# Maternal Chikungunya virus infection and pregnancy outcomes: a global systematic review and meta-analysis of vertical transmission dynamics and associated morbidity

**DOI:** 10.1080/22221751.2026.2651466

**Published:** 2026-03-25

**Authors:** Yan Guo, Yujie Xiang, Mengyuan Zheng, Zhouling Pan, Yanxian Jiang, Wei Chang, Gaowen Liu, Yonghan Luo, A.-Mei Zhang, Li Liu, Yuanyuan Zhang, Caifen Zhu, Jie Zhang, Yue Feng, Xueshan Xia

**Affiliations:** a Faculty of Life Science and Technology & Yunnan Province Key Laboratory of Public Health and Biosafety, Kunming University of Science and Technology, Kunming, Yunnan, People’s Republic of China; b Department of Reproductive Gynecology, NHC Key Laboratory of Healthy Birth and Birth Defect Prevention in Western China, First People's Hospital of Yunnan Province, Kunming, Yunnan, People’s Republic of China; c Department of Reproductive Gynecology, the Affiliated Hospital of Kunming University of Science and Technology, Kunming, Yunnan, People’s Republic of China; d Yunnan Kecan Biotechnology Co., Ltd, Kunming, Yunnan, People’s Republic of China; e The First Clinical Medical School, Kunming Medical University, Kunming, Yunnan, People’s Republic of China; f School of Public Health & Yunnan Province Key Laboratory of Public Health and Biosafety, Kunming Medical University, Kunming, Yunnan, People’s Republic of China

**Keywords:** Chikungunya virus, pregnancy, vertical transmission, adverse pregnancy outcomes, neonatal outcomes, meta-analysis

## Abstract

Chikungunya virus (CHIKV) infection during pregnancy presents a major threat to maternal-fetal health, yet a comprehensive global assessment of vertical transmission risks and perinatal outcomes remains scarce. To provide a comprehensive evidence synthesis, we conducted a systematic review and meta-analysis, prospectively registered in PROSPERO (CRD420251164423). We screened studies published up to October 20, 2025, and included 57 observational studies for qualitative synthesis, with 27 studies pooled in meta-analyses. The pooled vertical transmission rate was 18.1%, revealing a pronounced gestational-age gradient: rates were low in the first (3.9%) and second (1.2%) trimesters but surged dramatically during the third trimester (36.5%) and the intrapartum period (49.1%). Adverse pregnancy outcomes occurred in 11.3% of infected women, with abnormal fetal heart rate (44.9%) and stillbirth (22.0%) being the most frequent. Among neonates, 36.5% experienced adverse outcomes, commonly including feeding difficulties (79.4%), fever (68.9%), and thrombocytopenia (57.2%); neonatal mortality reached 6.9%. Crucially, meta-analysis of comparative studies demonstrated that maternal CHIKV infection was associated with a more than two-fold higher risk of adverse perinatal outcomes (OR = 2.28), with particularly robust associations identified for abnormal fetal heart rate (OR = 5.07) and delayed neurodevelopment (OR = 11.98). This study underscores that maternal CHIKV infection, especially during late gestation, substantially elevates the risks of vertical transmission and severe perinatal complications. Consequently, our findings strongly advocate for the implementation of enhanced prenatal surveillance and systematic postnatal assessment protocols in CHIKV-endemic regions to mitigate adverse outcomes and guide public health interventions.

## Background

Chikungunya virus (CHIKV) is an expanding mosquito-borne pathogen now posing an urgent global health threat [1]. By December 2025, more than 260,000 cases and 83 deaths had been reported across 12 countries [2]. The French overseas department of La Réunion in the Indian Ocean experienced a major outbreak in 2025. As of May 18, that region reported over 51,000 confirmed cases and more than 188,600 suspected cases [3]. Furthermore, autochthonous transmission has been reported in several temperate regions. By August 2025, Guangdong Province in China had documented approximately 8,000 locally acquired cases. Italy reported 167 autochthonous cases by early September [4], and France recorded 788 locally transmitted cases by the end of November [5,6]. These reports highlight the persistent and widespread threat of CHIKV in diverse geographical settings. Most infections cause acute fever, severe arthralgia, rash, and myalgia, and chronic joint symptoms may persist for months or years [7]. Although the overall fatality rate is low, severe outcomes and deaths occur disproportionately in neonates, infants, and older adults [8]. Evidence increasingly shows that maternal CHIKV infection can raise the risks of adverse obstetric outcomes, such as preterm birth, fetal growth restriction, abnormal fetal heart rate, and stillbirth [9–11]. Moreover, CHIKV can be vertically transmitted to the fetus [12], leading to neonatal fever, rash, thrombocytopenia [13], and in severe cases, life-threatening neurological complications such as encephalitis and meningitis with potential long-term neurodevelopmental sequelae [14,15]. Collectively, these epidemiological patterns demonstrate that CHIKV is emerging as a cross-continental and intensifying public health challenge, highlighting the urgent need for targeted interventions to safeguard maternal and child health.

Although previous systematic reviews and meta-analyses have explored the risks of CHIKV infection in pregnancy [16,17], they are often limited by methodological shortcomings. These include reliance on small-scale case reports, a lack of quantitative synthesis for key outcomes, inadequate subgroup analysis of important determinants such as gestational timing and geographic region, and a failure to directly compare outcomes between infected and uninfected women. Furthermore, the most recent syntheses predate 2018 and thus omit subsequent large-scale cohort studies, reducing the timeliness and representativeness of the available evidence.

To address these evidence gaps, we conducted a systematic review of 57 studies (2006–2025), including a meta-analysis of 27 studies that met predefined quality criteria. By incorporating recent large-scale cohort data and applying trimester-stratified analysis, our study shows that the risk of CHIKV vertical transmission increases with advancing pregnancy, with minimal risk in early pregnancy, a marked rise in the third trimester, and peak transmission around delivery. We also quantified the frequency of nearly 20 neonatal clinical manifestations and, based on six comparative cohort studies, provided the first direct evidence linking maternal CHIKV infection to adverse pregnancy and neonatal outcomes, suggesting a potential association with adverse outcomes. Sensitivity and publication bias analyses further support the robustness of these findings.

By synthesizing global evidence, this study provides timely and robust data to strengthen clinical guidance, guide public health responses, and enhance surveillance for CHIKV in pregnancy. Our findings directly inform the care of at-risk mothers and newborns, the design of targeted interventions, and strategic resource allocation in endemic regions, thereby advancing evidence-informed practice to mitigate this growing threat to maternal and child health worldwide.

## Methods

### Study design and registration

This systematic review and meta-analysis synthesizes evidence on the vertical transmission risk, pregnancy outcomes, and neonatal outcomes associated with maternal CHIKV infection. We followed the Preferred Reporting Items for Systematic Reviews and Meta-Analyses (PRISMA) guidelines [18] and prospectively registered this study in the International Prospective Register of Systematic Reviews (PROSPERO) with ID CRD420251164423.

### Search strategy

On October 20, 2025, we searched the PubMed, Web of Science, Embase, and the Cochrane Library for eligible studies without start date or language restrictions. The keywords were “Chikungunya virus,” “pregnancy,” “maternal infection,” “vertical transmission,” “congenital infection,” “fetus,” and “infant”. The full search strategy is described in detail in Table S1. In addition, reference lists of the relevant articles were manually checked for other potentially relevant papers.

Title and abstract screening and full-text evaluation were performed independently by two researchers (YG and YJX). Any disagreements during the screening process were resolved through discussion with a third researcher (YF).

### Inclusion and exclusion criteria

We included observational studies (cohort, case–control, and cross-sectional), case series, and reports involving pregnant women with CHIKV infection. Eligible studies were required to report at least one outcome related to neonatal CHIKV infection, pregnancy (e.g. preterm birth, miscarriage, preeclampsia), or neonatal health (e.g. fever, rash, meningitis).

We excluded studies with incomplete or unclear pregnancy or transmission data; those focusing on neonates or children unrelated to maternal infection; studies involving co-infections with other pathogens (including, but not limited to, other arboviruses such as dengue virus and Zika virus) that prevented isolation of the specific effect of CHIKV; those lacking laboratory confirmation of fetal or neonatal CHIKV infection; and non-human, non-original, or inaccessible studies (e.g. in vitro, animal, review, or unpublished conference abstracts).

For quantitative meta-analysis, we included cohort studies, cross-sectional studies, and case series with at least five cases to ensure sufficient data for meaningful statistical pooling and to reduce instability caused by extremely small samples. Case reports and smaller case series (< 5 cases) were included only in the descriptive synthesis [17,19,20].

### Data extraction and quality assessment

Data extraction was performed independently by two investigators (YG and YJX) using a standardized form that captured study characteristics, methodological details, and outcome measures. A third investigator (YF) verified the extracted data, and any discrepancies were resolved through consensus.

Methodological quality was assessed using design-specific tools. For case–control and cohort studies, the Newcastle-Ottawa Scale (NOS) was applied [21], evaluating selection, comparability, and exposure/outcome assessment, with total scores (0–9) determining quality categories (low: 1–3, moderate: 4–6, high: 7–9). For single-arm cohort studies, comparability was omitted. Case series and reports were evaluated using the Murad tool [22], which covers selection, ascertainment, causality, and reporting; studies were rated based on the number of criteria met (high: 7, moderate: 5–6, low: < 5). Two reviewers (YG, YJX) conducted independent assessments, with a third reviewer (YF) resolving any discrepancies through consensus.

### Handling of overlapping or duplicate data

To minimize the risk of double-counting, all included studies were screened for potential overlap by comparing study location, recruitment period, author groups, and sample sizes. When multiple publications originated from the same cohort or outbreak dataset (e.g. secondary analyses, extended follow-up reports, or subgroup analyses), the report providing the most comprehensive dataset or the largest sample size was prioritized for quantitative synthesis. Additional publications derived from the same cohort were retained for qualitative synthesis only when they reported unique outcomes not available in the primary report.

When different outcomes were reported across multiple publications from the same cohort, outcome-specific data were extracted while ensuring that individual participants were not counted more than once in pooled analyses. Detailed procedures for study selection and data handling are provided in the Supplementary Methods.

### Outcomes

This systematic review and meta-analysis of 27 studies evaluated outcomes following maternal CHIKV infection. The primary outcome was vertical transmission of CHIKV from mother to infant, defined according to the laboratory-confirmed diagnostic criteria reported in each individual study (e.g. RT–PCR detection of viral RNA or serological evidence of neonatal IgM antibodies). Because diagnostic approaches varied across studies, the original definitions were retained during data extraction and harmonized for quantitative synthesis. Secondary outcomes encompassed adverse pregnancy outcomes (e.g. miscarriage, preterm birth, stillbirth) and neonatal outcomes (e.g. systemic symptoms, neurological/hematological abnormalities, respiratory complications, death). For neonatal outcomes, a composite category of “at least one adverse neonatal outcome” was constructed, comprising clinical symptoms, laboratory abnormalities, imaging findings, and severe complications based on harmonized definitions across studies. Due to heterogeneity in outcome definitions, data were extracted as reported and harmonized into broader prespecified categories for synthesis. Detailed harmonization procedures are provided in the Supplementary Methods, and a complete mapping of outcome definitions across studies is available in Supplementary Table S10.

### Statistical analysis

All quantitative syntheses were conducted using random-effects models to account for expected clinical and methodological heterogeneity across studies. Pooled estimates with 95% confidence intervals (CIs) were calculated for proportions and odds ratios (ORs) of dichotomous outcomes. Statistical heterogeneity was assessed using the *I^2^* statistic [23], with values >75% considered indicative of substantial heterogeneity. Potential sources of heterogeneity were explored through subgroup analyses (e.g. gestational stage of infection and geographic region) and sensitivity analyses, including leave-one-out analyses performed by sequentially excluding individual studies and analyses restricted to studies with molecular confirmation of neonatal infection (RT–PCR alone or combined with serology). Because outcome definitions varied across studies, data were extracted using the original study definitions and grouped into broader conceptual categories for synthesis. Publication bias was assessed using funnel plots with Egger’s and Begg’s tests. Statistical significance for odds ratios (ORs) was assessed based on whether the 95% confidence interval excluded the null value (1.0); ORs with 95% CIs not crossing 1.0 were considered statistically significant. All *p*-values were two-tailed (significant at *p* < 0.05). Analyses were conducted using the meta package (version 6.5-1) in R (version 3.4.2) [24], which is consistent with the methodological standards for meta-analyses as described in the CRAN documentation (https://cran.r-project.org/web/packages/meta/index.html).

## Results

### Study selection and characteristics

A total of 57 studies met the inclusion criteria for the systematic review, of which 27 were eligible for quantitative meta-analysis.

**Figure 1.**
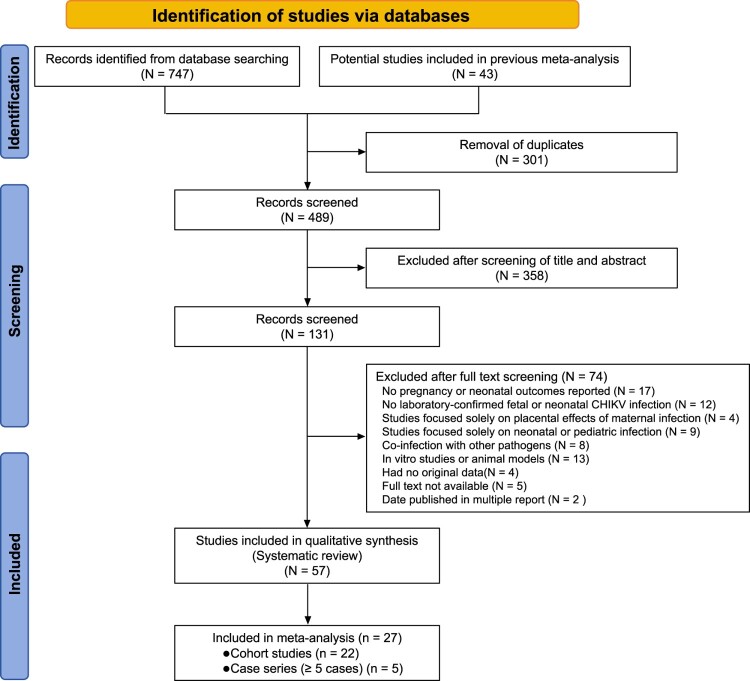
PRISMA flow diagram of study selection. The diagram illustrates the identification, screening, eligibility assessment, and inclusion of studies in the systematic review and meta-analysis.

From 747 database records and 43 additional sources, 301 duplicates were removed. Title and abstract screening of 489 records led to full-text review of 131 articles. Ultimately, 57 studies met the inclusion criteria and were included in the systematic review, with 27 studies (cohorts and case series with ≥ 5 cases) eligible for meta-analysis (Figure 1).

The included studies were published between 2006 and 2025 and covered multiple geographic regions, primarily Reunion Island, Brazil, India, and Colombia. In terms of study design, 22 studies (38.6%) were cohort studies and 35 (61.4%) were case series or case reports. CHIKV infection was most commonly diagnosed via RT–PCR (40 studies, 70.2%). Serological assays were used in 28 studies (49.1%), and 11 studies (19.3%) combined RT–PCR with serology. Maternal infection occurred across all stages of pregnancy, with late-pregnancy infections most frequently reported (Table S2).

### Risk of bias and quality assessment

Study quality was assessed using design-specific validated tools, including the NOS for observational studies and the Murad tool for case series and case reports, as described in the Methods section. Overall, the methodological quality of included studies was moderate to high. Among the 22 observational studies, 18 (81.8%) were rated as high quality and 4 (18.2%) as moderate quality. The main limitations involved comparability and completeness of reporting. Only 7 studies (31.8%) included control groups for confounder adjustment. Two studies (9.1%) had incomplete baseline data; however, they were retained because they met predefined inclusion criteria and provided sufficient outcome data for quantitative synthesis. Sensitivity analyses showed that their inclusion did not materially affect the pooled estimates (Tables S2, S3).

Of 35 case reports and series, 33 (94.3%) were rated as high quality. Limitations mainly concerned reporting completeness: 2 studies (5.7%) had incomplete baseline data, and among the 19 case series, only 11 (57.9%) adequately described statistical methods (Tables S2, S3).

### Qualitative synthesis of included studies

Among 57 studies, 48 reported laboratory-confirmed mother-to-child transmission data. Evidence consistently indicates that vertical transmission risk is strongly associated with gestational timing, occurring predominantly during late pregnancy or the intrapartum period, as reported in studies from Réunion Island, Colombia, Brazil, India, and Thailand. For instance, transmission rates reached 28–48% in cohorts from these regions [24,25]. In contrast, early to mid-pregnancy infections rarely resulted in confirmed vertical transmission [9,10,26], suggesting the placental barrier may be more vulnerable in later gestation (Table S2).

**Figure 2.**
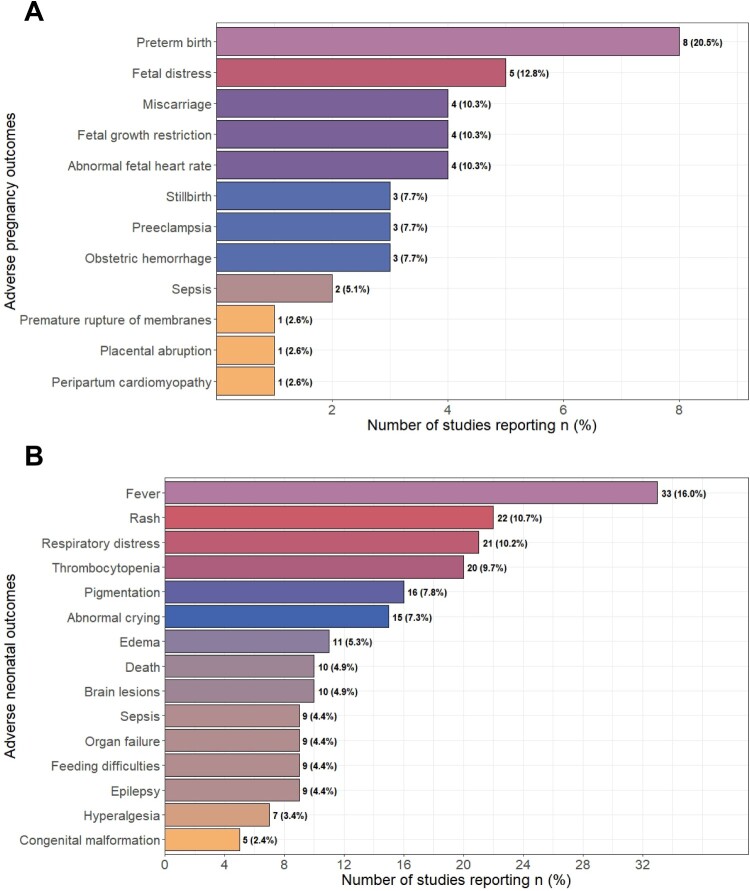
Summary of systematic review findings. Adverse pregnancy outcomes were reported across the included studies. (B) Adverse neonatal outcomes reported in neonates exposed to maternal CHIKV infection. “n” indicates the number of studies reporting each outcome, with the percentage representing the proportion of included studies that reported that specific outcome.

In studies reporting adverse pregnancy outcomes (26 studies), preterm birth was the most frequently reported outcome (8 studies), followed by fetal distress (5 studies), abnormal fetal heart rate (4 studies), obstetric hemorrhage (3 studies), miscarriage (3 studies), and stillbirth (3 studies). Current evidence suggests a stronger association between acute maternal infection in late pregnancy and these obstetric complications, particularly preterm birth and fetal distress (Figure 2A, Table S2).

In 51 studies reporting neonatal outcomes, the most common clinical features were fever (33 studies), rash (22 studies), and thrombocytopenia (20 studies). A significant proportion of neonates developed severe complications, including neurological involvement (19 studies) and respiratory distress (21 studies). Fatal cases were reported in 10 studies, highlighting the potential lethality of severe neonatal CHIKV infection (Figure 2B, Table S2).

## Vertical transmission risk

### Overall vertical transmission rate

**Figure 3.**
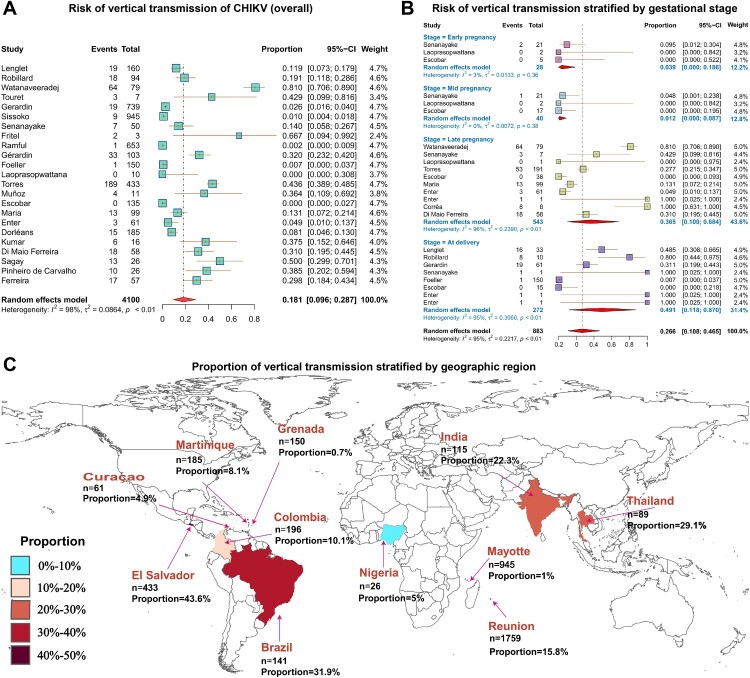
Risk of vertical transmission of CHIKV during pregnancy. (A) Overall pooled risk of vertical transmission among pregnant women infected with CHIKV. (B) Risk of vertical transmission stratified by gestational stage (early pregnancy, mid pregnancy, late pregnancy, and at delivery). (C) Geographic distribution of vertical transmission proportions across regions included in the analysis. In panels A and B, squares represent the proportion reported in individual studies and horizontal lines indicate 95% confidence intervals (CIs). The size of each square reflects the weight of the study in the meta-analysis. Diamonds represent pooled estimates derived from random-effects meta-analysis. In panel C, colours indicate the proportion of vertical transmission in each region, and n represents the total number of infected pregnancies included in the regional analysis.

A total of 24 studies involving 4,100 CHIKV-infected pregnant women were included in the quantitative analysis of vertical transmission. The pooled analysis demonstrated that nearly one in five pregnancies affected by maternal CHIKV infection resulted in vertical transmission, with an overall rate of 18.1% (95% CI: 9.6% – 28.7%, *I^2^* = 98%), indicating a substantial risk of mother-to-child infection. Substantial between-study heterogeneity was observed, likely reflecting differences in outbreak settings, diagnostic approaches, and the timing of maternal infection (Figure 3A).

### Stratified analysis by gestational stage

Subgroup analysis based on the trimester of infection revealed that the risk of vertical transmission was significantly associated with the timing of maternal infection (Figure 3B; Table S4). The estimated transmission rates following infection during the first and second trimesters were 3.9% (95% CI: 0.0% – 18.6%, *I^2^* = 3%) and 1.2% (95% CI: 0.0% – 8.7%, *I^2^* = 0%), respectively. However, these estimates were based on limited data and wide confidence intervals. In stark contrast, the risk increased markedly with third-trimester infection, reaching 36.5% (95% CI: 10.0% – 68.4%, *I^2^* = 96%), and was highest for intrapartum infection, with a transmission rate of 49.1% (95% CI: 11.8% – 87.0%, *I^2^* = 95%). These results demonstrate a clear gestational-age gradient in transmission risk, with minimal transmission in early pregnancy but a dramatic increase during late pregnancy and particularly around delivery.

### Stratified analysis by geographic region

Subgroup analyses by geographical region demonstrated substantial heterogeneity in the vertical transmission rate of CHIKV (Figure 3C, S1, Table S5). In Réunion Island, where seven studies were available, the pooled vertical transmission rate was 15.8% (95% CI: 4.1% – 33.1%, *I²* = 97%). In contrast, estimates from Brazil, based on three studies, were considerably higher at 31.9% (95% CI: 24.5% – 39.8%, *I²* = 0%). Several regions showed widely divergent vertical transmission rates based on single-study estimates, ranging from 0.7% in Grenada and 1.0% in Mayotte to 43.6% in El Salvador and 50.0% in Nigeria. Overall, these findings highlight pronounced geographic variability in CHIKV vertical transmission risk, suggesting that regional epidemiological conditions, diagnostic capacity, and healthcare infrastructure may strongly influence reported transmission rates.

## Adverse pregnancy outcomes

### Overall incidence

The combined result showed that the incidence of at least one adverse pregnancy outcome in CHIKV-infected pregnant women was 11.3% (95% CI: 6.9% – 16.7%, *I²* = 85%) (Figure S2, Table S6). This indicates that more than one in ten pregnancies complicated by maternal CHIKV infection resulted in at least one adverse obstetric outcome.

### Outcome-specific analysis

**Figure 4.**
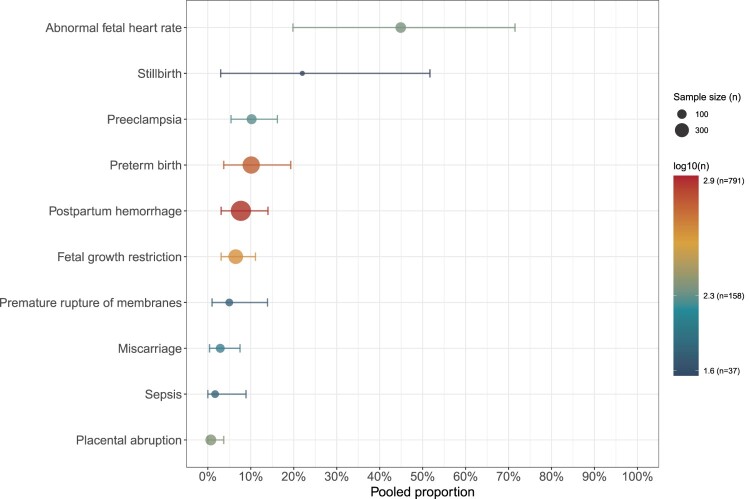
Pooled proportions of adverse pregnancy outcomes following maternal CHIKV infection. Each point represents the pooled proportion of a specific pregnancy outcome estimated using random-effects meta-analysis, with horizontal lines indicating 95% confidence intervals. The x-axis represents the pooled proportion (percentage) of CHIKV-infected pregnant women experiencing each outcome. The size of each point corresponds to the sample size (n) included in the meta-analysis for that outcome, and the colour scale represents the log-transformed sample size (log10[*n*]).

Subgroup analysis based on different pregnancy outcomes showed significant differences in the incidence of various adverse pregnancy outcomes. Abnormal fetal heart rate was the most commonly reported adverse outcome, with an incidence of 44.9% (95% CI: 19.8% – 71.5%, *I²* = 89%). The incidence of stillbirth was 22.0% (95% CI: 3.0% – 51.7%, *I²* = 72%). Other notable outcomes included preterm birth (10.1%), preeclampsia (10.2%), and postpartum hemorrhage (7.7%), while fetal growth restriction (6.5%) and miscarriage (2.9%) occurred less commonly. Individual studies also reported premature rupture of membranes (5.0%), sepsis (1.7%), and placental abruption (0.7%) (Figure 4, S2, Table S6). Taken together, these findings indicate that maternal CHIKV infection is associated with a broad spectrum of obstetric complications, with fetal cardiac abnormalities and stillbirth representing the most prominent risks.

## Adverse neonatal outcomes

### Overall incidence

A pooled analysis showed that the combined proportion of neonates born to CHIKV-infected mothers who experienced at least one adverse outcome was 36.5% (95% CI: 30.0% – 43.2%, *I²* = 96%) (Figure S3, Table S7). This proportion suggests that neonatal morbidity following maternal CHIKV infection is substantial. The composite outcome encompassed a broad spectrum of neonatal morbidity as prespecified in the Methods.

### Outcome-specific analysis

**Figure 5.**
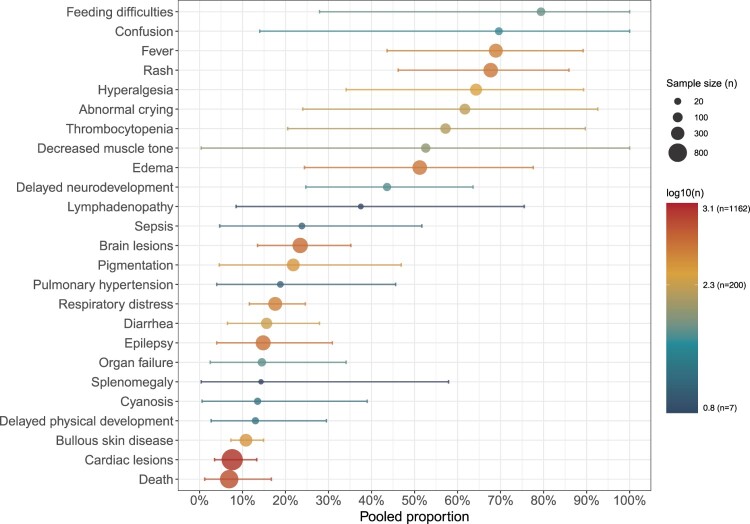
Pooled proportions of adverse neonatal outcomes among neonates exposed to maternal CHIKV infection. Each point represents the pooled proportion of neonates presenting with a specific clinical outcome, estimated using random-effects meta-analysis. Horizontal lines indicate 95% confidence intervals. The x-axis represents the pooled proportion (percentage) of neonates with each outcome. Point size reflects the sample size (n) included in the analysis for that outcome, and the colour scale represents the log-transformed sample size (log10[*n*]).

The incidence of various adverse neonatal outcomes differed substantially. Among systemic symptoms, feeding difficulties had the highest incidence at 79.4% (95% CI: 27.9% – 100.0%, *I²* = 94%), followed by fever (68.9%; 95% CI: 43.6% – 89.2%, *I²* = 96%) and rash (67.7%; 95% CI: 46.2% – 85.9%, *I²* = 95%). Hyperalgesia (64.3%; 95% CI: 34.1% – 89.3%, *I²* = 95%) and abnormal crying (61.7%; 95% CI: 24.0% – 92.6%, *I²* = 96%) were also frequently observed. For neurological complications, the incidence of decreased muscle tone was 52.6% (95% CI: 0.4% – 100.0%, *I²* = 96%), brain lesions 23.4% (95% CI: 13.5% – 35.2%, *I²* = 81%), and epilepsy 14.8% (95% CI: 4.0% – 30.9%, *I²* = 91%). Among hematological abnormalities, thrombocytopenia was most prominent with an incidence of 57.2% (95% CI: 20.5% – 89.7%, *I²* = 95%). For respiratory complications, respiratory distress occurred in 17.6% (95% CI: 11.6% – 24.6%, *I²* = 53%). Among severe outcomes, 16 deaths were reported among 787 neonates across five studies included in the pooled analysis, corresponding to a mortality rate of 6.9% (95% CI: 1.2% – 16.7%, *I²* = 82%). Analysis based on single-study reports showed the incidence of lymphadenopathy was 37.5%, pulmonary hypertension 18.8%, and splenomegaly 14.3% (Figure 5, S3, Table S7). These results indicate that neonatal CHIKV infection frequently manifests with systemic symptoms and neurological complications, with hematological abnormalities such as thrombocytopenia also commonly observed.

## Comparative analysis between infected and non-infected pregnancies

### Overall increased risk

In a meta-analysis of six comparative cohort studies, CHIKV-infected women had significantly higher overall odds of adverse pregnancy and neonatal outcomes compared to non-infected women (OR = 2.28, 95% CI: 1.35–3.86, *I²* = 80%) (Figures S4-S6, Table S8). This finding indicates that maternal CHIKV infection was associated with approximately twofold higher odds of adverse perinatal outcomes.

### Outcome-specific associations

**Figure 6.**
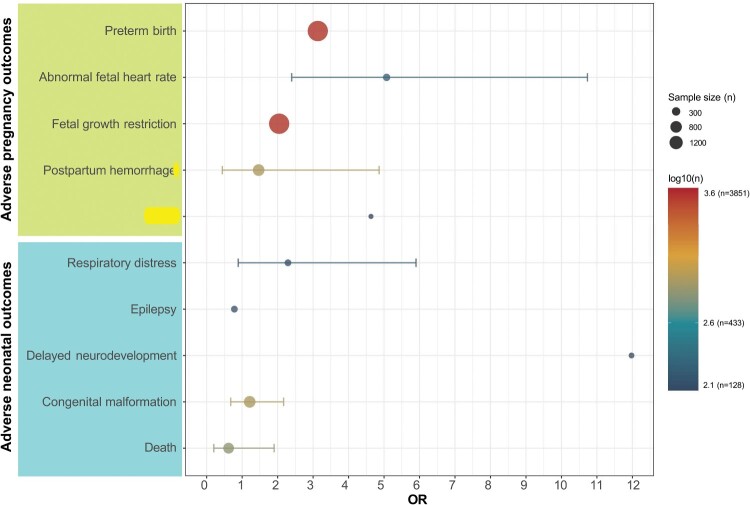
Comparison of adverse pregnancy and neonatal outcomes between CHIKV-infected and uninfected pregnant women. The figure presents pooled odds ratios (ORs) comparing the risk of selected pregnancy and neonatal outcomes between women with and without CHIKV infection. Horizontal lines represent 95% confidence intervals derived from random-effects meta-analysis. The x-axis represents the pooled odds ratio (OR). Outcomes are grouped into adverse pregnancy outcomes (upper panel) and adverse neonatal outcomes (lower panel). The size of each point corresponds to the sample size (n) included in the analysis, and the colour scale represents the log-transformed sample size (log10[*n*]).

Abnormal fetal heart rate (OR = 5.073, 95% CI: 2.397–10.735, *I²* = 0%) and delayed neurodevelopment (OR = 11.98, 95% CI: 4.78–30.05) were significantly more frequent in infected women. These findings suggest a possible association between maternal CHIKV infection and fetal cardiac abnormalities as well as neurodevelopmental outcomes. Elevated but non-significant odds were observed for preterm birth (OR = 3.13) and fetal growth restriction (OR = 2.04). No significant differences were found for other outcomes, including postpartum hemorrhage, fever, respiratory distress, congenital malformations, death, or epilepsy (Figure 6, S4 – S6, Table S8). In summary, CHIKV infection was specifically associated with increased risks of abnormal fetal heart rate and neurodevelopmental delay, while other outcome trends were not statistically significant. However, several outcome-specific estimates were derived from a limited number of studies, and these findings should therefore be interpreted with caution.

## Sensitivity analysis

Sensitivity analyses demonstrated stable and robust pooled estimates across analyses. Leave-one-out sensitivity analyses, performed by sequentially excluding individual studies, showed that no single study materially influenced the pooled estimates for vertical transmission, pregnancy outcomes, neonatal outcomes, or comparative analyses (Figures S7, S10, S11, S13, S15). In addition, sensitivity analyses restricted to studies with molecular confirmation of neonatal infection (RT–PCR alone or combined with serology) yielded pooled estimates comparable to the primary analysis (Figure S9).

## Publication bias

Egger’s and Begg’s tests indicated no significant publication bias across all outcome categories, including vertical transmission (*p* = 0.39 and 0.46), adverse pregnancy outcomes (*p* = 0.28 and 0.31), adverse neonatal outcomes (*p* = 0.07 and 0.11), and comparative risks between groups (*p* = 0.07 and 0.15). Funnel plots were generally symmetrical, supporting a low overall risk of publication bias and the robustness of the findings (Figures S8, S12, S14, S16, Table S9).

## Discussion

This global systematic review and meta-analysis of 57 studies (2006–2025) suggests that maternal CHIKV infection is associated with substantial risks for adverse pregnancy and neonatal outcomes. The pooled vertical transmission rate was 18.1%, with the estimated transmission risk increasing from early pregnancy to late gestation and reaching its highest level around delivery. Importantly, the limited number of early-pregnancy cases and the wide confidence intervals in pooled estimates mean that a low observed transmission rate should not be interpreted as the absence of risk. Adverse obstetric complications and neonatal morbidity were also frequently reported, and comparative analyses suggested higher odds of adverse perinatal outcomes among infected women.

This temporal pattern may reflect gestational changes in placental barrier function that have been hypothesized to facilitate maternal-fetal viral transfer. In early gestation, the placenta is characterized by a relatively thick multilayered trophoblast barrier that may restrict viral passage from the maternal to the fetal circulation [27–29]. As pregnancy progresses, structural and functional adaptations of the placenta, including syncytiotrophoblast thinning and increased placental vascularization [30], enhance maternal-fetal exchange but may simultaneously increase vulnerability to viral dissemination across the placental interface [31], which has been proposed as a possible explanation for the higher transmission rates reported in late pregnancy (36.5%). In addition to transplacental transmission during late gestation, infection around delivery may occur through direct neonatal exposure to infected maternal blood and secretions, effectively bypassing the placental barrier. These mechanistic differences across gestational stages provide a biological basis for clinical risk stratification. In particular, maternal infection in late pregnancy may warrant intensified fetal surveillance and delivery planning in facilities equipped with neonatal intensive care capability, whereas infections occurring in early pregnancy appear less likely to require invasive intervention. However, these biological explanations should therefore be interpreted as hypothesis-generating interpretations based on existing experimental and clinical literature rather than direct evidence derived from the present meta-analysis. Future research integrating placental histopathology, virology, and clinical observations will be essential to better define the temporal windows of placental susceptibility and to inform targeted prevention strategies.

Another important observation of this study is the substantial geographic variability in vertical transmission risk across regions. Reported estimates ranged widely across outbreak settings, indicating that global pooled estimates should be interpreted cautiously. This heterogeneity may be influenced by multiple factors. Although viral genetic variation has been proposed as a potential contributor to differences in transmission dynamics, direct evidence linking specific CHIKV strains or lineages to differential mother-to-child transmission risk remains limited, as genomic sequencing is rarely performed in such settings. More consistently, variability in surveillance systems, diagnostic capacity, outbreak intensity, and healthcare infrastructure likely plays a substantial role in shaping the observed regional differences [32]. Consequently, prevention and response strategies should be tailored to regional healthcare capacities and epidemiological contexts rather than relying on a uniform global risk estimate.

Maternal CHIKV infection was also associated with a range of obstetric complications. Abnormal fetal heart rate emerged as the most frequently reported obstetric manifestation, suggesting that maternal infection may directly or indirectly disrupt fetal cardiac function. Evidence suggests CHIKV can infect fetal myocardium through vertical transmission, causing myocardial injury and structural abnormalities [31]. Viral infection may also indirectly affect fetal heart development via maternal systemic inflammatory responses [33]. The occurrence of myocarditis in CHIKV infections further supports that the virus can impair fetal cardiac function through direct or indirect mechanisms [34]. The occurrence of stillbirth further underscores the potential severity of intrauterine infection. Severe maternal viremia, placental damage, or acute impairment of fetal circulation may contribute to fetal demise in severe cases [9]. These findings emphasize the importance of fetal monitoring in pregnancies complicated by CHIKV infection, particularly during late gestation.

Neonatal outcomes also demonstrate that CHIKV infection can involve multiple organ systems. A substantial proportion of exposed neonates developed clinical complications, reflecting the systemic nature of neonatal CHIKV infection. The proportion of neonates with at least one adverse outcome in our analysis (36.5%) is higher than the pooled risk of symptomatic neonatal disease reported in previous meta-analyses [17]. This discrepancy likely reflects differences in outcome definitions. In our study, adverse neonatal outcomes were defined using a harmonized composite framework that encompassed systemic symptoms, laboratory abnormalities, imaging findings, and severe complications, rather than only acute symptomatic disease. This broader scope may account for the higher overall proportion observed. Early manifestations are often nonspecific, including fever, rash, feeding difficulties, and irritability, which may represent the initial phase of systemic viral dissemination. However, a proportion of affected infants subsequently develop severe complications, particularly neurological involvement such as encephalitis or brain parenchymal injury, highlighting the neuroinvasive potential of the virus [35,36]. Hematological abnormalities are also frequently observed, particularly thrombocytopenia, which may result from direct viral injury to megakaryocytes or immune-mediated platelet destruction [37]. Respiratory distress and mortality were observed. The mortality estimate exceeds that reported in earlier meta-analyses [17] and may reflect several methodological and epidemiological differences. Our review incorporated more recent outbreak data (2006–2025), including studies from regions with heterogeneous healthcare capacity where case-fatality rates may vary. In addition, the pooled estimate was largely derived from neonates with laboratory-confirmed vertical or perinatal infection or clinically significant disease, thereby representing a higher-risk subgroup rather than all infected pregnancies. Wider adoption of RT–PCR and improved diagnostic capacity in recent outbreaks may also have increased identification of severe neonatal cases. Finally, substantial inter-study heterogeneity and the inclusion of hospital-based cohorts during epidemic peaks may have influenced the pooled estimate. These findings underscore the importance of close monitoring of neonates exposed to maternal CHIKV infection, even when early symptoms appear mild.

Our comparative meta-analysis suggests that maternal CHIKV infection is associated with higher odds of adverse pregnancy and neonatal outcomes. The significantly elevated risks of abnormal fetal heart rate and delayed neurodevelopment suggest that CHIKV infection may affect both acute fetal physiology and long-term neurological development. It should be noted that the estimate for delayed neurodevelopment was derived from a very limited number of studies, while the association with abnormal fetal heart rate was based on only two studies. Moreover, neurodevelopmental outcomes were assessed using heterogeneous follow-up durations and assessment tools across studies. Therefore, these findings should be interpreted with caution. Future prospective studies with standardized neurodevelopmental assessments and long-term follow-up are needed to better clarify these associations. Nonetheless, these findings highlight the importance of extended neurodevelopmental follow-up for infants exposed to CHIKV during pregnancy, particularly those infected in the perinatal period.

However, several limitations should be considered when interpreting our findings. First, most included studies were observational in design, precluding causal inference and leaving residual confounding unavoidable despite comparative analyses. In particular, the comparative meta-analysis was based on only six cohort studies, several of which had limited adjustment for key confounders. Second, substantial heterogeneity was observed across most pooled estimates, likely reflecting differences in study design, outbreak settings, geographic regions, timing of maternal infection, as well as diagnostic methods and outcome definitions. Although viral lineage variation has been proposed as a potential contributor to differences in transmission dynamics and disease severity, genome sequencing data were unavailable for the vast majority of included studies and could not be analyzed. Third, several outcome-specific estimates, including neonatal mortality and stillbirth, were based on a limited number of studies and events, resulting in wide confidence intervals and reduced precision. In addition, geographic representation was uneven, and long-term neurodevelopmental follow-up data remain scarce. Therefore, these pooled estimates should be interpreted as indicators of overall trends rather than precise risk estimates for specific populations. Large prospective multicenter studies with standardized protocols and longer follow-up are needed to strengthen the evidence base.

Despite these limitations, this study provides the most comprehensive synthesis to date of the global evidence on maternal CHIKV infection and pregnancy outcomes. By integrating data from multiple outbreak regions and incorporating recent large-scale cohort studies, our analysis offers a robust overview of the clinical spectrum and transmission dynamics of CHIKV during pregnancy.

In summary, maternal CHIKV infection, particularly during late gestation, poses significant risks to both maternal and neonatal health. The pronounced gestational-age gradient in transmission, the broad spectrum of neonatal complications, and the independent association with adverse perinatal outcomes highlight the need for risk-stratified clinical surveillance in CHIKV-endemic regions. For pregnancies complicated by third-trimester maternal infection, enhanced monitoring may include serial assessment of fetal heart rate and ultrasound-based evaluation of fetal growth and well-being, with delivery planned in facilities equipped with neonatal intensive care capability. Neonates exposed during late pregnancy or around delivery should undergo close early observation and laboratory monitoring for potential neurological or hematological complications. Future research should prioritize prospective multicenter cohort studies with integrated virological, placental, and clinical data to better define the mechanisms and timing of vertical transmission. Studies investigating placental viral tropism and maternal immune responses are also needed to clarify the biological pathways underlying maternal-fetal infection. In addition, the development and evaluation of maternal CHIKV vaccines represent an important research priority to reduce the risk of infection during pregnancy and improve neonatal outcomes.

## Supplementary Material

FigureS8.pdf

FigureS1.pdf

FigureS11.pdf

FigureS10.pdf

FigureS3.pdf

FigureS12.pdf

FigureS2.pdf

FigureS7.pdf

FigureS9.pdf

Appendix.pdf

FigureS4.pdf

FigureS13.pdf

FigureS5.pdf

FigureS14.pdf

FigureS6.pdf

## Data Availability

All data is provided in the article/supplementary materials.
