# Supplementary figures and images for: Maternal Chikungunya virus infection and pregnancy outcomes: a global systematic review and meta-analysis of vertical transmission dynamics and associated morbidity
Source: Emerg Microbes Infect. 2026 Mar 25;15(1):2651466. doi: 10.1080/22221751.2026.2651466 (PMC13101005; doi:10.1080/22221751.2026.2651466)

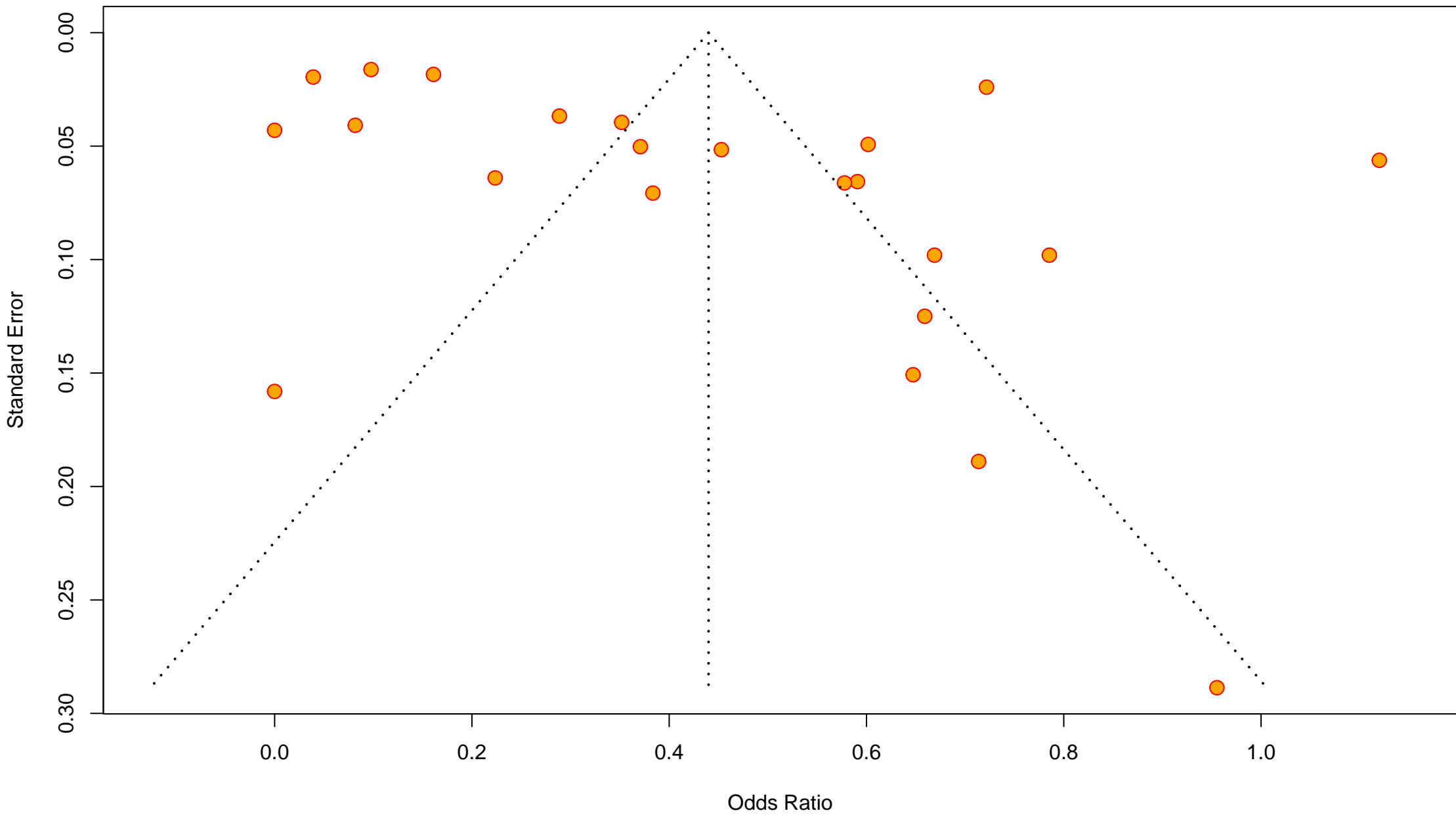

Supplement: FigureS8.pdf [file TEMI_A_2651466_SM5573.pdf]

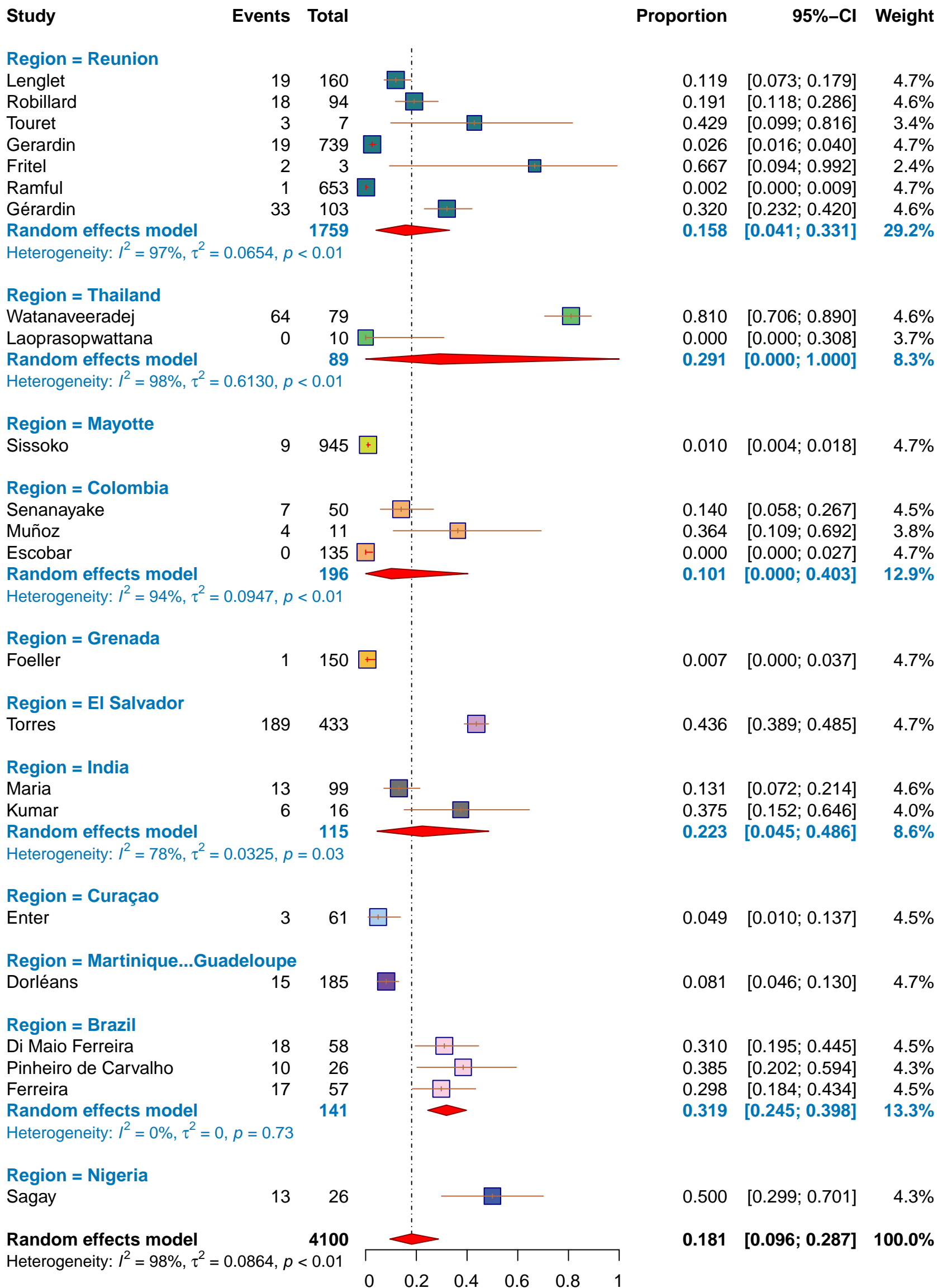

Supplement: FigureS1.pdf [file TEMI_A_2651466_SM5572.pdf]

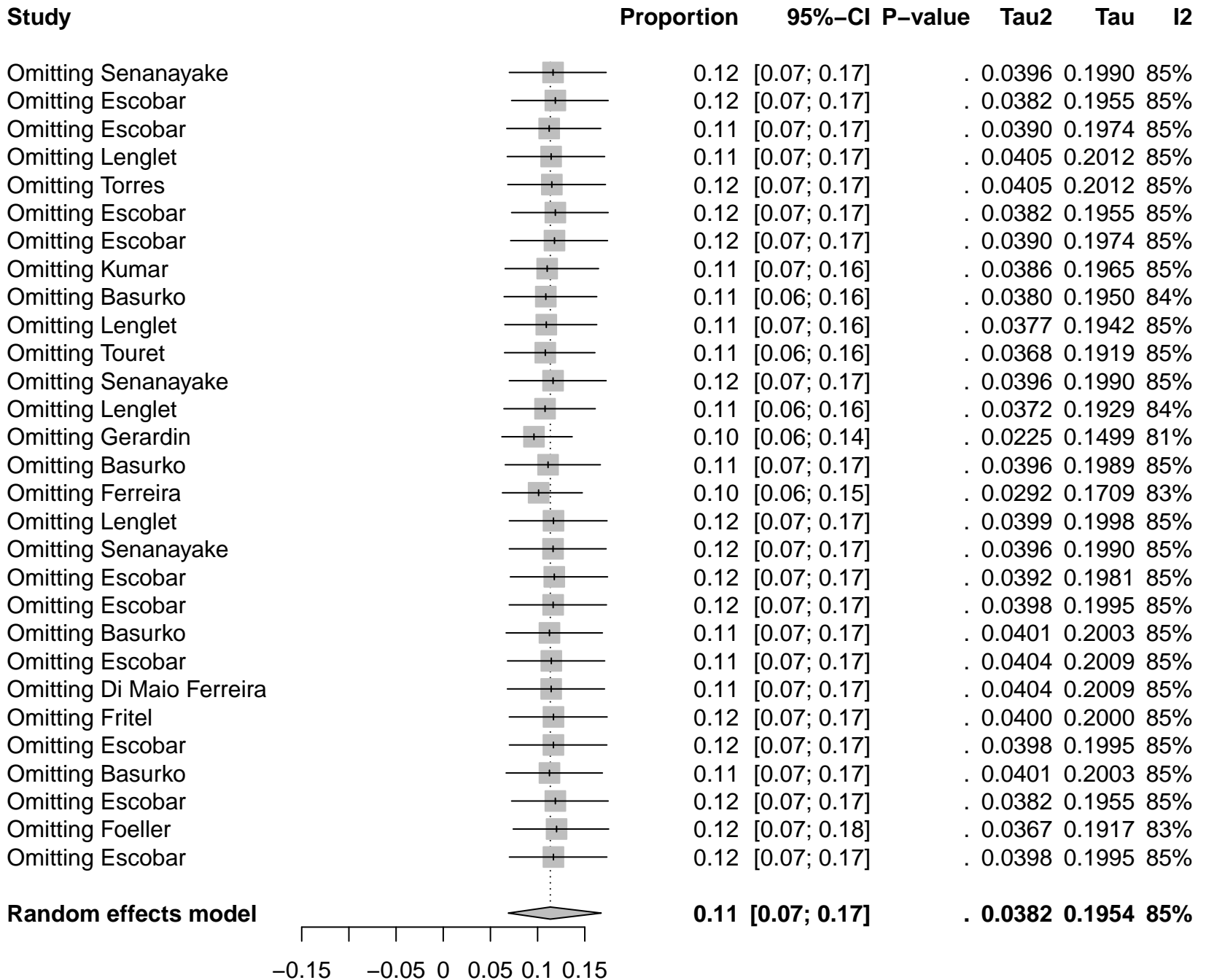

Supplement: FigureS11.pdf [file TEMI_A_2651466_SM5571.pdf]

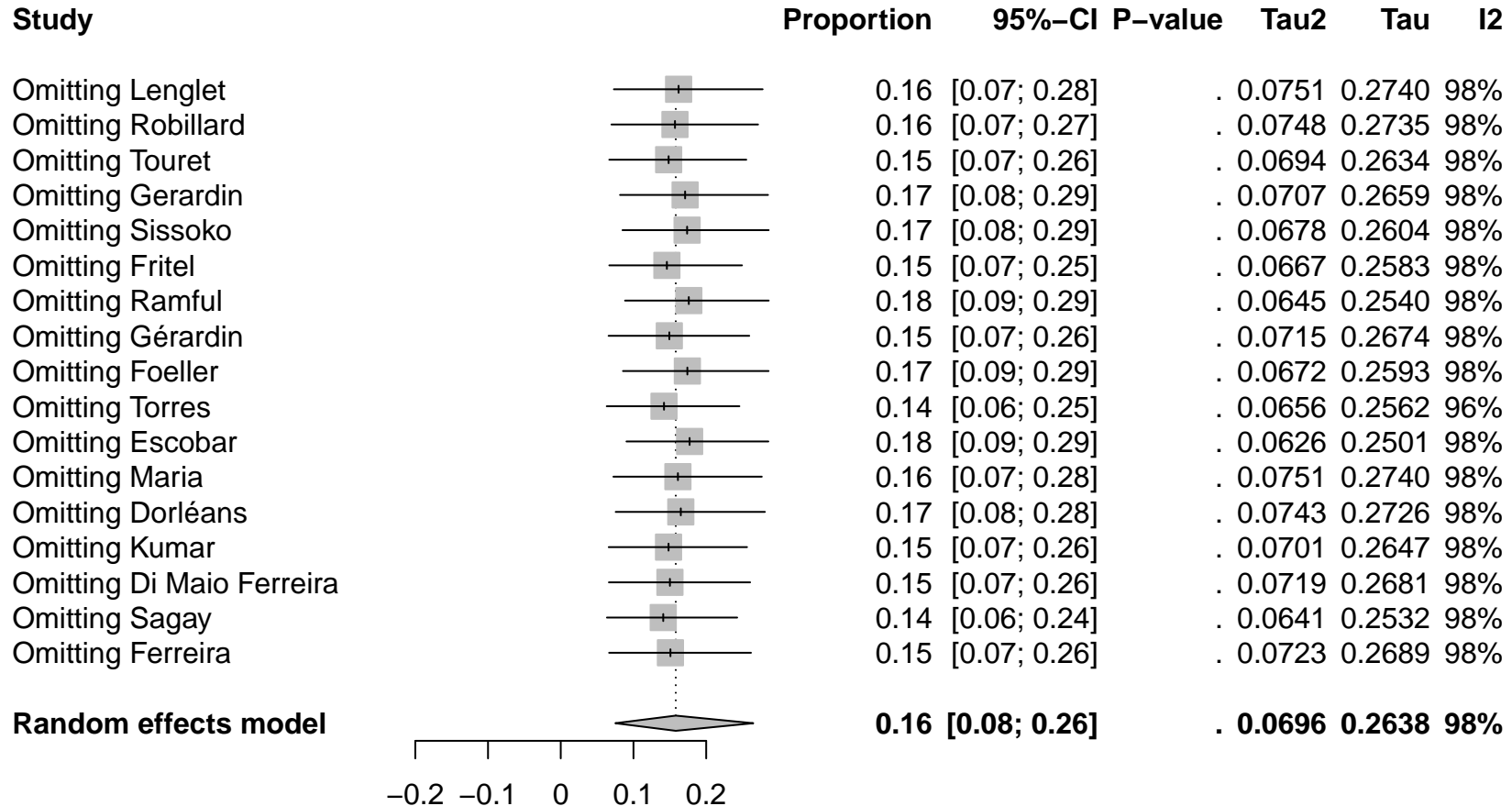

Supplement: FigureS10.pdf [file TEMI_A_2651466_SM5570.pdf]

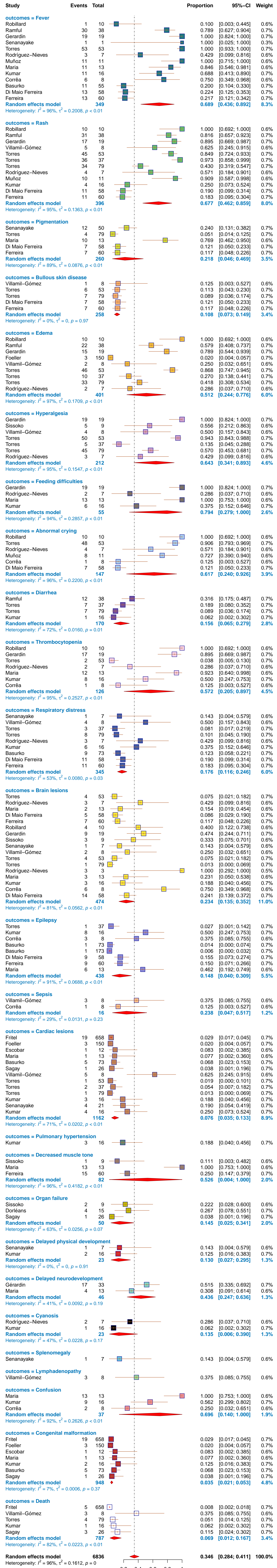

Supplement: FigureS3.pdf [file TEMI_A_2651466_SM5569.pdf]

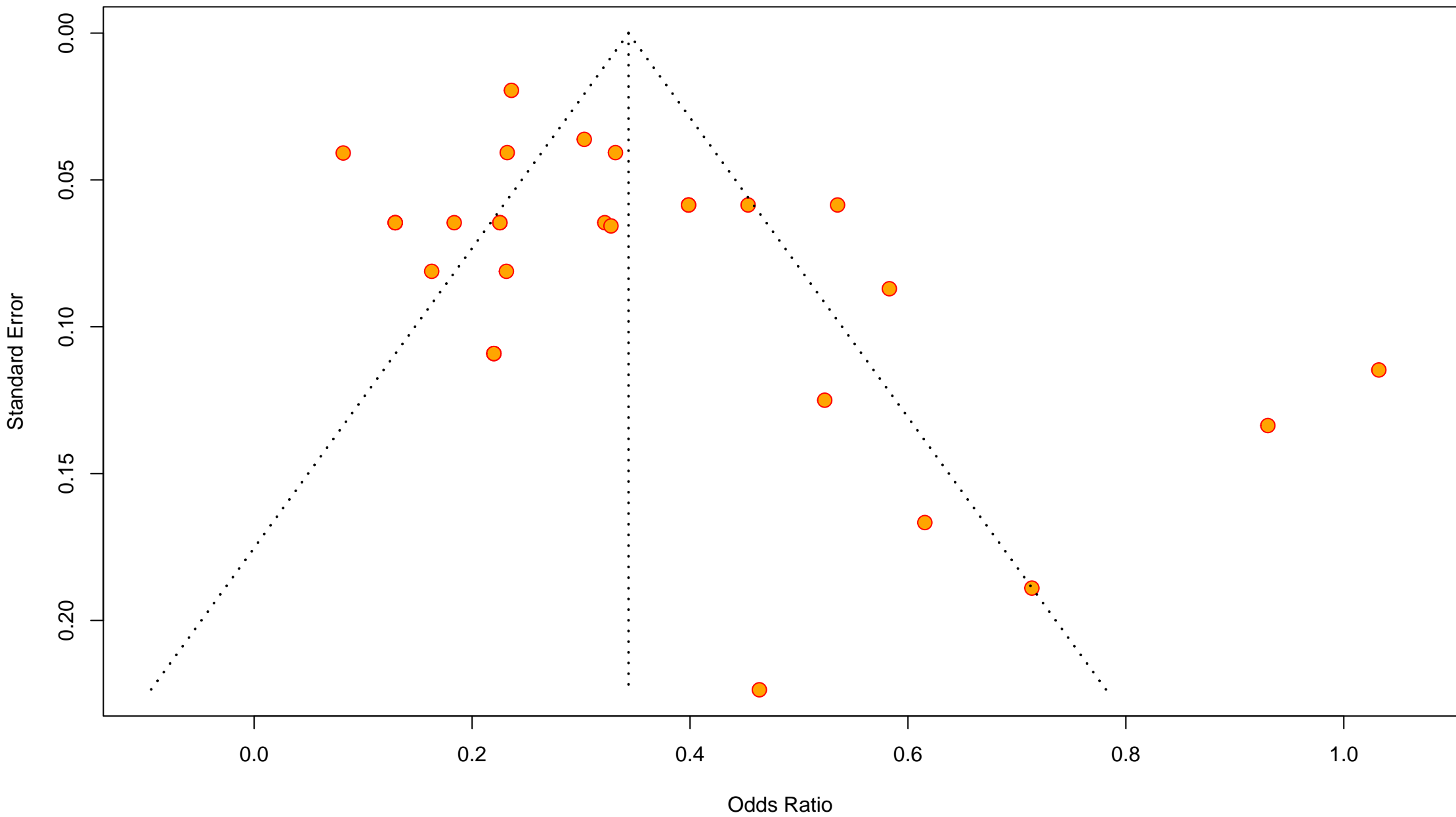

Supplement: FigureS12.pdf [file TEMI_A_2651466_SM5568.pdf]

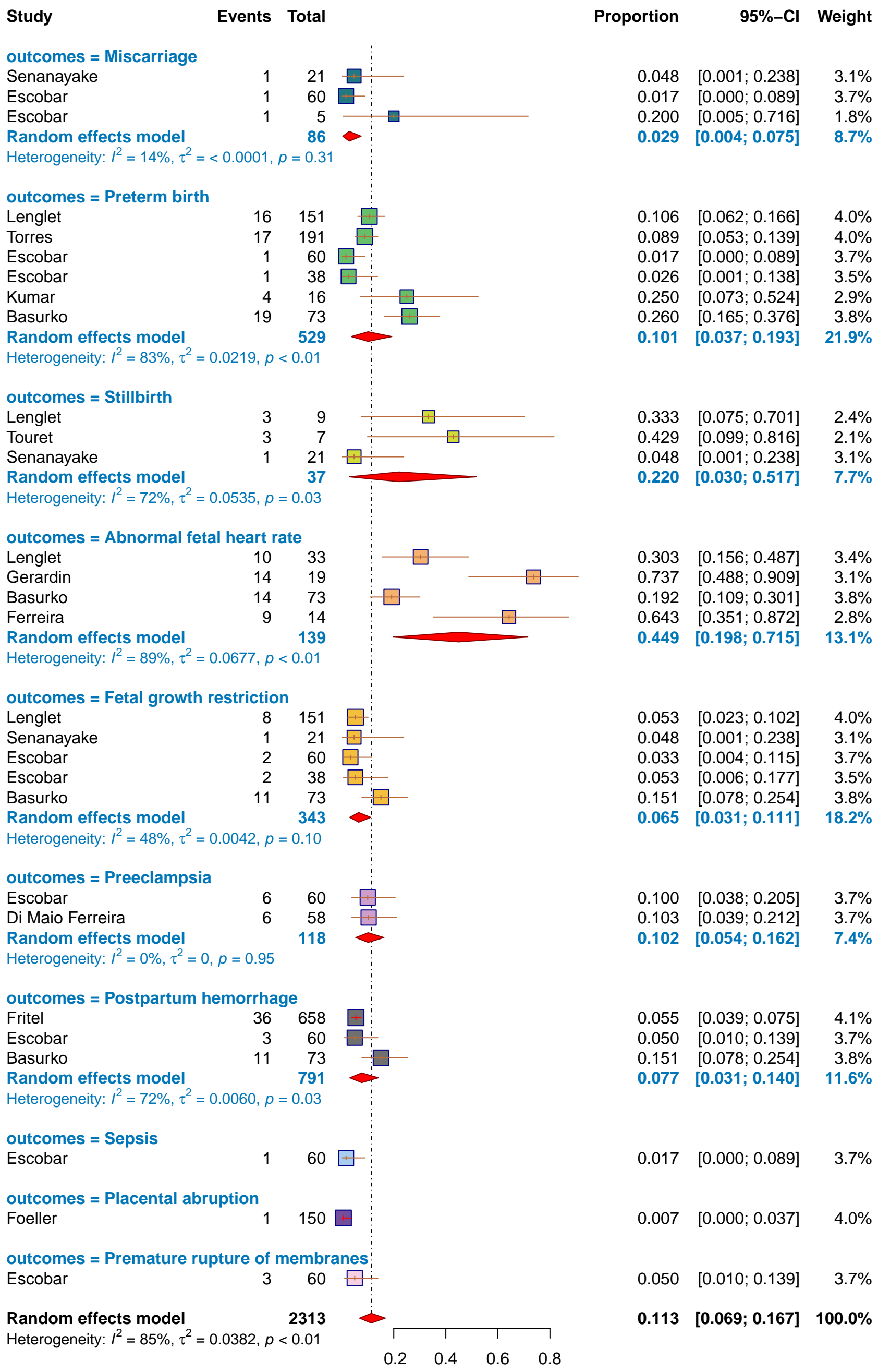

Supplement: FigureS2.pdf [file TEMI_A_2651466_SM5567.pdf]

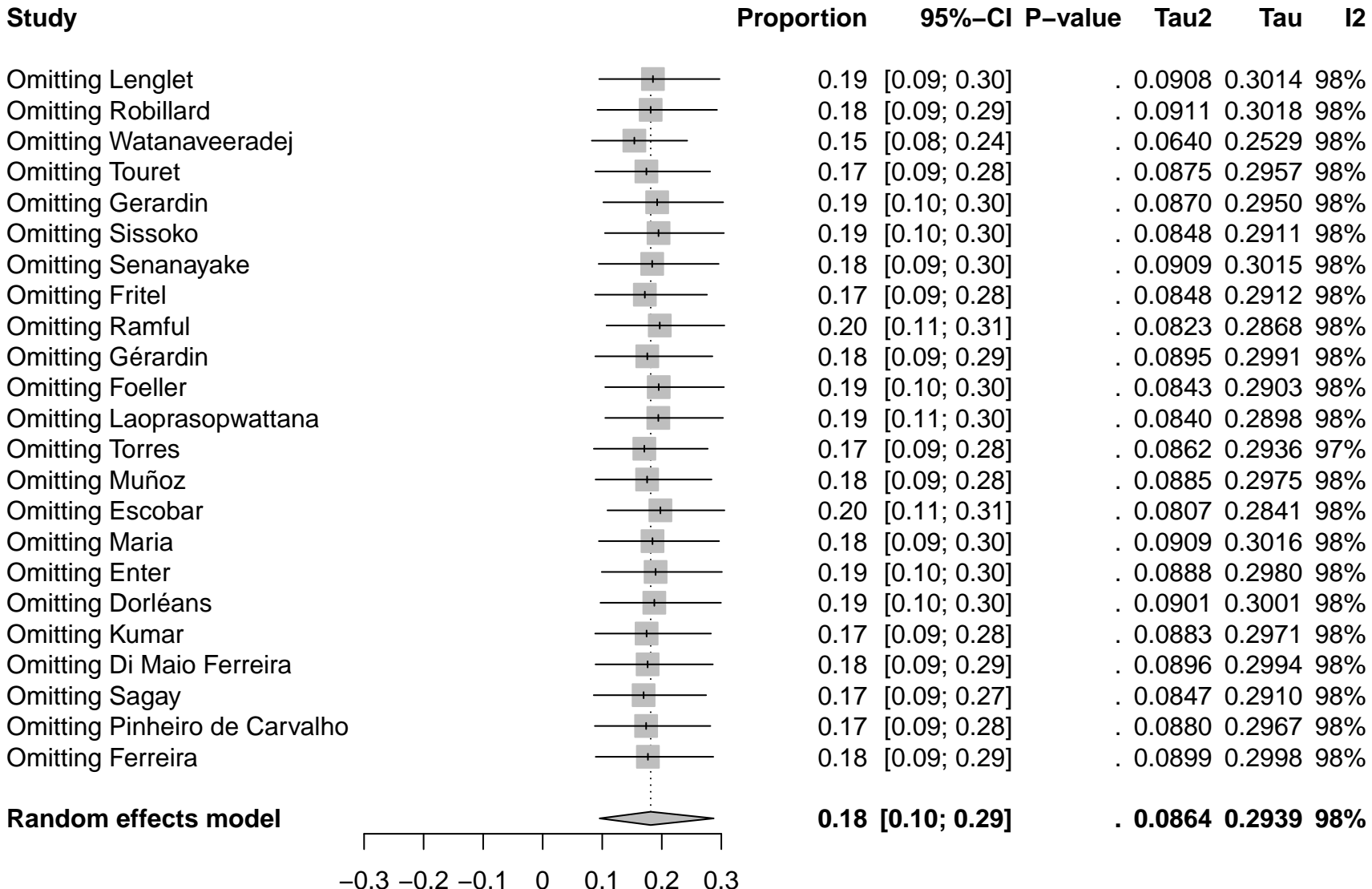

Supplement: FigureS7.pdf [file TEMI_A_2651466_SM5566.pdf]

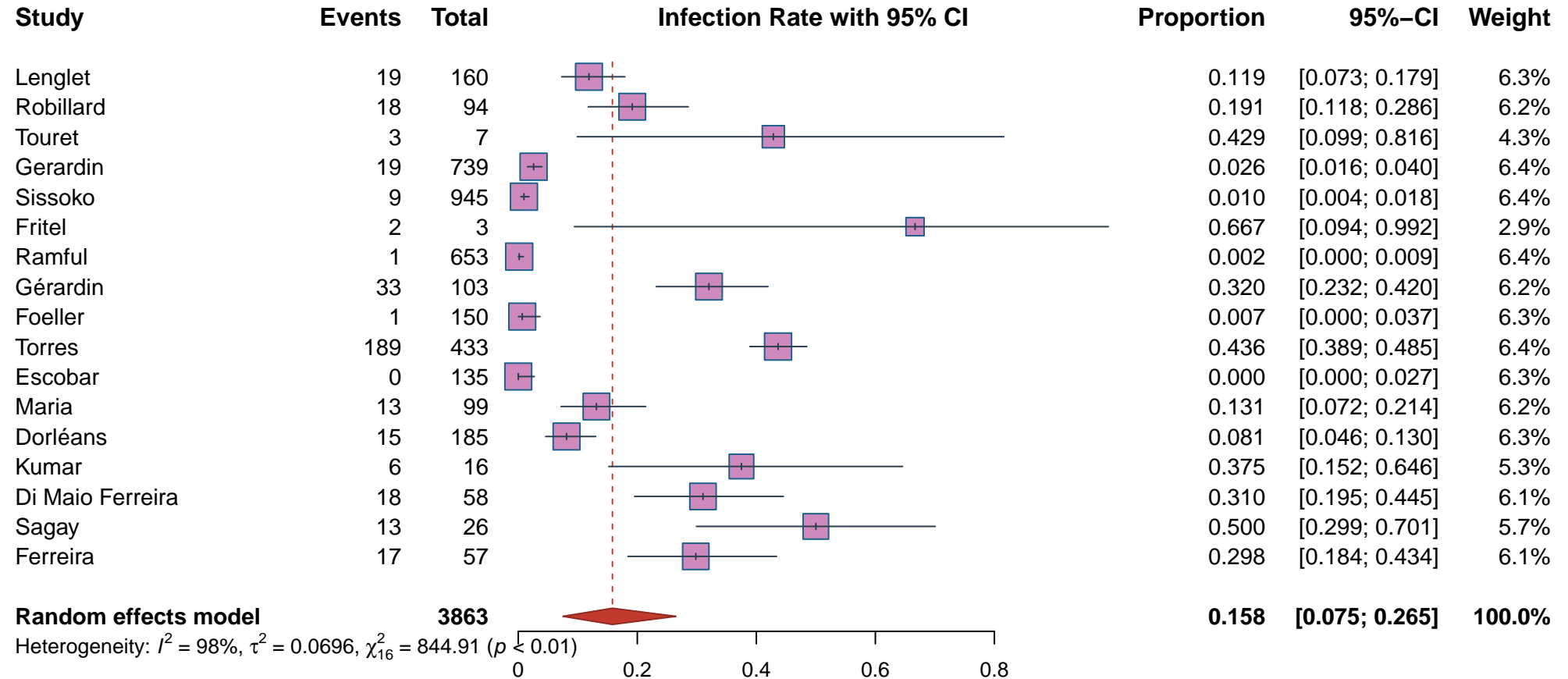

Supplement: FigureS9.pdf [file TEMI_A_2651466_SM5565.pdf]

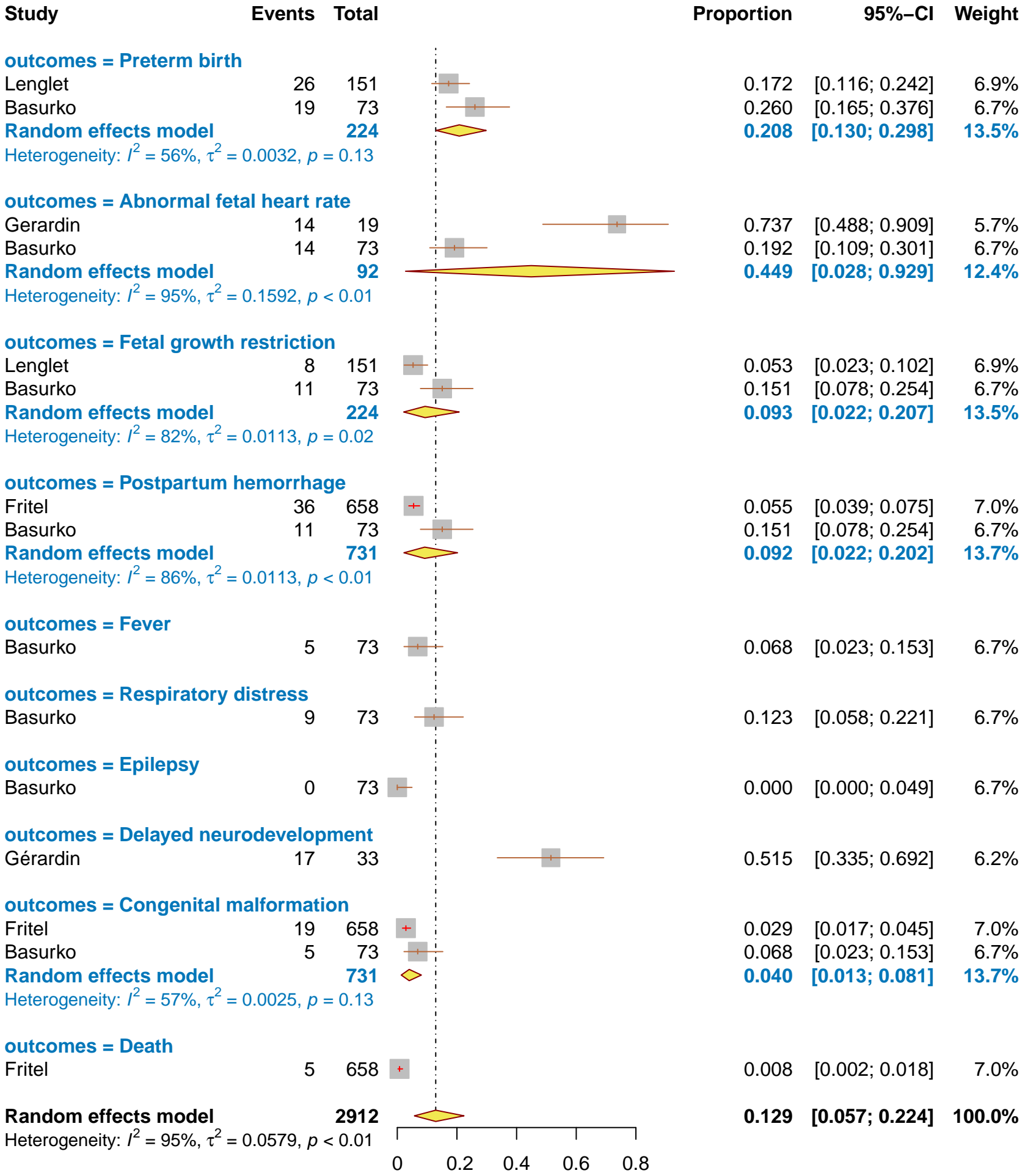

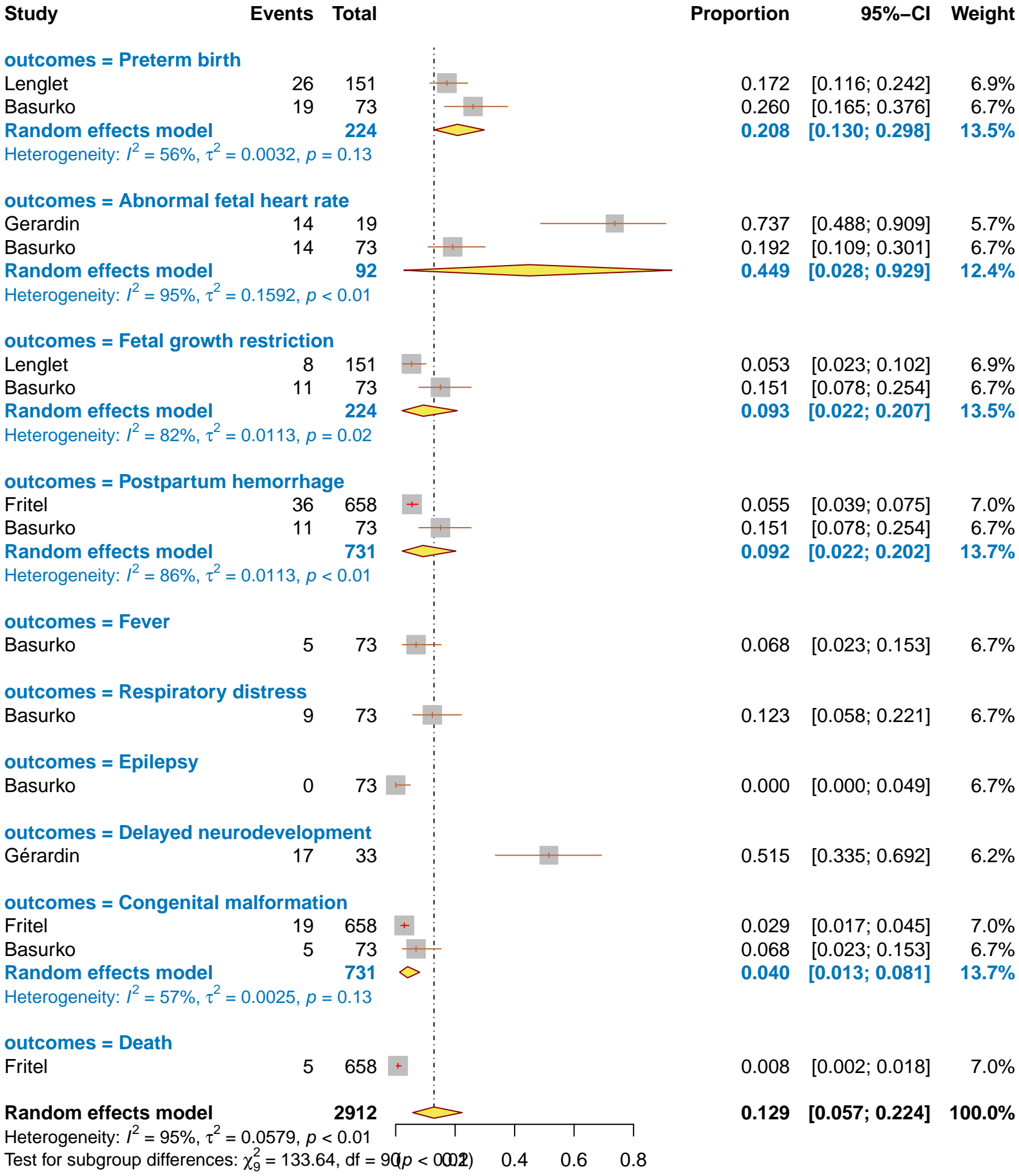

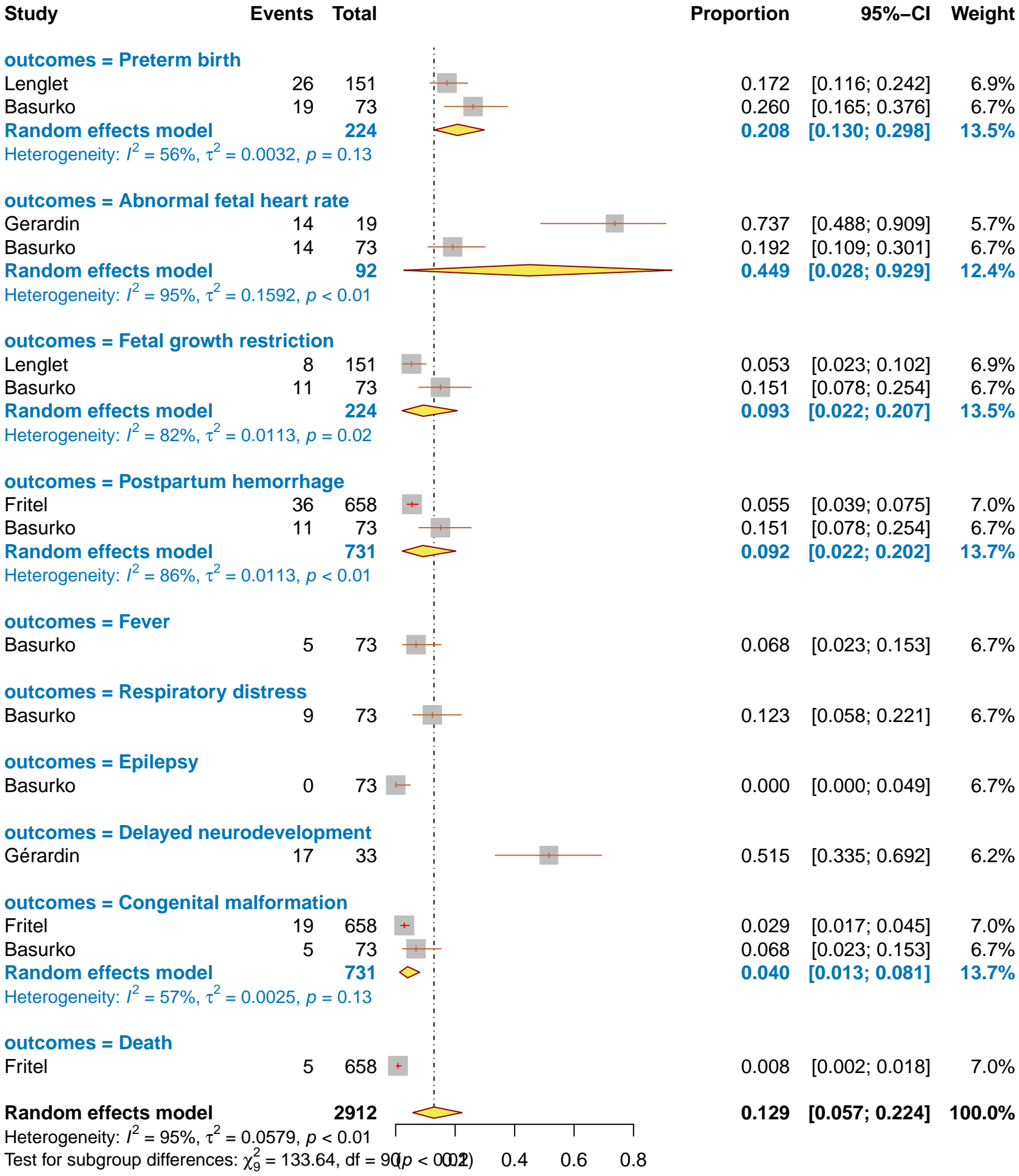

Supplement: FigureS4.pdf [file TEMI_A_2651466_SM5563.pdf]

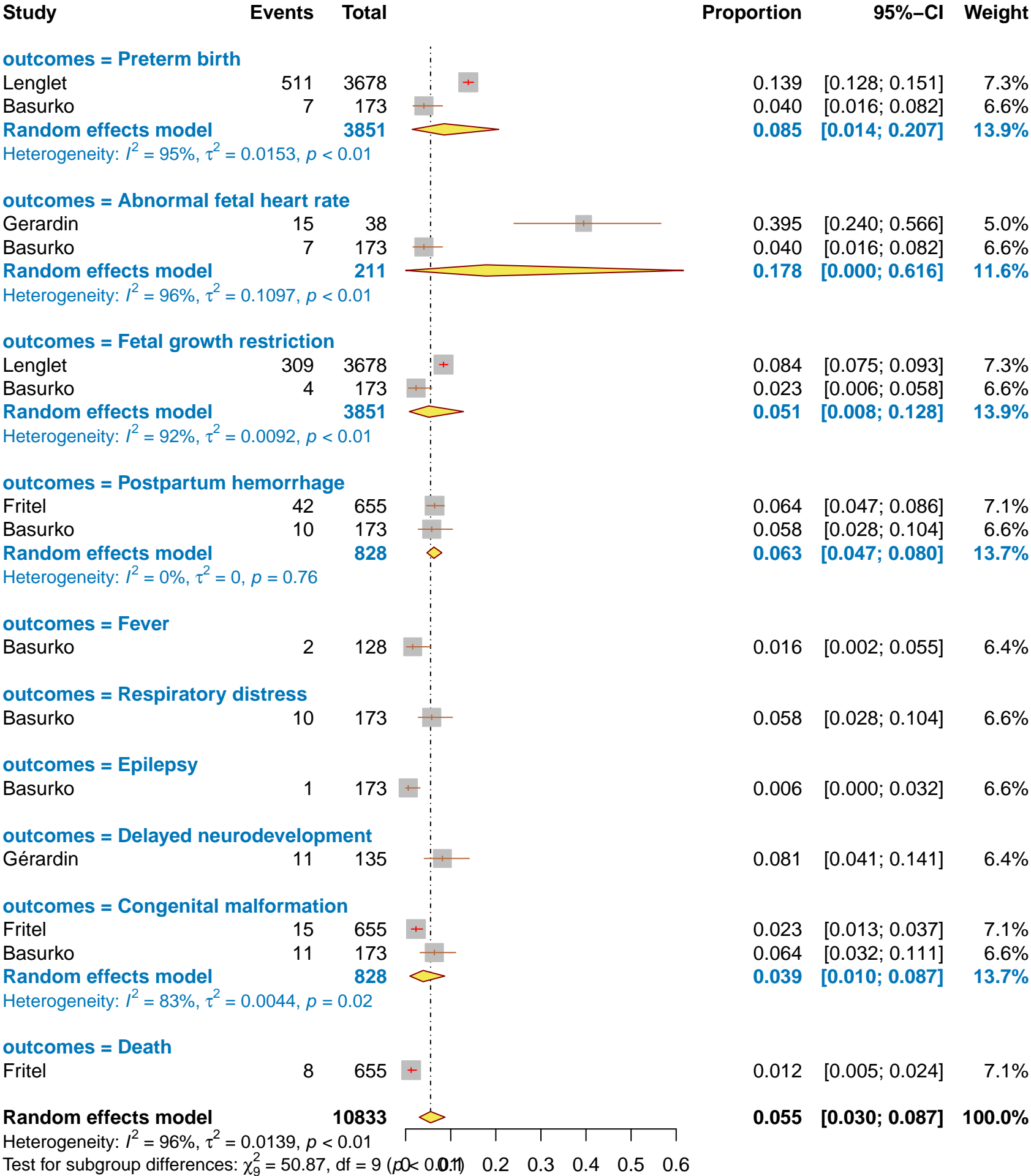

Supplement: FigureS5.pdf [file TEMI_A_2651466_SM5561.pdf]

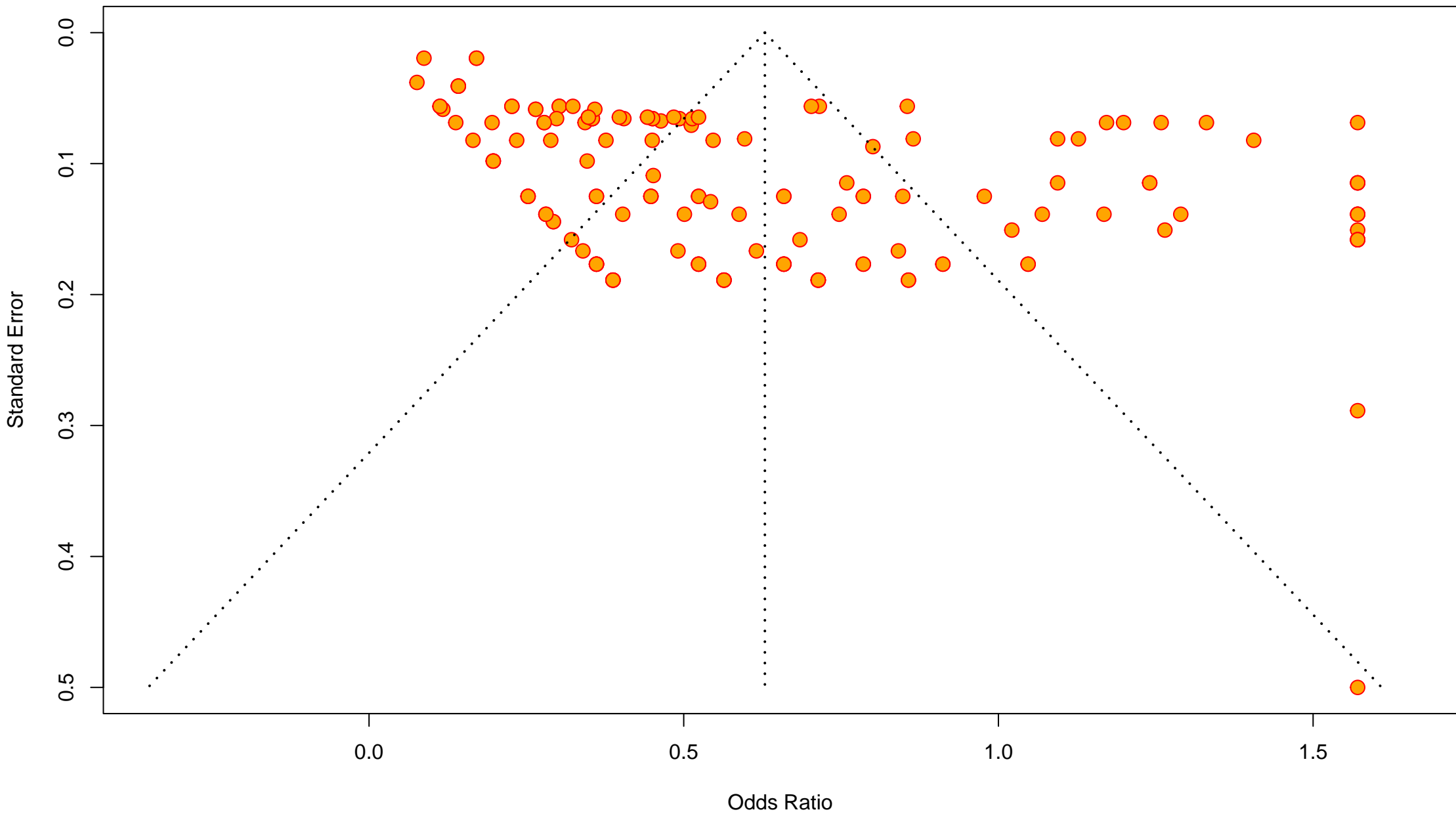

Supplement: FigureS14.pdf [file TEMI_A_2651466_SM5560.pdf]

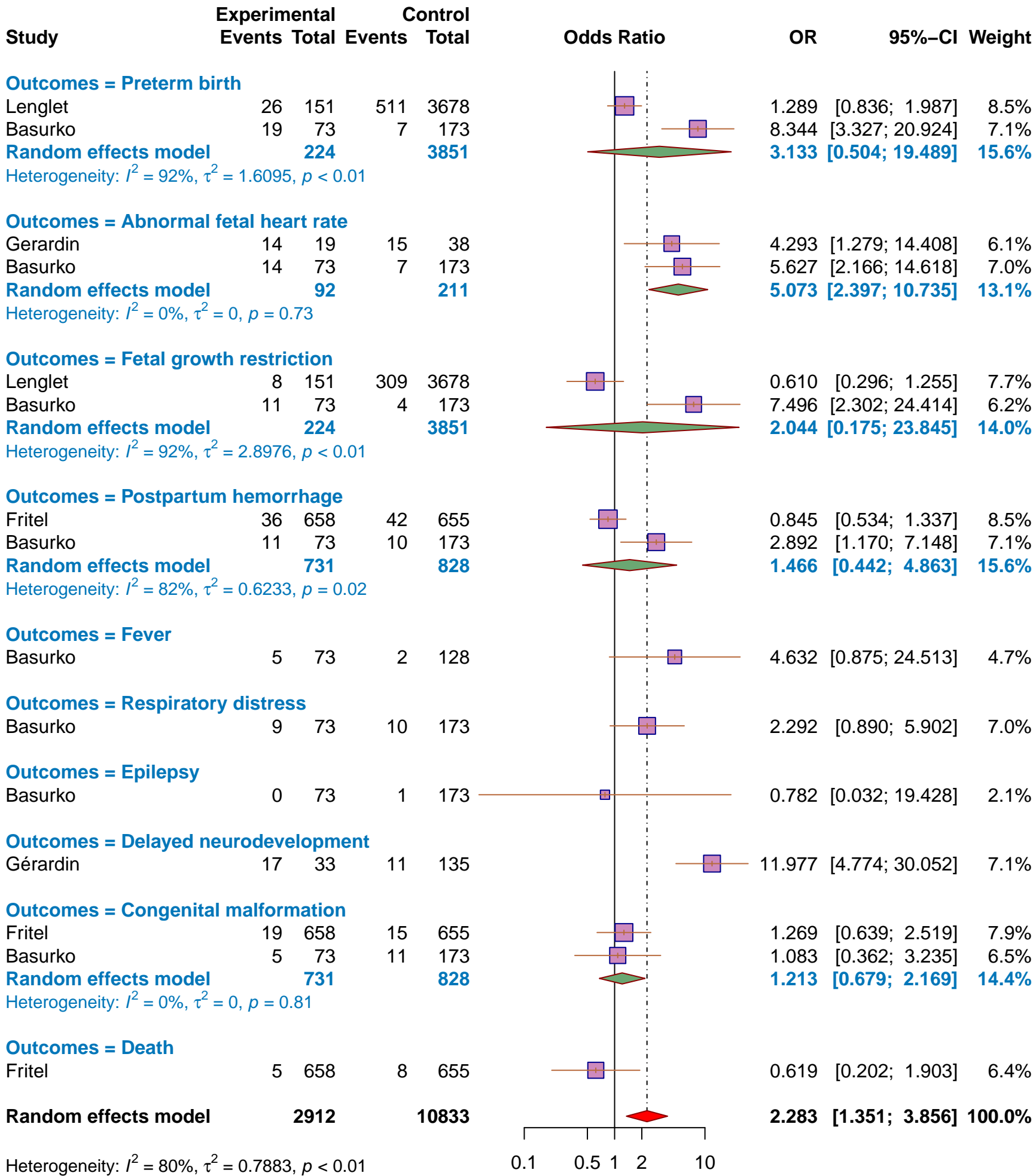

Supplement: FigureS6.pdf [file TEMI_A_2651466_SM5559.pdf]
