# Supplementary material for: Maternal Chikungunya virus infection and pregnancy outcomes: a global systematic review and meta-analysis of vertical transmission dynamics and associated morbidity
Source: Emerg Microbes Infect. 2026 Mar 25;15(1):2651466. doi: 10.1080/22221751.2026.2651466 (PMC13101005; doi:10.1080/22221751.2026.2651466)
Supplement: Appendix.pdf [file TEMI_A_2651466_SM5564.pdf]

Supplementary web appendix for:

Pregnancy and Neonatal Outcomes Following Chikungunya Virus Infection During Pregnancy: A Global Systematic Review and Meta-Analysis

Figure S1. Forest plot of CHIKV vertical transmission rates among pregnant women across different regions. 1
Figure S2. Forest plot of positive rates for different pregnancy outcomes in pregnant women infected with CHIKV. 2
Figure S3. Forest plot of positive rates for different neonatal outcomes in pregnant women infected with CHIKV. 3
Figure S4. Incidence of adverse pregnancy outcomes and neonatal outcomes in the CHIKV positive group of pregnant women in six comparative cohort studies. 4
Figure S5. Incidence of adverse pregnancy outcomes and neonatal outcomes in the CHIKV negative group of pregnant women in six comparative cohort studies. 5
Figure S6. Forest plot of adverse pregnancy and neonatal outcomes rates between pregnant women infected and uninfected with CHIKV. 6
Figure S7. Sensitivity analysis of vertical transmission rates among CHIKV-infected pregnant women. 7
Figure S8. Publication bias analysis of vertical transmission rates among CHIKV-infected pregnant women. 7
Figure S9. Sensitivity analysis restricted to studies with molecular confirmation of neonatal CHIKV infection (RT-PCR alone or combined with serology). 8
Figure S10. Leave-one-out sensitivity analysis of pooled vertical transmission estimates.8
Figure S11. Leave-one-out sensitivity analysis of the positive rates of adverse pregnancy outcomes among CHIKV-infected pregnant women. 9
Figure S12. Publication bias analysis of the positive rates of adverse pregnancy outcomes among CHIKV-infected pregnant women. 9
Figure S13. Leave-one-out sensitivity analysis of the positive rates of adverse neonatal outcomes following maternal CHIKV infection. 10

Figure S14. Publication bias analysis of the positive rates of adverse neonatal outcomes among CHIKV-infected pregnant women. 11
Figure S15. Leave-one-out sensitivity analysis comparing adverse pregnancy and neonatal outcomes in CHIKV-infected and uninfected pregnant women. 12
Figure S16. Publication bias analysis comparing adverse pregnancy and neonatal outcomes in CHIKV-infected and uninfected pregnant women. 12
Table S1. Search strategy. 13
Supplementary Methods 14
Table S2. Characteristics of included studies. 16
Table S3. The quality evaluation of all studies. 23
Table S4. Vertical transmission rates of CHIKV among pregnant women infected at different gestational stages. 24
Table S5. Vertical transmission rates of CHIKV among pregnant women across different regions. 24
Table S6. Adverse pregnancy outcomes following maternal CHIKV infection (overall and by outcome). 25
Table S7. Adverse neonatal outcomes following maternal CHIKV infection (overall and by outcome). 26
Table S8. Comparison of adverse pregnancy and neonatal outcomes between pregnant women infected and uninfected with CHIKV. 27
Table S9. Publication bias analysis. 29
Table S10. Outcome definition mapping table. 30
References 33

Figure S1. Forest plot of CHIKV vertical transmission rates among pregnant women across different regions.

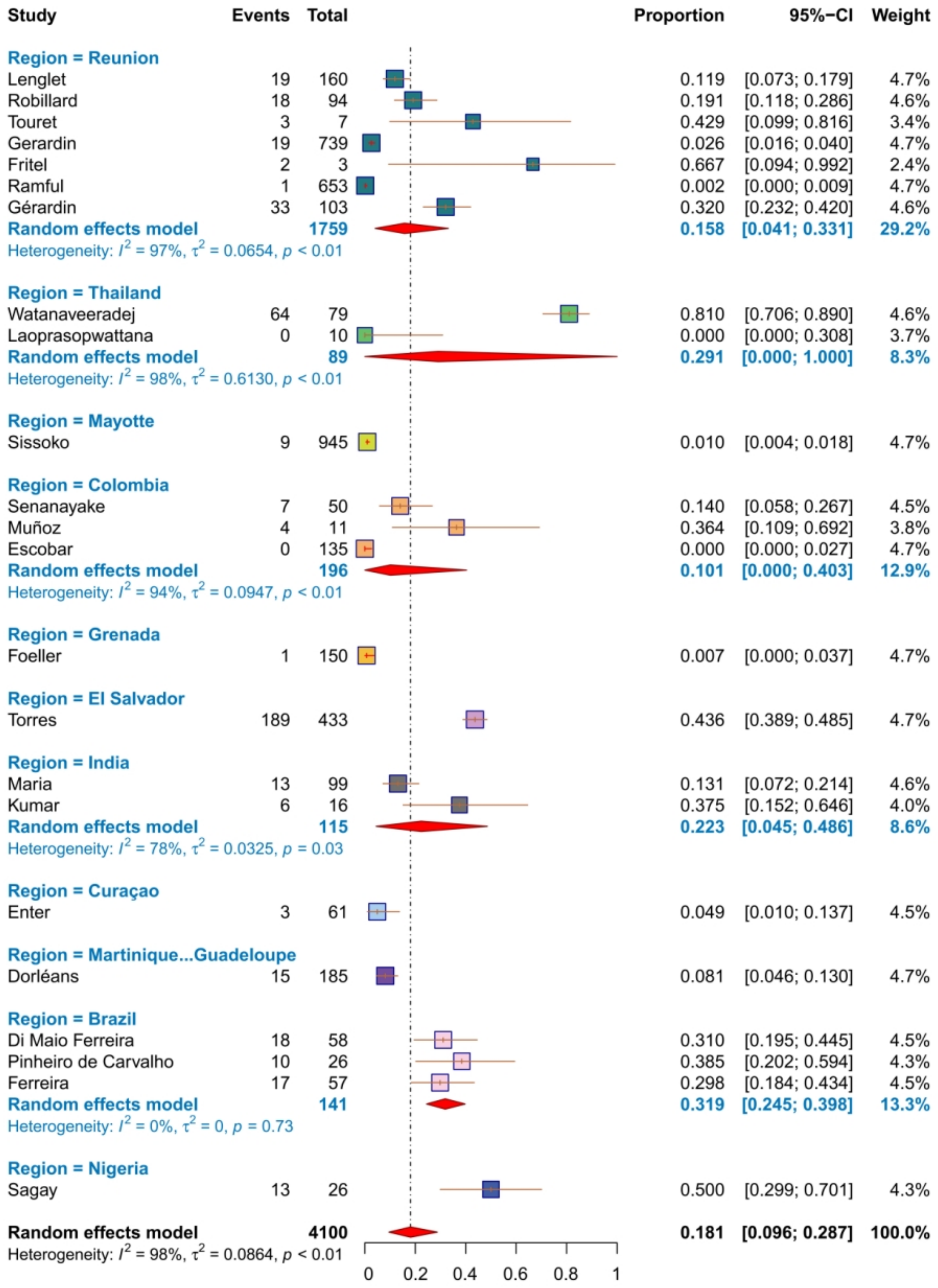

Figure S2. Forest plot of positive rates for different pregnancy outcomes in pregnant women infected with CHIKV.

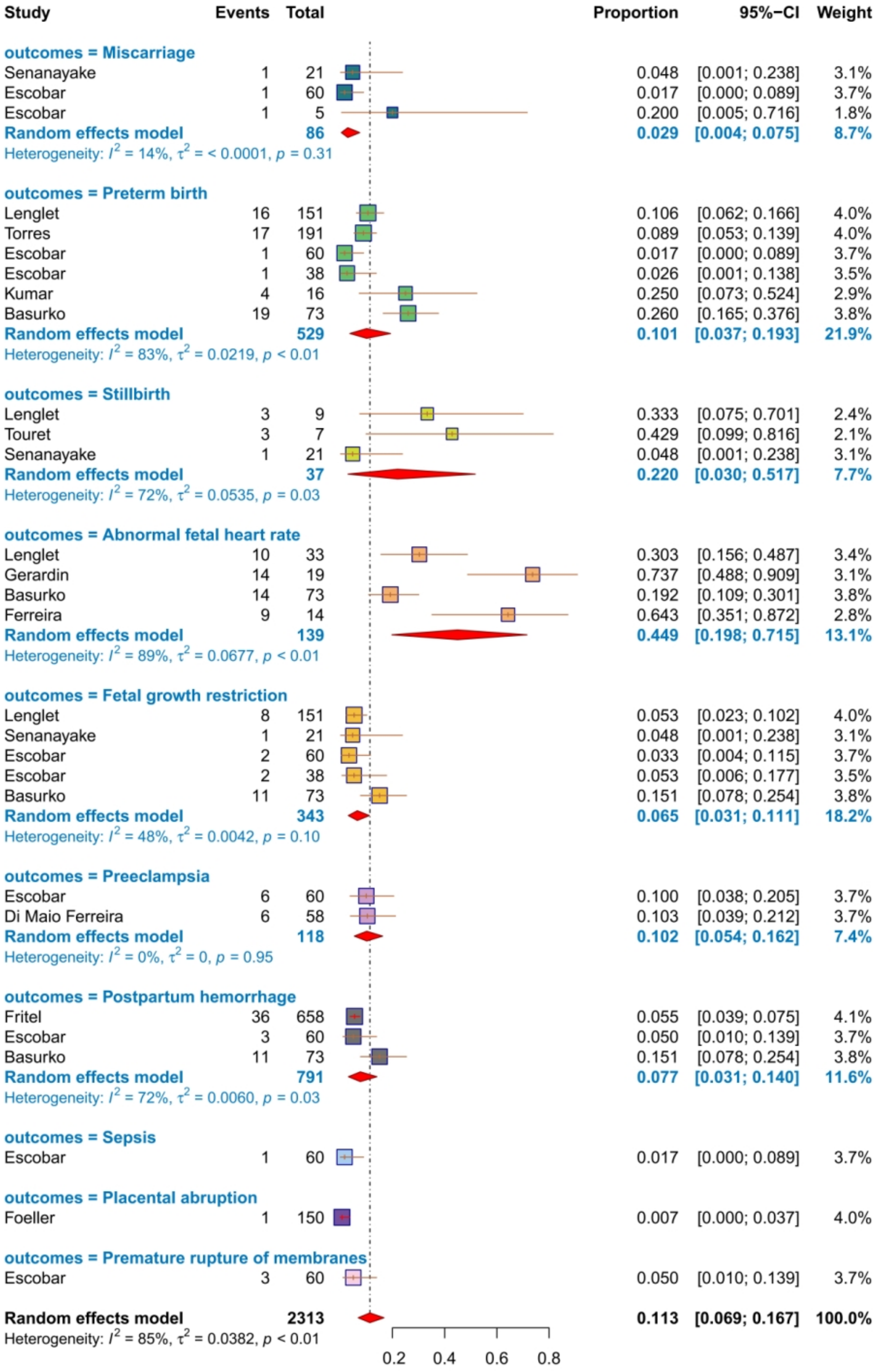

Figure S3. Forest plot of positive rates for different neonatal outcomes in pregnant women infected with CHIKV.

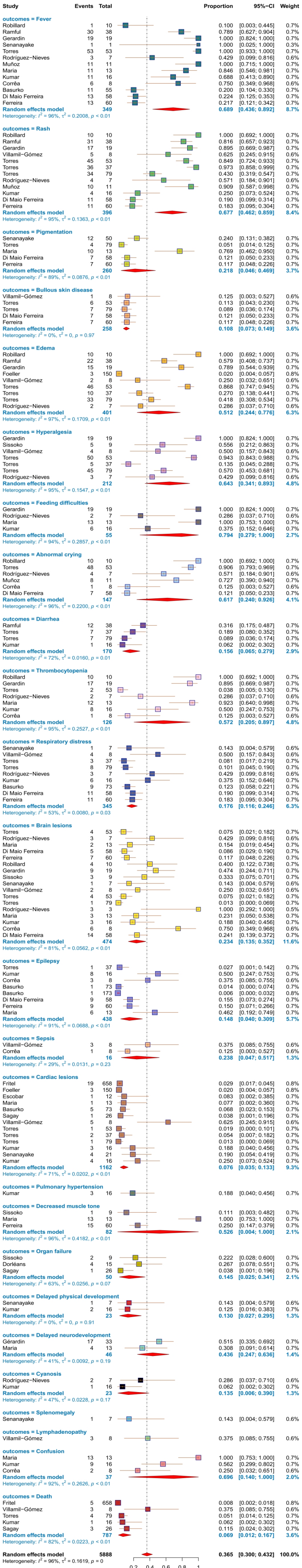

Figure S4. Incidence of adverse pregnancy outcomes and neonatal outcomes in the CHIKV positive group of pregnant women in six comparative cohort studies.

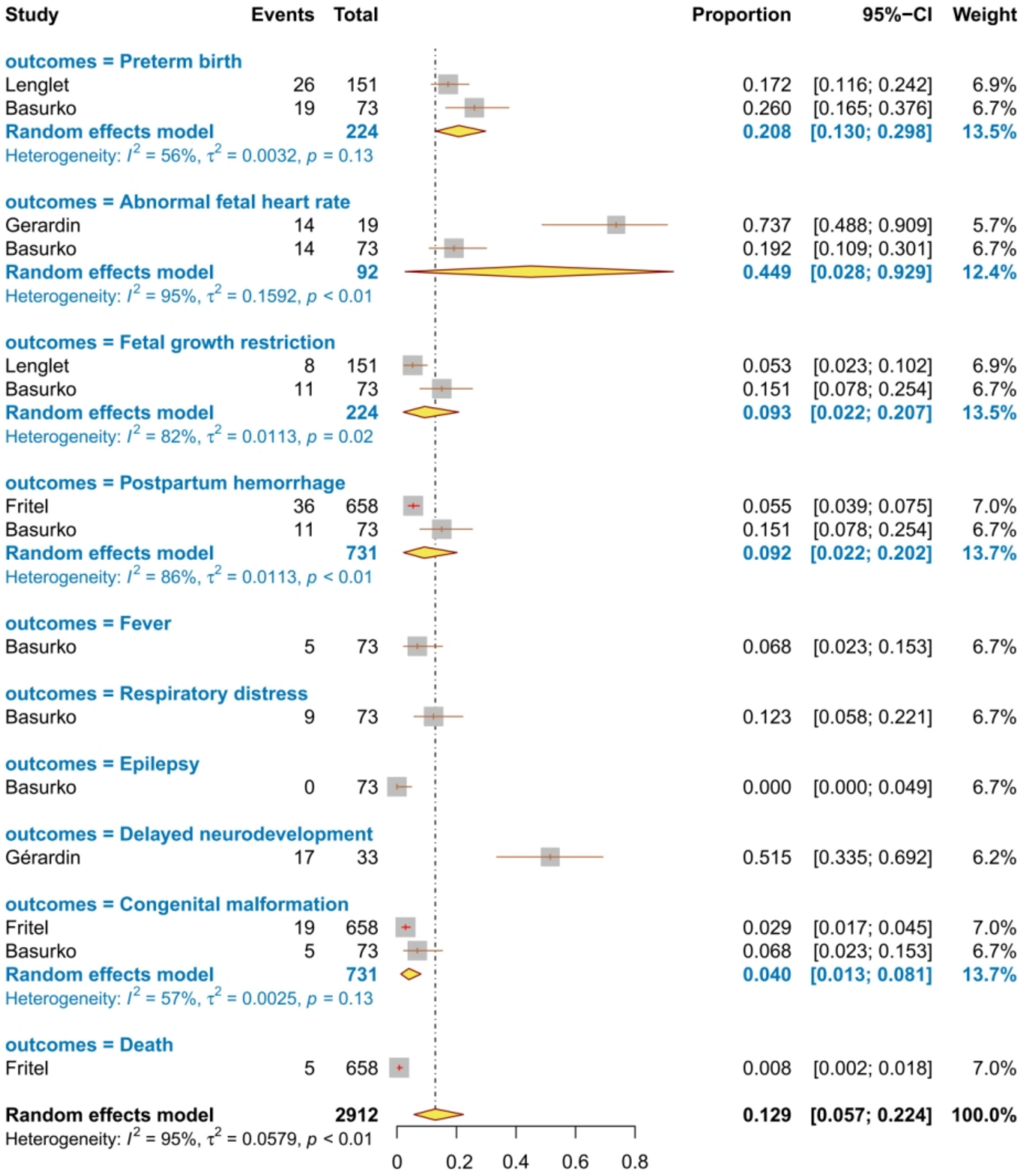

Figure S5. Incidence of adverse pregnancy outcomes and neonatal outcomes in the CHIKV negative group of pregnant women in six comparative cohort studies.

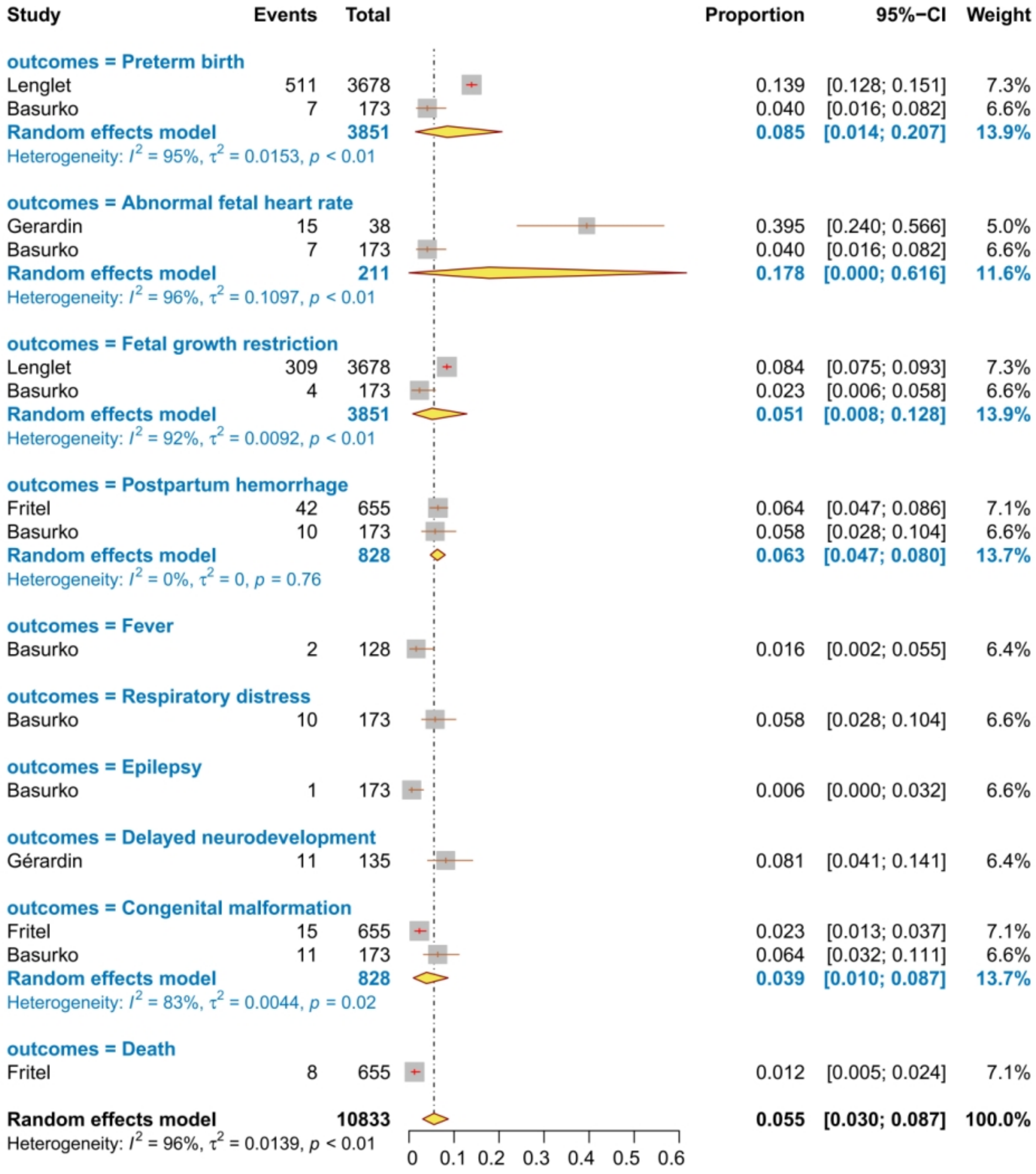

Figure S6. Forest plot of adverse pregnancy and neonatal outcomes rates between pregnant women infected and uninfected with CHIKV.

Study	Experimental Events	Experimental Total	Control Events	Control Total	OR	95%−CI	Weight
**Outcomes = Preterm birth**							
Lenglet	26	151	511	3678	1.289	[0.836; 1.987]	8.5%
Basurko	19	73	7	173	8.344	[3.327; 20.924]	7.1%
**Random effects model**		**224**		**3851**	**3.133**	**[0.504; 19.489]**	**15.6%**
Heterogeneity: $I^2 = 92\%$, $\tau^2 = 1.6095$, $p < 0.01$							
**Outcomes = Abnormal fetal heart rate**							
Gerardin	14	19	15	38	4.293	[1.279; 14.408]	6.1%
Basurko	14	73	7	173	5.627	[2.166; 14.618]	7.0%
**Random effects model**		**92**		**211**	**5.073**	**[2.397; 10.735]**	**13.1%**
Heterogeneity: $I^2 = 0\%$, $\tau^2 = 0$, $p = 0.73$							
**Outcomes = Fetal growth restriction**							
Lenglet	8	151	309	3678	0.610	[0.296; 1.255]	7.7%
Basurko	11	73	4	173	7.496	[2.302; 24.414]	6.2%
**Random effects model**		**224**		**3851**	**2.044**	**[0.175; 23.845]**	**14.0%**
Heterogeneity: $I^2 = 92\%$, $\tau^2 = 2.8976$, $p < 0.01$							
**Outcomes = Postpartum hemorrhage**							
Fritel	36	658	42	655	0.845	[0.534; 1.337]	8.5%
Basurko	11	73	10	173	2.892	[1.170; 7.148]	7.1%
**Random effects model**		**731**		**828**	**1.466**	**[0.442; 4.863]**	**15.6%**
Heterogeneity: $I^2 = 82\%$, $\tau^2 = 0.6233$, $p = 0.02$							
**Outcomes = Fever**							
Basurko	5	73	2	128	4.632	[0.875; 24.513]	4.7%
**Outcomes = Respiratory distress**							
Basurko	9	73	10	173	2.292	[0.890; 5.902]	7.0%
**Outcomes = Epilepsy**							
Basurko	0	73	1	173	0.782	[0.032; 19.428]	2.1%
**Outcomes = Delayed neurodevelopment**							
Gérardin	17	33	11	135	11.977	[4.774; 30.052]	7.1%
**Outcomes = Congenital malformation**							
Fritel	19	658	15	655	1.269	[0.639; 2.519]	7.9%
Basurko	5	73	11	173	1.083	[0.362; 3.235]	6.5%
**Random effects model**		**731**		**828**	**1.213**	**[0.679; 2.169]**	**14.4%**
Heterogeneity: $I^2 = 0\%$, $\tau^2 = 0$, $p = 0.81$							
**Outcomes = Death**							
Fritel	5	658	8	655	0.619	[0.202; 1.903]	6.4%
**Random effects model**		**2912**		**10833**	**2.283**	**[1.351; 3.856]**	**100.0%**
Heterogeneity: $I^2 = 80\%$, $\tau^2 = 0.7883$, $p < 0.01$							

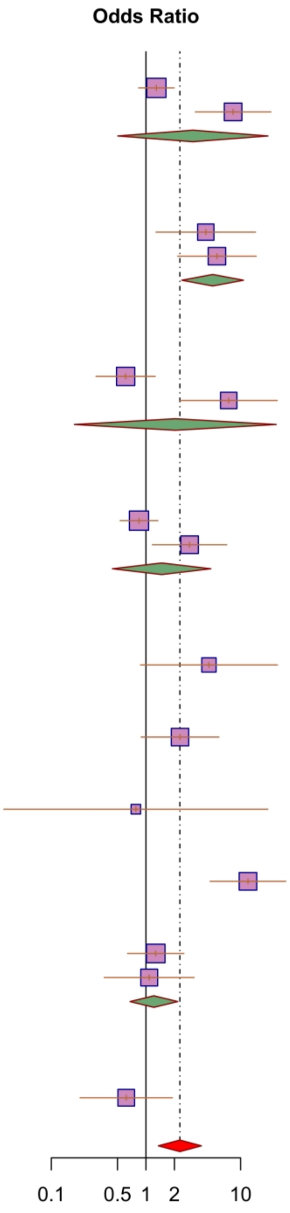

Figure S7. Sensitivity analysis of vertical transmission rates among CHIKV-infected pregnant women.

Study	Proportion	95%−CI	P−value	Tau2	Tau	I2
Omitting Lenglet	0.19	[0.09; 0.30]	.	0.0908	0.3014	98%
Omitting Robillard	0.18	[0.09; 0.29]	.	0.0911	0.3018	98%
Omitting Watanaveeradej	0.15	[0.08; 0.24]	.	0.0640	0.2529	98%
Omitting Touret	0.17	[0.09; 0.28]	.	0.0875	0.2957	98%
Omitting Gerardin	0.19	[0.10; 0.30]	.	0.0870	0.2950	98%
Omitting Sissoko	0.19	[0.10; 0.30]	.	0.0848	0.2911	98%
Omitting Senanayake	0.18	[0.09; 0.30]	.	0.0909	0.3015	98%
Omitting Fritel	0.17	[0.09; 0.28]	.	0.0848	0.2912	98%
Omitting Ramful	0.20	[0.11; 0.31]	.	0.0823	0.2868	98%
Omitting Gérardin	0.18	[0.09; 0.29]	.	0.0895	0.2991	98%
Omitting Foeller	0.19	[0.10; 0.30]	.	0.0843	0.2903	98%
Omitting Laoprasopwattana	0.19	[0.11; 0.30]	.	0.0840	0.2898	98%
Omitting Torres	0.17	[0.09; 0.28]	.	0.0862	0.2936	97%
Omitting Muñoz	0.18	[0.09; 0.28]	.	0.0885	0.2975	98%
Omitting Escobar	0.20	[0.11; 0.31]	.	0.0807	0.2841	98%
Omitting Maria	0.18	[0.09; 0.30]	.	0.0909	0.3016	98%
Omitting Enter	0.19	[0.10; 0.30]	.	0.0888	0.2980	98%
Omitting Dorléans	0.19	[0.10; 0.30]	.	0.0901	0.3001	98%
Omitting Kumar	0.17	[0.09; 0.28]	.	0.0883	0.2971	98%
Omitting Di Maio Ferreira	0.18	[0.09; 0.29]	.	0.0896	0.2994	98%
Omitting Sagay	0.17	[0.09; 0.27]	.	0.0847	0.2910	98%
Omitting Pinheiro de Carvalho	0.17	[0.09; 0.28]	.	0.0880	0.2967	98%
Omitting Ferreira	0.18	[0.09; 0.29]	.	0.0899	0.2998	98%
**Random effects model**	**0.18**	**[0.10; 0.29]**	**.**	**0.0864**	**0.2939**	**98%**

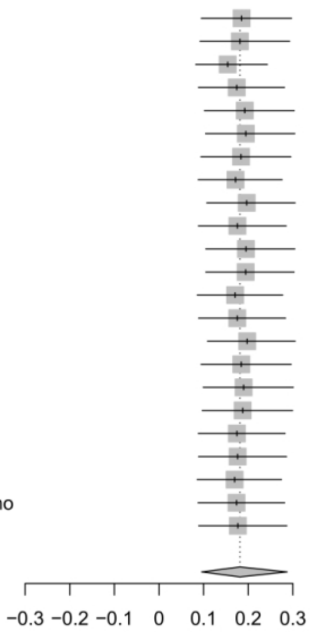

Figure S8. Publication bias analysis of vertical transmission rates among CHIKV-infected pregnant women.

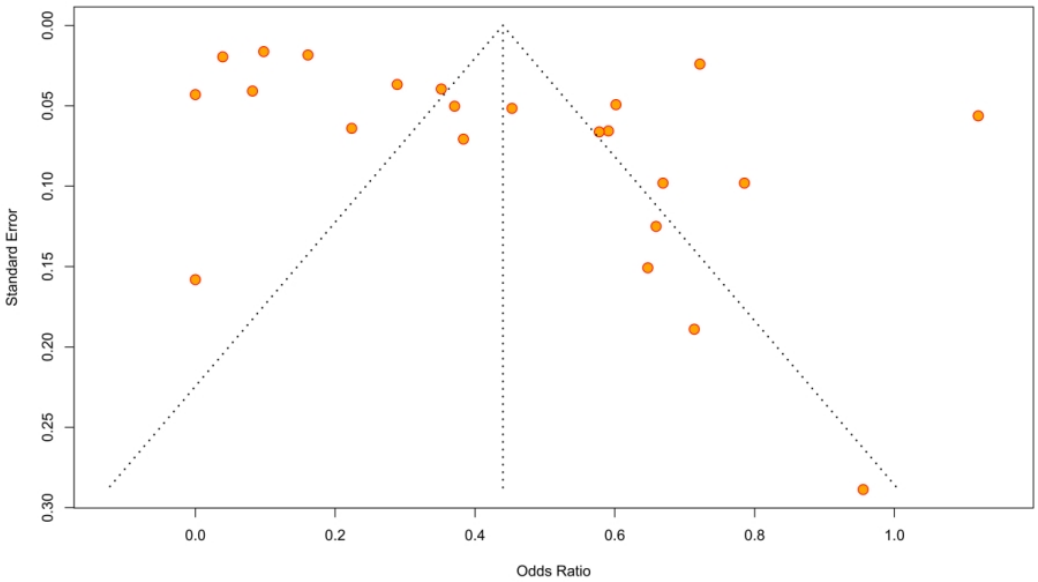

Figure S9. Sensitivity analysis restricted to studies with molecular confirmation of neonatal CHIKV infection (RT-PCR alone or combined with serology).

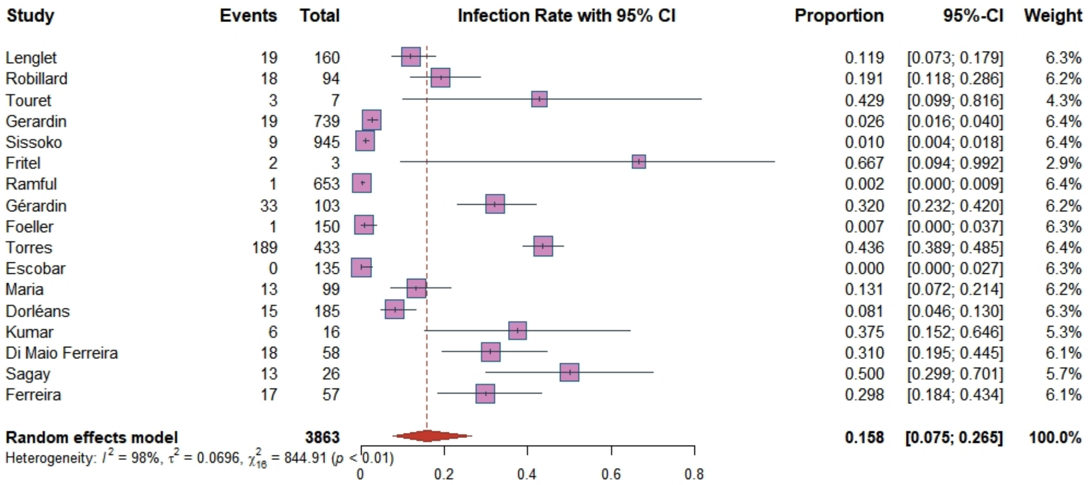

Figure S10. Leave-one-out sensitivity analysis of pooled vertical transmission estimates.

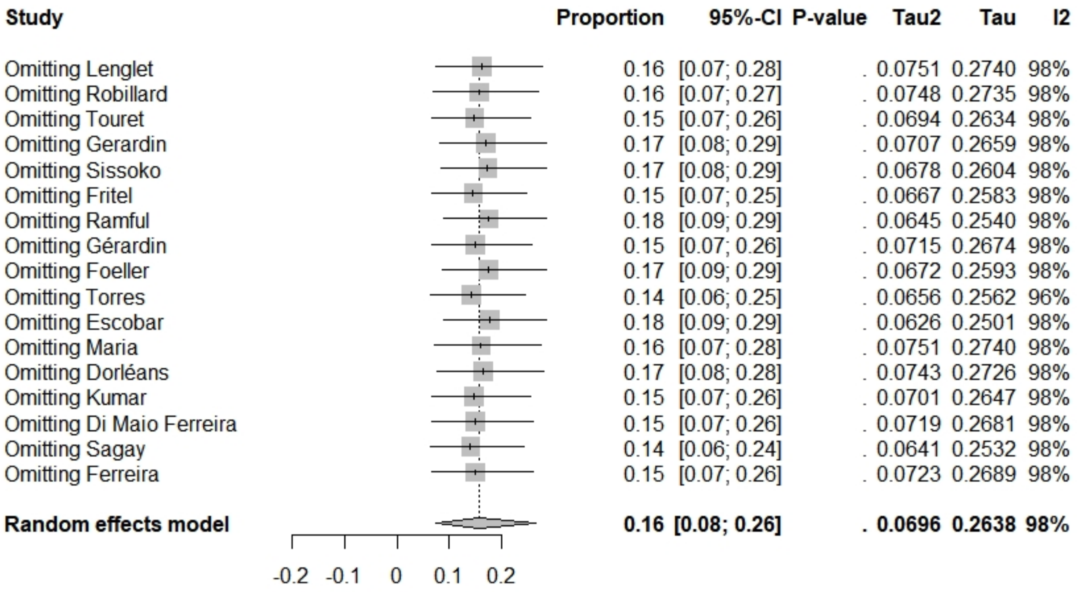

Figure S11. Leave-one-out sensitivity analysis of the positive rates of adverse pregnancy outcomes among CHIKV-infected pregnant women.

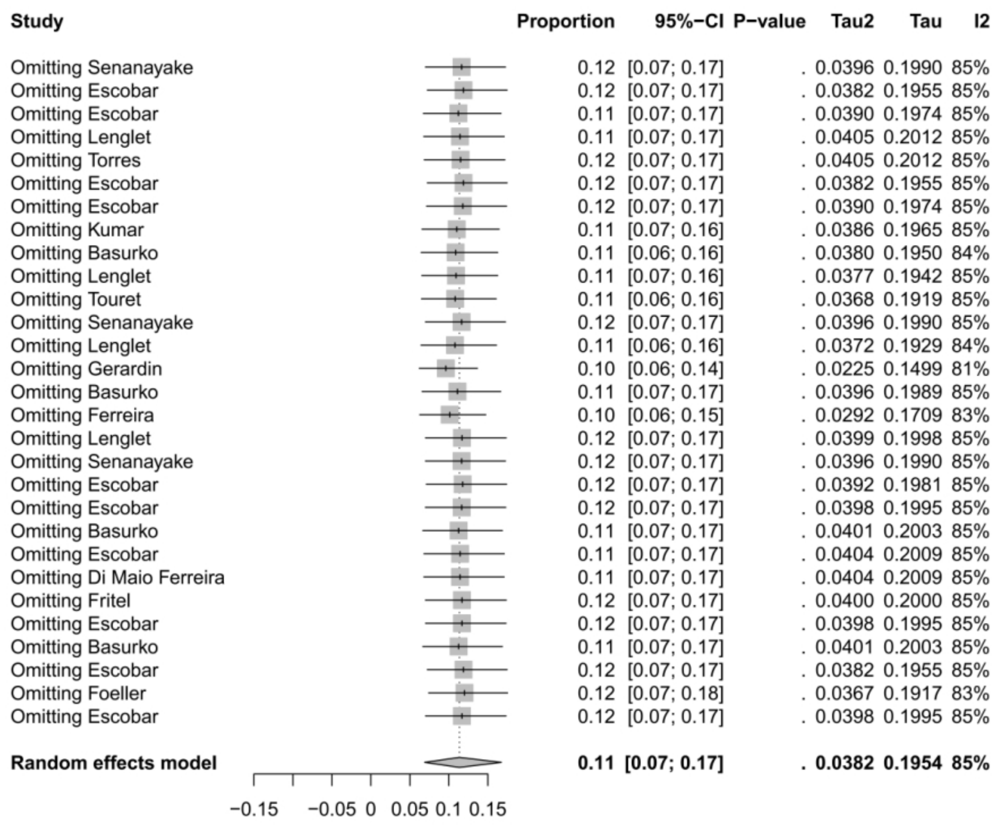

Figure S12. Publication bias analysis of the positive rates of adverse pregnancy outcomes among CHIKV-infected pregnant women.

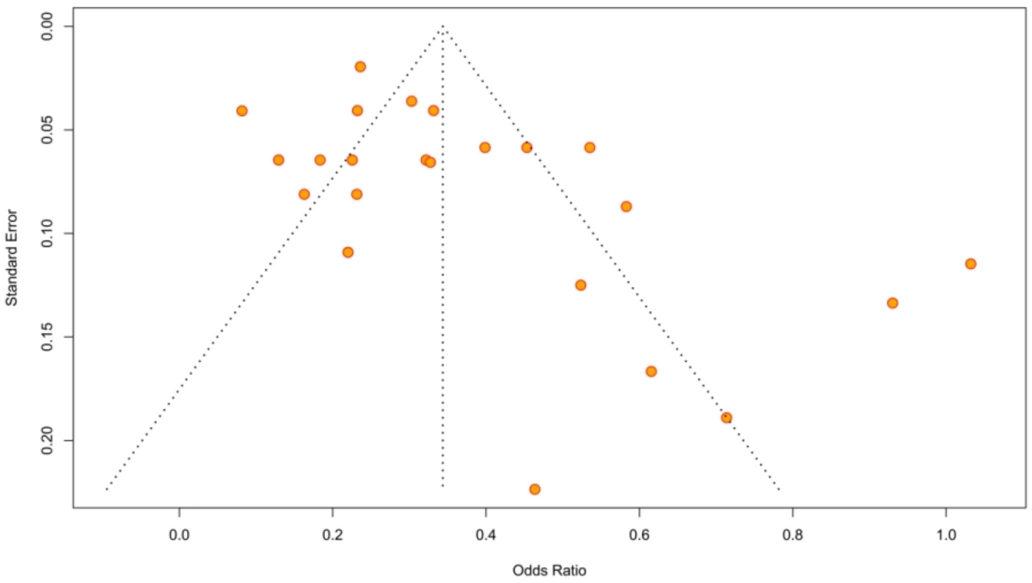

Figure S13. Leave-one-out sensitivity analysis of the positive rates of adverse neonatal outcomes following maternal CHIKV infection.

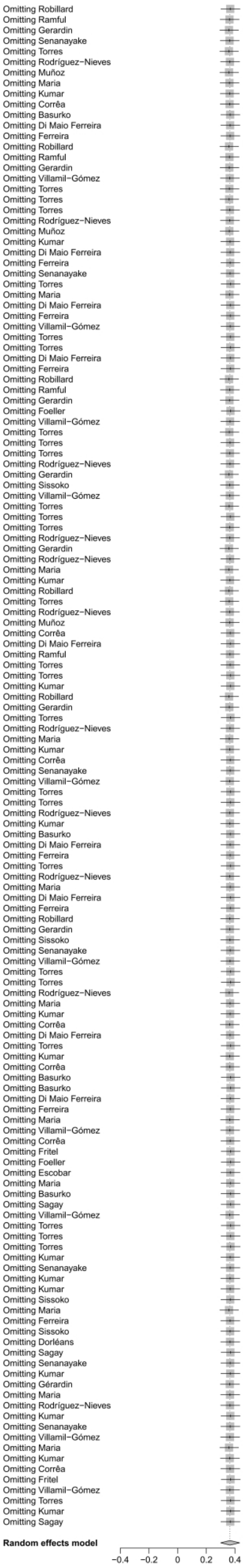

Study	Proportion	95%−CI	P−value	Tau2	Tau	I2
Omitting Robillard	0.37	[0.30; 0.43]	.	0.1624	0.4030	96%
Omitting Ramful	0.36	[0.30; 0.43]	.	0.1616	0.4019	96%
Omitting Gerardin	0.36	[0.29; 0.42]	.	0.1568	0.3960	96%
Omitting Senanayake	0.36	[0.30; 0.43]	.	0.1613	0.4016	96%
Omitting Torres	0.36	[0.29; 0.42]	.	0.1562	0.3953	95%
Omitting Rodríguez−Nieves	0.36	[0.30; 0.43]	.	0.1630	0.4037	96%
Omitting Muñoz	0.36	[0.29; 0.43]	.	0.1574	0.3968	96%
Omitting Maria	0.36	[0.30; 0.43]	.	0.1612	0.4015	96%
Omitting Kumar	0.36	[0.30; 0.43]	.	0.1623	0.4029	96%
Omitting Corrêa	0.36	[0.30; 0.43]	.	0.1620	0.4025	96%
Omitting Basurko	0.37	[0.30; 0.43]	.	0.1629	0.4037	96%
Omitting Di Maio Ferreira	0.37	[0.30; 0.43]	.	0.1630	0.4038	96%
Omitting Ferreira	0.37	[0.30; 0.43]	.	0.1630	0.4037	96%
Omitting Robillard	0.36	[0.29; 0.43]	.	0.1575	0.3969	96%
Omitting Ramful	0.36	[0.30; 0.43]	.	0.1613	0.4016	96%
Omitting Gerardin	0.36	[0.30; 0.43]	.	0.1605	0.4006	96%
Omitting Villamil−Gómez	0.36	[0.30; 0.43]	.	0.1626	0.4032	96%
Omitting Torres	0.36	[0.30; 0.43]	.	0.1609	0.4012	96%
Omitting Torres	0.36	[0.30; 0.43]	.	0.1586	0.3982	96%
Omitting Torres	0.36	[0.30; 0.43]	.	0.1632	0.4039	96%
Omitting Rodríguez−Nieves	0.36	[0.30; 0.43]	.	0.1627	0.4034	96%
Omitting Muñoz	0.36	[0.30; 0.43]	.	0.1605	0.4006	96%
Omitting Kumar	0.37	[0.30; 0.43]	.	0.1630	0.4038	96%
Omitting Di Maio Ferreira	0.37	[0.30; 0.43]	.	0.1629	0.4036	96%
Omitting Ferreira	0.37	[0.30; 0.43]	.	0.1629	0.4036	96%
Omitting Senanayake	0.37	[0.30; 0.43]	.	0.1631	0.4038	96%
Omitting Torres	0.37	[0.30; 0.44]	.	0.1618	0.4023	96%
Omitting Maria	0.36	[0.30; 0.43]	.	0.1618	0.4023	96%
Omitting Di Maio Ferreira	0.37	[0.30; 0.43]	.	0.1625	0.4032	96%
Omitting Ferreira	0.37	[0.30; 0.43]	.	0.1625	0.4031	96%
Omitting Villamil−Gómez	0.37	[0.30; 0.43]	.	0.1626	0.4032	96%
Omitting Torres	0.37	[0.30; 0.43]	.	0.1625	0.4031	96%
Omitting Torres	0.37	[0.30; 0.44]	.	0.1623	0.4028	96%
Omitting Di Maio Ferreira	0.37	[0.30; 0.43]	.	0.1625	0.4032	96%
Omitting Ferreira	0.37	[0.30; 0.43]	.	0.1625	0.4031	96%
Omitting Robillard	0.36	[0.29; 0.43]	.	0.1575	0.3969	96%
Omitting Ramful	0.36	[0.30; 0.43]	.	0.1628	0.4035	96%
Omitting Gerardin	0.36	[0.30; 0.43]	.	0.1616	0.4020	96%
Omitting Foeller	0.37	[0.30; 0.44]	.	0.1612	0.4015	96%
Omitting Villamil−Gómez	0.37	[0.30; 0.43]	.	0.1629	0.4037	96%
Omitting Torres	0.36	[0.30; 0.43]	.	0.1607	0.4009	96%
Omitting Torres	0.37	[0.30; 0.43]	.	0.1631	0.4039	96%
Omitting Torres	0.36	[0.30; 0.43]	.	0.1632	0.4039	96%
Omitting Rodríguez−Nieves	0.37	[0.30; 0.43]	.	0.1630	0.4037	96%
Omitting Gerardin	0.36	[0.29; 0.42]	.	0.1568	0.3960	96%
Omitting Sissoko	0.36	[0.30; 0.43]	.	0.1628	0.4035	96%
Omitting Villamil−Gómez	0.36	[0.30; 0.43]	.	0.1629	0.4036	96%
Omitting Torres	0.36	[0.30; 0.43]	.	0.1594	0.3992	96%
Omitting Torres	0.37	[0.30; 0.43]	.	0.1626	0.4033	96%
Omitting Torres	0.36	[0.30; 0.43]	.	0.1628	0.4035	96%
Omitting Rodríguez−Nieves	0.36	[0.30; 0.43]	.	0.1630	0.4037	96%
Omitting Gerardin	0.36	[0.29; 0.42]	.	0.1568	0.3960	96%
Omitting Rodríguez−Nieves	0.37	[0.30; 0.43]	.	0.1630	0.4037	96%
Omitting Maria	0.36	[0.29; 0.43]	.	0.1572	0.3965	96%
Omitting Kumar	0.36	[0.30; 0.43]	.	0.1631	0.4039	96%
Omitting Robillard	0.36	[0.29; 0.43]	.	0.1575	0.3969	96%
Omitting Torres	0.36	[0.30; 0.43]	.	0.1601	0.4002	96%
Omitting Rodríguez−Nieves	0.36	[0.30; 0.43]	.	0.1627	0.4034	96%
Omitting Muñoz	0.36	[0.30; 0.43]	.	0.1621	0.4026	96%
Omitting Corrêa	0.37	[0.30; 0.43]	.	0.1626	0.4032	96%
Omitting Di Maio Ferreira	0.37	[0.30; 0.43]	.	0.1625	0.4032	96%
Omitting Ramful	0.37	[0.30; 0.43]	.	0.1632	0.4039	96%
Omitting Torres	0.37	[0.30; 0.43]	.	0.1629	0.4036	96%
Omitting Torres	0.37	[0.30; 0.44]	.	0.1623	0.4028	96%
Omitting Kumar	0.37	[0.30; 0.44]	.	0.1621	0.4026	96%
Omitting Robillard	0.36	[0.29; 0.43]	.	0.1575	0.3969	96%
Omitting Gerardin	0.36	[0.30; 0.43]	.	0.1605	0.4006	96%
Omitting Torres	0.37	[0.30; 0.44]	.	0.1616	0.4020	96%
Omitting Rodríguez−Nieves	0.37	[0.30; 0.43]	.	0.1630	0.4037	96%
Omitting Maria	0.36	[0.30; 0.43]	.	0.1602	0.4003	96%
Omitting Kumar	0.36	[0.30; 0.43]	.	0.1630	0.4037	96%
Omitting Corrêa	0.37	[0.30; 0.43]	.	0.1626	0.4032	96%
Omitting Senanayake	0.37	[0.30; 0.43]	.	0.1626	0.4033	96%
Omitting Villamil−Gómez	0.36	[0.30; 0.43]	.	0.1629	0.4036	96%
Omitting Torres	0.37	[0.30; 0.44]	.	0.1622	0.4028	96%
Omitting Torres	0.37	[0.30; 0.44]	.	0.1624	0.4030	96%
Omitting Rodríguez−Nieves	0.36	[0.30; 0.43]	.	0.1630	0.4037	96%
Omitting Kumar	0.36	[0.30; 0.43]	.	0.1631	0.4039	96%
Omitting Basurko	0.37	[0.30; 0.43]	.	0.1626	0.4032	96%
Omitting Di Maio Ferreira	0.37	[0.30; 0.43]	.	0.1629	0.4036	96%
Omitting Ferreira	0.37	[0.30; 0.43]	.	0.1629	0.4036	96%
Omitting Torres	0.37	[0.30; 0.44]	.	0.1622	0.4027	96%
Omitting Rodríguez−Nieves	0.36	[0.30; 0.43]	.	0.1630	0.4037	96%
Omitting Maria	0.37	[0.30; 0.43]	.	0.1627	0.4034	96%
Omitting Di Maio Ferreira	0.37	[0.30; 0.44]	.	0.1623	0.4028	96%
Omitting Ferreira	0.37	[0.30; 0.43]	.	0.1625	0.4031	96%
Omitting Robillard	0.36	[0.30; 0.43]	.	0.1630	0.4038	96%
Omitting Gerardin	0.36	[0.30; 0.43]	.	0.1630	0.4038	96%
Omitting Sissoko	0.37	[0.30; 0.43]	.	0.1630	0.4038	96%
Omitting Senanayake	0.37	[0.30; 0.43]	.	0.1626	0.4033	96%
Omitting Villamil−Gómez	0.37	[0.30; 0.43]	.	0.1629	0.4037	96%
Omitting Torres	0.37	[0.30; 0.44]	.	0.1622	0.4027	96%
Omitting Torres	0.37	[0.30; 0.44]	.	0.1609	0.4012	96%
Omitting Rodríguez−Nieves	0.36	[0.30; 0.43]	.	0.1596	0.3995	96%
Omitting Maria	0.37	[0.30; 0.43]	.	0.1630	0.4037	96%
Omitting Kumar	0.37	[0.30; 0.43]	.	0.1629	0.4036	96%
Omitting Corrêa	0.36	[0.30; 0.43]	.	0.1620	0.4025	96%
Omitting Di Maio Ferreira	0.37	[0.30; 0.43]	.	0.1631	0.4038	96%
Omitting Torres	0.37	[0.30; 0.44]	.	0.1614	0.4018	96%
Omitting Kumar	0.36	[0.30; 0.43]	.	0.1630	0.4037	96%
Omitting Corrêa	0.36	[0.30; 0.43]	.	0.1630	0.4037	96%
Omitting Basurko	0.37	[0.30; 0.44]	.	0.1610	0.4012	96%
Omitting Basurko	0.37	[0.30; 0.44]	.	0.1606	0.4007	96%
Omitting Di Maio Ferreira	0.37	[0.30; 0.43]	.	0.1628	0.4034	96%
Omitting Ferreira	0.37	[0.30; 0.43]	.	0.1627	0.4034	96%
Omitting Maria	0.36	[0.30; 0.43]	.	0.1630	0.4037	96%
Omitting Villamil−Gómez	0.36	[0.30; 0.43]	.	0.1630	0.4037	96%
Omitting Corrêa	0.37	[0.30; 0.43]	.	0.1626	0.4032	96%
Omitting Fritel	0.37	[0.30; 0.44]	.	0.1614	0.4017	96%
Omitting Foeller	0.37	[0.30; 0.44]	.	0.1612	0.4015	96%
Omitting Escobar	0.37	[0.30; 0.43]	.	0.1623	0.4028	96%
Omitting Maria	0.37	[0.30; 0.44]	.	0.1622	0.4028	96%
Omitting Basurko	0.37	[0.30; 0.44]	.	0.1621	0.4026	96%
Omitting Sagay	0.37	[0.30; 0.44]	.	0.1617	0.4021	96%
Omitting Villamil−Gómez	0.36	[0.30; 0.43]	.	0.1626	0.4032	96%
Omitting Torres	0.37	[0.30; 0.44]	.	0.1612	0.4015	96%
Omitting Torres	0.37	[0.30; 0.44]	.	0.1619	0.4024	96%
Omitting Torres	0.37	[0.30; 0.44]	.	0.1609	0.4012	96%
Omitting Kumar	0.37	[0.30; 0.43]	.	0.1629	0.4036	96%
Omitting Senanayake	0.37	[0.30; 0.43]	.	0.1629	0.4036	96%
Omitting Kumar	0.37	[0.30; 0.43]	.	0.1630	0.4038	96%
Omitting Kumar	0.37	[0.30; 0.43]	.	0.1629	0.4036	96%
Omitting Sissoko	0.37	[0.30; 0.43]	.	0.1625	0.4031	96%
Omitting Maria	0.36	[0.29; 0.43]	.	0.1572	0.3965	96%
Omitting Ferreira	0.37	[0.30; 0.43]	.	0.1631	0.4038	96%
Omitting Sissoko	0.37	[0.30; 0.43]	.	0.1629	0.4036	96%
Omitting Dorléans	0.37	[0.30; 0.43]	.	0.1630	0.4038	96%
Omitting Sagay	0.37	[0.30; 0.44]	.	0.1617	0.4021	96%
Omitting Senanayake	0.37	[0.30; 0.43]	.	0.1626	0.4033	96%
Omitting Kumar	0.37	[0.30; 0.43]	.	0.1626	0.4032	96%
Omitting Gérardin	0.36	[0.30; 0.43]	.	0.1630	0.4037	96%
Omitting Maria	0.37	[0.30; 0.43]	.	0.1631	0.4038	96%
Omitting Rodríguez−Nieves	0.37	[0.30; 0.43]	.	0.1630	0.4037	96%
Omitting Kumar	0.37	[0.30; 0.44]	.	0.1621	0.4026	96%
Omitting Senanayake	0.37	[0.30; 0.43]	.	0.1626	0.4033	96%
Omitting Villamil−Gómez	0.36	[0.30; 0.43]	.	0.1630	0.4037	96%
Omitting Maria	0.36	[0.29; 0.43]	.	0.1572	0.3965	96%
Omitting Kumar	0.36	[0.30; 0.43]	.	0.1628	0.4035	96%
Omitting Corrêa	0.37	[0.30; 0.43]	.	0.1629	0.4037	96%
Omitting Fritel	0.37	[0.30; 0.44]	.	0.1607	0.4008	95%
Omitting Villamil−Gómez	0.36	[0.30; 0.43]	.	0.1630	0.4037	96%
Omitting Torres	0.37	[0.30; 0.44]	.	0.1618	0.4023	96%
Omitting Kumar	0.37	[0.30; 0.44]	.	0.1621	0.4026	96%
Omitting Sagay	0.37	[0.30; 0.43]	.	0.1625	0.4031	96%
**Random effects model**	**0.36**	**[0.30; 0.43]**	**.**	**0.1619**	**0.4023**	**96%**

Figure S14. Publication bias analysis of the positive rates of adverse neonatal outcomes among CHIKV-infected pregnant women.

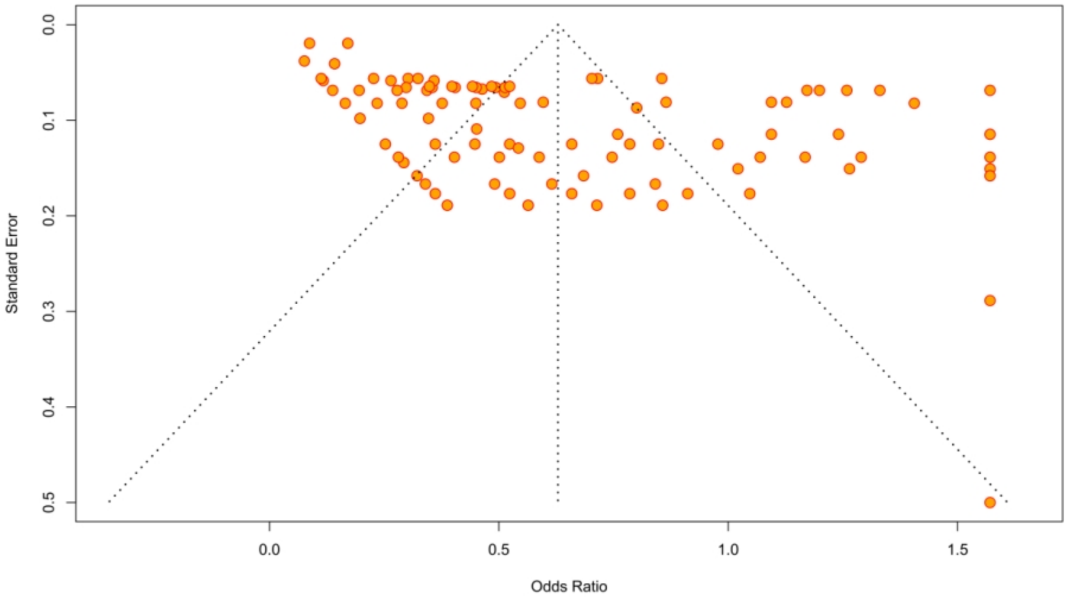

Figure S15. Leave-one-out sensitivity analysis comparing adverse pregnancy and neonatal outcomes in CHIKV-infected and uninfected pregnant women.

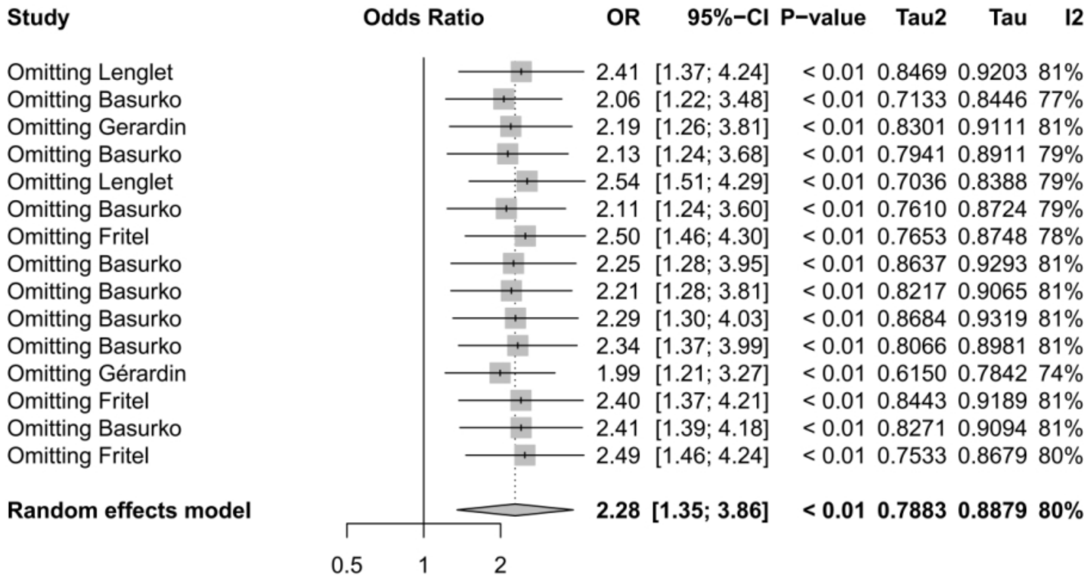

Figure S16. Publication bias analysis comparing adverse pregnancy and neonatal outcomes in CHIKV-infected and uninfected pregnant women.

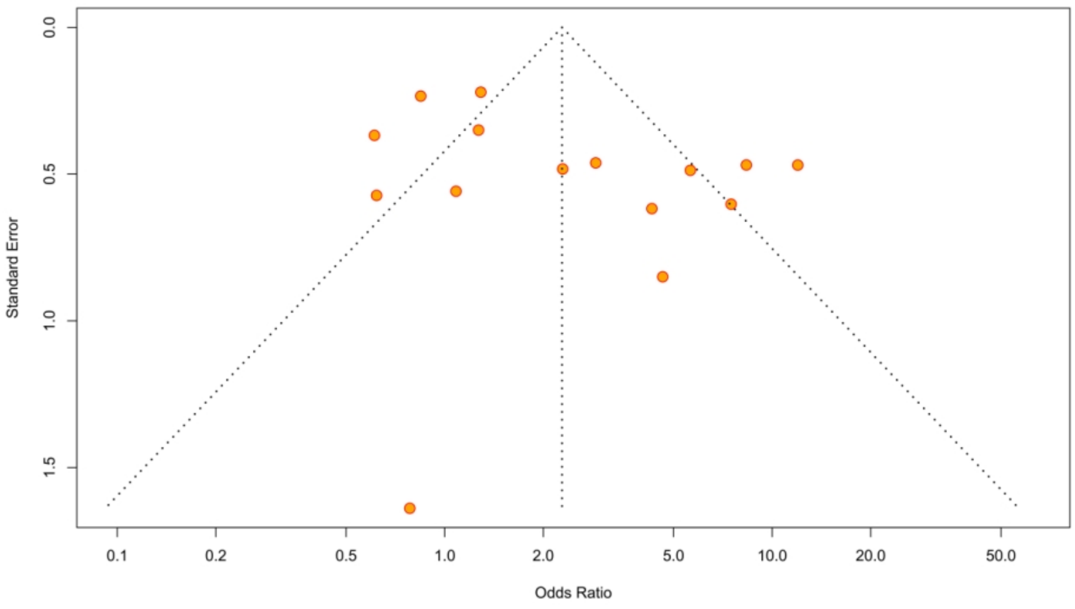

Table S1. Search strategy.

**Search strategy in PubMed**		
#1	("Chikungunya virus"[Mesh] OR "Chikungunya Fever"[Mesh] OR chikungunya OR CHIKV OR "chikungunya infection" OR "chikungunya disease"OR "arbovirus infection" OR "mosquito-borne infection")	9,556
#2	("Pregnant Women"[Mesh] OR pregnancy OR pregnant OR "maternal infection" OR "maternal outcome" OR "pregnancy complication" OR "perinatal infection" OR "obstetric complication")	1,281,234
#3	("Infant, Newborn"[Mesh] OR fetus OR fetal OR neonatal OR newborn OR perinatal OR "vertical transmission" OR "mother-to-child transmission" OR "congenital infection" OR "intrauterine infection" OR placenta OR placental OR "amniotic fluid" OR "umbilical cord" OR "cord blood" OR "fetal membrane" OR "fetal membranes" OR "fetal outcomes" OR "neonatal outcomes" OR "perinatal outcomes" OR "pregnancy outcome" OR stillbirth OR miscarriage OR abortion OR preterm OR "preterm birth" OR "low birth weight" OR LBW OR "neonatal death" OR "infant death" OR mortality)	3,429,236
#4	#1 AND #2 AND #3	185
**Search strategy in Embase**		
#1	('chikungunya virus'/exp OR 'chikungunya fever'/exp OR chikungunya OR CHIKV OR 'arbovirus infection'/exp OR 'mosquito-borne disease'/exp)	13,846
#2	('pregnancy'/exp OR 'pregnant woman'/exp OR 'maternal infection'/exp OR 'pregnancy complication'/exp OR 'obstetric complication'/exp OR 'perinatal infection'/exp)	1,785,602
#3	('infant'/exp OR 'newborn'/exp OR 'fetus'/exp OR 'neonate'/exp OR 'vertical transmission'/exp OR 'mother to child transmission'/exp OR 'congenital infection'/exp OR 'intrauterine infection'/exp OR 'placenta'/exp OR 'amniotic fluid'/exp OR 'umbilical cord'/exp OR 'cord blood'/exp OR 'fetal membrane'/exp OR 'pregnancy outcome'/exp OR stillbirth OR miscarriage OR abortion OR preterm OR 'low birth weight' OR 'neonatal death' OR 'infant death' OR mortality)	4,272,765
#4	#1 AND #2 AND #3	216
**Search strategy in Cochrane Library**		
#1	MeSH descriptor: [Chikungunya virus] explode all trees OR (chikungunya OR CHIKV OR "arboviral infection" OR "mosquito-borne infection")	5,591
#2	MeSH descriptor: [Pregnancy] explode all trees OR (pregnancy OR pregnant OR "maternal infection" OR "pregnancy complication" OR "obstetric complication")	89,601
#3	MeSH descriptor: [Infant, Newborn] explode all trees OR (fetal OR neonatal OR placenta OR "amniotic fluid" OR "umbilical cord" OR "cord blood" OR "fetal membrane" OR "vertical transmission" OR "mother-to-child transmission" OR "congenital infection" OR "fetal outcomes" OR "neonatal outcomes" OR "perinatal outcomes" OR stillbirth OR miscarriage OR preterm OR "low birth weight" OR "neonatal death")	2,042,348
#4	#1 AND #2 AND #3	48
**Search strategy in Web of Science**		
#1	TS=(chikungunya OR CHIKV OR "chikungunya infection" OR "chikungunya fever" OR "arbovirus infection" OR "mosquito-borne infection")	14,489
#2	TS=(pregnancy OR pregnant OR "maternal infection" OR "pregnancy complication" OR "obstetric complication" OR "perinatal infection")	1,492,826
#3	TS=(fetus OR fetal OR neonatal OR newborn OR perinatal OR placenta OR placental OR "amniotic fluid" OR "umbilical cord" OR "cord blood" OR "fetal membrane" OR "vertical transmission" OR "mother-to-child transmission" OR "congenital infection" OR "intrauterine infection" OR "fetal outcome" OR "neonatal outcome" OR "perinatal outcome" OR stillbirth OR miscarriage OR preterm OR "low birth weight" OR "neonatal death" OR "infant death" OR mortality)	4,437,898

#4	#1 AND #2 AND #3	298

Supplementary Methods
Outcome Definitions and Harmonization Procedures

Definitions and assessment methods for pregnancy and neonatal outcomes varied considerably across the included studies. To ensure methodological transparency, all outcome data were extracted exactly as originally defined by each study, without reinterpretation or reclassification at the extraction stage.

Following extraction, outcomes were grouped into broader, conceptually similar categories to facilitate quantitative synthesis. These categories included:

Maternal outcomes (e.g., febrile illness, hematological abnormalities, obstetric complications);

Pregnancy outcomes (e.g., miscarriage, stillbirth, preterm birth, fetal growth restriction);

Neonatal outcomes (e.g., neurological complications, systemic symptoms, laboratory abnormalities).

This harmonization process involved categorizing heterogeneous outcomes into shared domains based on clinical similarity. To provide full transparency regarding the variability in outcome definitions across studies, we constructed a comprehensive outcome definition mapping table (Table S10) that lists, for each primary and secondary outcome, the specific diagnostic criteria or definitions used by each included study. This table enables readers to assess the degree of heterogeneity in outcome ascertainment and to interpret the pooled estimates in light of this variability.

Importantly, no additional standardization or redefinition of outcomes was applied, in order to avoid introducing misclassification bias or distorting original study intent. All analyses therefore reflect outcome definitions as reported in the primary studies.

Handling of overlapping cohorts

During data extraction, we identified several instances of potential cohort overlap, particularly from outbreak settings where multiple publications reported on the same population (e.g., Réunion Island 2005–2006 outbreak, Colombia 2014–2015 outbreaks). To address this, we implemented a hierarchical selection strategy:

For cohorts with multiple publications, we prioritized the report with the largest sample size or the most complete outcome data.

When reports provided complementary information (e.g., one reporting vertical transmission, another reporting neonatal outcomes), data were combined only if we could confirm non-overlapping participant populations.

In cases where overlap could not be definitively determined, we contacted study authors for clarification. If no response was received, we included the study with the most comprehensive data and excluded others to minimize potential double-counting.

This approach ensures that each participant contributed only once to each pooled analysis. The discrepancies between raw case numbers in Table S2 and the denominators used in meta-analyses reflect this deduplication process.

Clarification of selected neonatal outcomes

For neonatal outcomes that were particularly heterogeneous or loosely defined across studies, we applied the following harmonization approach:

"Abnormal crying": This outcome encompassed various descriptions including "irritability," "high-pitched cry," "excessive crying," and "inconsolable crying." All such descriptions were grouped under the single category "abnormal crying" based on their clinical similarity as indicators of neonatal neurological irritability.

"Hyperalgesia": This term was used inconsistently across studies, sometimes referring to generalized pain hypersensitivity and other times to localized pain responses. Given the limited number of studies reporting this outcome and the lack of standardized assessment tools, we retained the original terminology as reported by each study without further reclassification, and the specific definitions used are detailed in Table S10.

"Confusion": This outcome was reported infrequently and typically as part of a broader neurological assessment. In most cases, it was described alongside other neurological symptoms such as lethargy or decreased responsiveness. Due to the sparse data and variable descriptions, we grouped "confusion" with other non-specific neurological signs under the broader category of "neurological complications" in the main analysis, while preserving the original terminology in Table S10 for transparency.

This approach ensures that clinically similar manifestations were combined for quantitative synthesis while maintaining transparency about the underlying heterogeneity in definitions.

Table S2. Characteristics of included studies.

Study	Area	Study period	Study design	Detection method (mother / infants)	Gestational age at maternal infection	Vertical transmission (No. of mothers / No. Of infants)	Clinical manifestations of CHIKV infection in mothers	Pregnancy outcomes	Neonatal outcomes	Include d in meta-analysis (Y / N)	Quality assessment method(NOS / Revised NOS / Murad)	Quality assessment results (High / Moderate / Low)
Lenglet et al 2006[1]	Reunion	2005/06-2006/02	Comparative cohort	RT-PCR, IgM / RT-PCR	Early-to-mid pregnancy	160/19	Fever, rash, joint pain	Stillbirth	-	**Y**	NOS	High
Robillard et al 2006[2]	Reunion	2005/06-2006/01	Single-arm cohort	RT-PCR, IgM / RT-PCR, IgM	Mid-to-late pregnancy	94/18	Fever	-	Fever, rash, edema, abnormal crying, adrenal crisis, thrombocytopenia, abnormal EEG	Y	Revised NOS	High
Watanaveeradej et al 2006[3]	Thailand	1998/03-1999/10	Single-arm cohort	IgM, IgG / IgM, IgG	Late pregnancy	79/64	Fever, rash, joint pain	-	-	Y	Revised NOS	Moderate
Touret et al 2006[4]	Reunion	2005/06-2006/02	Case series	RT-PCR, IgM / RT-PCR, IgM	Early-to-mid pregnancy	7/3	Fever, rash, joint pain, headache	Stillbirth	-	Y	Murad	High
Ramful et al 2007[5]	Reunion	2005/03-2006/04	Single-arm cohort	RT-PCR, IgM / RT-PCR, IgM	Late-pregnancy to delivery	38/38	Fever, rash, joint pain	-	Fever, rash, edema, diarrhea	Y	Revised NOS	High
Gerardin et al 2008[6]	Reunion	2005/06-2006/12	Comparative cohort	RT-PCR, IgM, IgG / RT-PCR, IgM, IgG	Early-to-late pregnancy	739/19	Fever, rash, joint pain	Abnormal fetal heart rate	Fever, feeding difficulties, pain hypersensitivity, edema, rash, petechiae, encephalopathy, hemorrhagic fever, thrombocytopenia, lymphocytopenia	Y	NOS	High
Sissoko et al 2008[7]	Mayotte	2005/10-2006/04	Single-arm cohort	RT-PCR, IgM / RT-PCR, IgM	NS	945/9	Polyarthralgia, fever, headache	-	Meningoencephalitis, hypotonia, pain hypersensitivity, renal failure	Y	Revised NOS	High
Rao et al 2008[8]	India	2006	Case series	NS / RT-PCR	Late pregnancy	2/2	Fever, joint pain	-	Fever, rash, pigmentation, respiratory distress, apnea, thrombocytopenia, mucositis, subcutaneous fat reduction	N	Murad	High
Senanayake et al 2009[9]	Colombia	2007/04-2007/10	Single-arm cohort	IgM / IgM	Early pregnancy	50/7	Fever, joint pain	Miscarriage, stillbirth, fetal intrauterine growth restriction	Fever, pigmentation, congenital heart disease，meningoencephalitis,	Y	Revised NOS	High

									respiratory distress, developmental delay, splenomegaly, myocarditis			
Fritel et al 2010[10]	Reunion	2006/04-2006/11	Comparative cohort	RT-PCR, IgM / RT-PCR, IgM	Mid-pregnancy to delivery	3/2	Fever, arthralgia, headache, edema, diarrhea, oral ulcers, epistaxis or gingival bleeding, rash	Vaginal bleeding, obstetric hemorrhage	Congenital malformations, death	Y	NOS	High
Boumahni et al 2011[11]	Reunion	NS	Case	RT-PCR, IgM, IgG / RT-PCR, IgM, IgG	At delivery	1/1	-	-	Fever, pain, convulsions, respiratory failure, thrombocytopenia	N	Murad	High
Shrivastava et al 2011[12]	India	NS	case	RT-PCR, IgM, IgG / RT-PCR, IgM, IgG	Late pregnancy	1/1	Fever, joint pain	Abnormal fetal heart rate	Fever, rash, respiratory distress, abnormal crying, thrombocytopenia	N	Murad	High
Shenoy et al 2012[13]	India	NS	Case series	RT-PCR / RT-PCR	Late pregnancy	2/2	Fever, joint pain	-	Apnea, brain lesions, pigmentation, epilepsy, spastic diplegia, intellectual disability, primary optic atrophy, cerebral palsy, intellectual disability	N	Murad	High
Khandelwal et al 2012[14]	NS	NS	Case	IgM / IgM	Late pregnancy	1/1	Fever, joint pain, muscle pain, generalized itchy erythematous papules	-	Pigmentation	N	Murad	High
Gopakumar et al 2012[15]	India	2019	Case	IgM, IgG / IgM, IgG	Late pregnancy	1/1	Fever, joint pain, pigmentation	-	Fever, generalized edema, hyperalgesia, abnormal crying, renal failure, pigmentation, thrombocytopenia, sepsis, oral mucosal bleeding	N	Murad	High
Kumar et al 2014[16]	NS	NS	Case	IgM / IgM	Late pregnancy	1/1	Fever	-	Respiratory distress, pigmentation, thrombocytopenia, sepsis	N	Murad	High
Ramful et al 2014[17]	Reunion	2006-2008	Single-arm cohort	RT-PCR, IgM, IgG / RT-PCR, IgM, IgG	Throughout pregnancy	653/1	Fever, rash, joint pain	-	-	Y	Revised NOS	High
Gérardin et al 2014[18]	Reunion	2006/04-2006/08	Comparative cohort	RT-PCR, IgM / RT-PCR, IgM	Late-pregnancy to delivery	103/33	Fever	Premature birth	Neurodevelopmental delay	Y	NOS	High
Foeller et al 2015[19]	Grenada	2014/01-2015/09	Comparative cohort	RT-PCR, IgM, IgG /	At delivery	150/1	Fever, joint pain, muscle pain, rash,	Placental abruption, vaginal bleeding	Tracheomalacia, congenital hydrops, clubfoot	Y	NOS	High

				RT-PCR, IgM, IgG			bone pain, headache, retro-orbital pain, vomiting					
Villamil-Gómez et al 2015[20]	Colombia	2014/09-2015/02	Case series	RT-PCR, IgM, IgG / RT-PCR, IgM, IgG	NS	7/8	Fever, joint pain, rash, edema, headache	-	Maculopapular rash, hyperalgesia, respiratory distress, sepsis, necrotizing enterocolitis, lymphadenopathy, meningoencephalitis, myocarditis, edema, bullous dermatitis, pericarditis, pericardial effusion and mild atrial septal defect, pulmonary hypertension, death	Y	Murad	High
Taksande et al 2015[21]	India	NS	Case	NS / IgM	Late pregnancy	1/1	Fever, generalized soreness, headache	-	Fever, abnormal crying, thrombocytopenia	N	Murad	High
Vasani et al 2016[22]	India	NS	Case	IgM, IgG / IgM, IgG	Late pregnancy	1/1	Fever	-	Pigmentation, respiratory distress	N	Murad	High
Laoprasopwattana et al 2016[23]	Thailand	2006/04-2006/08	Comparative cohort	RT-PCR, IgM / RT-PCR, IgM	Late-pregnancy to delivery	10/0	Fever, rash, joint pain	-	Normal	Y	NOS	Moderate
Torres et al 2016[24]	El Salvador, Colombia, Dominican Republic	2014/08-2014/10	Single-arm cohort	RT-PCR, IgM / RT-PCR	Late pregnancy	433/189	Fever, joint pain	Premature birth	Fever, abnormal crying, rash, hyperalgesia, pigmentation, generalized edema, thrombocytopenia, hemodynamic instability, bullous dermatitis/desquamation, respiratory failure, meningoencephalitis, myocarditis, diarrhea, respiratory distress, abdominal pain, seizures	Y	Revised NOS	High
Alvarado-Socarras et al 2016[25]	Colombia	NS	Case series	NS / IgM	Late pregnancy	2/2	Fever, edema, pelvic pain, maculopapular rash	-	Fever, maculopapular rash, jaundice, distal cyanosis, abnormal crying, thrombocytopenia, seizures, hypotonia, lymphocytopenia	N	Murad	High
Bandeira et al 2016[26]	Brazil	2015/11	Case	NS / RT-PCR	Late pregnancy	1/1	Fever, rash	-	Fever , rash, seizures, encephalitis	N	Murad	High
Karthiga et al 2016[27]	India	NS	Case series	IgM / IgM	At delivery	2/4	Fever	-	Seizures, encephalopathy, pigmentation, anemia, thrombocytopenia, hypocalcemia, apnea, uremia	N	Murad	High
Rodríguez-Niev	American	2014/08-2	Single-arm	NS / IgM	Late	NS/10	Fever, rash, joint	-	Fever, abnormal crying, rash,	Y	Revised	High

es et al 2016[28]		015/01	cohort		pregnancy		pain		general discomfort, apnea, respiratory distress, poor sucking ability, cyanosis, generalized edema, leukopenia, leukocytosis, thrombocytopenia, hypoalbuminemia, hydrocephalus, cerebral infarction		NOS	
Nigam et al 2016[29]	India	NS	case	IgM / RT-PCR	Late pregnancy	1/1	Fever, joint pain	Pericardial effusion, fetal growth restriction	Respiratory distress, pericardial effusion	N	Murad	High
Lyra et al 2016[30]	Brazil	2025/08	Case series	RT-PCR / RT-PCR	Late pregnancy	2/2	Fever, rash, joint pain	-	Fever, rash, respiratory distress, hepatosplenomegaly, generalized edema, pneumonia, drowsiness, sepsis, cerebral hemorrhage	N	Murad	High
Muñoz et al 2016[31]	Colombia	2014/12	Single-arm cohort	NS / RT-PCR、IgM	NS	11/4	Fever, joint pain	-	Fever, rash, abnormal crying	Y	Revised NOS	Moderate
Escobar et al 2017[32]	Colombia	2015/01-2015	Single-arm cohort	RT-PCR / RT-PCR	Early-to-late pregnancy	75/0	Fever, joint pain, rash, myalgia, leukopenia, thrombocytopenia.	Miscarriage, sepsis, premature rupture of membranes, preeclampsia, fetal growth restriction, preterm birth, postpartum hemorrhage	Fever, rash, abnormal crying, leukocytosis, lymphocytosis), Turner syndrome	Y	Revised NOS	High
Evans-Gilbert et al 2017[33]	Jamaica	2014	Case series	RT-PCR / RT-PCR	Late pregnancy	2/2	Fever, joint pain，thrombocytopenia, leukocytosis, disseminated intravascular coagulation, renal failure	-	Fever, drowsiness, rash, poor feeding, respiratory distress, cyanosis, apnea, abdominal distension, cyanosis, reduced peripheral perfusion, leukopenia, thrombocytopenia, rectal prolapse, death	N	Murad	High
Cardona-Correa et al 2017[34]	Colombia	NS	case	NS / IgM	At delivery	1/1	Fever, joint pain	-	Fever, rash, jaundice, sepsis, thrombocytopenia	N	Murad	High
Ramos et al 2018[35]	Brazil	2016/02	Case	IgM / RT-PCR, IgM	Late pregnancy	1/1	Rash, joint pain, headache	Premature birth	Rash, hand and foot edema, abnormal crying, respiratory failure, sepsis, encephalitis, cerebral hemorrhage, cerebral edema, hydrocephalus, epilepsy	N	Murad	High
Maria et al 2018[36]	India	2016/08-2016/11	Case series	RT-PCR, IgM / RT-PCR, IgM	Late pregnancy	99/13	Fever	-	Fever, drowsiness, refusal to eat, convulsions, hyperpigmentation, hypotonia, leukopenia, thrombocytopenia, shock, neurodevelopmental delay, finger deformities, cerebral softening and	Y	Murad	High

									ventricular dilation, cerebral atrophy			
Oliveira et al 2018[37]	Brazil	2017	case	NS / IgM	Late pregnancy	1/1	Fever, joint pain, headache, knee and ankle arthritis, cough, nausea, abdominal pain, diarrhea	Preeclampsia, respiratory failure, peripartum cardiomyopathy, death	Fever, respiratory distress, cyanosis, thrombocytopenia, jaundice, apnea, multiple organ hemorrhage, epilepsy, generalized edema, jaundice, cerebral edema, seizure, death	N	Murad	High
Enter et al 2018[38]	Curaçao	2014/09-2015/03	Case series	IgM / IgM, IgG	Late pregnancy	60/3	Fever, rash, joint pain	Abnormal fetal heart rate, fetal distress	Fever, rash, pain sensitivity, abnormal crying, feeding difficulties, convulsions, cerebral hemorrhage, death	Y	Murad	High
Dorléans et al 2018[39]	Martinique, Guadeloupe	2013/12-2015/01	Single-arm cohort	RT-PCR, IgM / RT-PCR, IgM	Late-pregnancy to delivery	185/15	Fever	-	Severe clinical manifestations, heart failure	Y	Revised NOS	High
Kumar et al 2019[40]	India	2016/08-2016/11	Case series	RT-PCR / RT-PCR, IgM	NS	16/6	Fever, rash, joint pain	Premature birth	Fever, drowsiness, epilepsy, respiratory distress, feeding difficulties, skin lesions, cardiovascular involvement, encephalopathy, hyperbilirubinemia, diarrhea, cyanosis, myocarditis, thrombocytopenia, pulmonary hypertension, developmental delay with microcephaly, death	Y	Murad	High
Di Maio Ferreira et al 2019[41]	Brazil	NS	Case	RT-PCR, IgM / RT-PCR, IgM	Late pregnancy	1/1	Fever, rash, joint pain	Fetal distress	Fever, skin lesions, abnormal crying, edema, hypoglycemia, reduced activity	N	Murad	High
Chandramathi et al 2020[42]	India	NS	case	NS / IgM	Late pregnancy	1/1	Fever	-	Pigmentation, apnea, sepsis, hyperbilirubinemia, thrombocytopenia	N	Murad	High
Corrêa et al 2020[43]	Brazil	NS	Case series	RT-PCR, IgM / RT-PCR, IgM	Late pregnancy	8/8	Fever, rash, joint pain	-	Fever, brain lesions, epilepsy, abnormal crying, thrombocytopenia, sepsis, impaired consciousness	Y	Murad	High
Jebain et al 2020[44]	Brazil	NS	case	RT-PCR, IgM, IgG / RT-PCR, IgM, IgG	Postpartum	1/1	Fever, joint pain	-	Fever, rash, pigmentation	N	Murad	High
Goulart Corrêa et al 2020[45]	Brazil	NS	Case series	RT-PCR, IgM / IgM	Late pregnancy	3/3	Fever, rash, joint pain	-	Fever, thrombocytopenia, feeding difficulties (1/3), brain lesions, decreased level of consciousness, convulsions	N	Murad	High

Shen et al 2021[46]	China	1905/07	Case series	RT-PCR / RT-PCR	At delivery	2/3	Fever, rash, joint pain, muscle pain	Cloudy amniotic fluid	Fever, rash, joint pain, jaundice	N	Murad	High
Salomão et al 2021[47]	Brazil	NS	Case series	RT-PCR / Immunohistochemistry	Early pregnancy	4/4	Fever, joint pain, rash, and itching	Miscarriage	-	N	Murad	Moderate
Basurko et al 2022[48]	Guiana	2012/06-2015/06	Comparative cohort	RT-PCR, IgM, IgG / NS	Early-to-late pregnancy	73/9	Fever, rash, joint pain, headache, myalgia, digestive system symptoms, weakness, generalized pain	Preterm birth, postpartum hemorrhage, fetal growth restriction, abnormal fetal heart rate	Fever, respiratory distress, epilepsy, congenital malformations	Y	NOS	High
Salomão et al 2022[49]	Brazil	2019	Case	RT-PCR, IgM / RT-PCR, IgM	Late pregnancy	1/1	Fever, rash, joint pain，lower limb swelling, erythematous lesions, bullous lesions	Reduced amniotic fluid	Fever, hypoglycemia, respiratory distress	N	Murad	High
Di Maio Ferreira et al 2024[50]	Brazil	2021/07	Case series	RT-PCR / RT-PCR	At delivery	58/18	Fever, joint pain, rash, arthritis, myalgia, itching, cholestasis	Preeclampsia , urinary tract infection, epilepsy, urinary sepsis	Fever, seizures and encephalitis, rash, respiratory distress, apnea, abnormal crying, pigmentation, bullous lesions, low body weight	Y	Murad	High
Tun et al 2024[51]	Vietnam	2017/09-2018/09	Single-arm cohort	NS / RT-PCR, IgM	NS	NS/18	Fever	-	Vomiting	N	Revised NOS	High
Sagay et al 2024[52]	Nigeria	2019/04-2022/01	Single-arm cohort	RT-PCR, IgM, IgG / RT-PCR, IgM, IgG	Early-to-late pregnancy	26/13	Fever, headache, fatigue	Preterm birth	Congenital malformations, sepsis, death	Y	Revised NOS	High
Faria et al 2024[53]	Brazil	NS	Case series	IgM, IgG / RT-PCR, IgM	Late pregnancy	2/2	Fever, muscle pain, joint pain, lymphocytopenia, itching, diffuse petechiae, lower limb pain, leukopenia, erysipelas	Reduced fetal movement, preterm birth	Fever, feeding difficulties, hematuria, rash, abnormal crying, pigmentation, respiratory distress, rash, epilepsy, intracranial hemorrhage, death	N	Murad	High
Pedrosa Do Monte et al 2025[54]	Brazil	2015-2016	Case series	RT-PCR IgM / RT-PCR, IgM	early pregnancy	4/5	Fever, rash, joint pain, headache, edema, vomiting, pelvic pain, itching, pain in the lower limbs and hands	Fetal distress	Pulmonary and hepatic congestion, cerebral congestion, pleural effusion, death	N	Murad	High

Pinheiro de Carvalho et al 2025[55]	Brazil	2015-2017	Single-arm cohort	NS / Immunohistochemistry	NS	26/10	Fever, headache, vomiting	Miscarriage	-	Y	Revised NOS	Moderate
Ferreira et al 2025[56]	Brazil	NS	Single-arm cohort	RT-PCR, IgM / RT-PCR, IgM	Early-late pregnancy	57/17	-	Fetal distress	Fever, rash, respiratory distress, pigmentation, blistering lesions, apnea, reduced activity, epilepsy	Y	Revised NOS	High
Yin et al 2025[57]	China	1905/07	Case series	RT-PCR / RT-PCR	late pregnancy	2/3	Fever	-	Fever	N	Murad	Moderate

Abbreviations: NOS, Newcastle–Ottawa Scale; EEC, electroencephalogram; Y, yes; N, no; No., number; NS, not specified. *Outcome definitions were extracted as originally reported in each study; definitions varied across studies and were harmonized into broader categories for analysis.

Note: The numbers presented in the "Vertical transmission (No. of mothers / No. of infants)" column represent the raw data as reported in each original study. However, for studies identified as overlapping reports from the same cohort (e.g., multiple publications from the Réunion Island outbreak), only the most comprehensive report was included in the meta-analysis to avoid double-counting. Consequently, the total number of participants in pooled analyses may differ from the sum of individual study sample sizes in this table. Detailed information on cohort overlaps and deduplication decisions is provided in the Supplementary Methods.

Table S3. The quality evaluation of all studies.

Observational Studies (n = 22) (Assessed using the NOS / Revised NOS)	N (%)	Case Reports (n = 16) and Case Series (n = 19) (Assessed using the Murad Tool)	N (%)
Selection		All Studies (Case Reports & Series)	
Representativeness of the exposed	22 (100)	1. Causality: Clear temporal sequence between exposure and outcome?	35 (100)
Virus positive and negative from the same source population	6 (31·9)	2. Were the patient's baseline characteristics (demographics, medical history, etc.) sufficiently described?	31 (88·6)
Ascertainment of exposure	21 (95·5)	3. Was the clinical course sufficiently described and continuous?	34 (97·1)
Outcome not present at start	22 (100)	4. Was the adverse event (outcome) measured using a reliable method?	35 (100)
Comparability			
Comparability on most important factors	7 (31·9)	Case Series Only	
Comparability on other risk factors	4 (18·2)	5. Were the cases collected/selected in a way that minimized selection bias (e.g., consecutive)?	19 (100)
Outcome		6. Was the exposure sufficiently described and measured?	19 (100)
Ascertainment of outcome	22 (100)	7. Was the outcome measured consistently and adequately for all cases?	17 (89·5)
Long enough follow-up	20 (90·1)	8. Were the statistical methods sufficiently reported and appropriate?	11 (57·9)
Adequacy of follow-up	20 (90·1)		

Table S4. Vertical transmission rates of CHIKV among pregnant women infected at different gestational stages.

	Studies (k)	Virus infection		
		Participants (n)	Pooled proportion （95% CI ）	I^2 (%)
Studies	24	883	0.266 (0.108 to 0.465)	95
Stage				
Early pregnancy	3	28	0.039 (0.000 to 0.186)	3
Mid pregnancy	3	40	0.012 (0.000 to 0.087)	0
Late pregnancy	10	543	0.365 (0.100 to 0.684)	96
At delivery	8	272	0.491 (0.118 to 0.870)	95

Vertical transmission was defined according to each study's laboratory-confirmed diagnostic criteria (RT-PCR and/or neonatal IgM detection)..

Table S5. Vertical transmission rates of CHIKV among pregnant women across different regions.

	Studies (k)	Virus infection		
		Participants (n)	Pooled proportion （95% CI ）	I^2 (%)
Studies	23	4100	0.181 (0.096 to 0.287)	98
Region				
Reunion	7	1759	0.158 (0.041 to 0.331)	97
Thailand	2	89	0.291 (0.000 to 1.000)	98
Mayotte	1	945	0.010 (0.004 to 0.018)	-
Colombia	3	196	0.101 (0.000 to 0.403)	94
Grenada	1	150	0.007 (0.000 to 0.037)	-
El Salvador	1	433	0.436 (0.389 to 0.485)	-
India	2	115	0.223 (0.045 to 0.486)	78
Curaçao	1	61	0.049 (0.010 to 0.137)	-
Martinique, Guadeloupe	1	185	0.081 (0.046 to 0.130)	-
Brazil	3	141	0.319 (0.245 to 0.398)	0
Nigeria	1	26	0.500 (0.299 to 0.701)	-

Vertical transmission was defined according to each study's laboratory-confirmed diagnostic criteria (RT-PCR and/or neonatal IgM detection).

Table S6. Adverse pregnancy outcomes following maternal CHIKV infection (overall and by outcome).
Overall (≥1 adverse pregnancy outcome): k = 29 outcome reports; n = 2313 participants; pooled incidence = 0.113 (95% CI 0.069–0.167).

Outcome	Outcome reports (k)	Virus infection		
		Participants (n)	Pooled proportion (95% CI)	I^2 (%)
**Overall (≥ 1 outcome)**	29	2313	0.113 (0.069 to 0.167)	85
Miscarriage	3	86	0.029 (0.004 to 0.075)	14
Preterm birth	6	529	0.101 (0.037 to 0.193)	83
Stillbirth	3	37	0.220 (0.030 to 0.517)	72
Abnormal fetal heart rate	4	139	0.449 (0.198 to 0.715)	89
Fetal growth restriction	5	343	0.065 (0.031 to 0.111)	48
Preeclampsia	2	118	0.102 (0.054 to 0.162)	0
Postpartum hemorrhage	3	791	0.077 (0.031 to 0.140)	72
Sepsis	1	60	0.017 (0.000 to 0.089)	-
Placental abruption	1	150	0.007 (0.000 to 0.037)	-
Premature rupture of membranes	1	60	0.050 (0.010 to 0.139)	-

Note: Outcome definitions varied across studies; outcomes were synthesized using original study definitions grouped into broader categories.

Table S7. Adverse neonatal outcomes following maternal CHIKV infection (overall and by outcome).
Overall (≥1 adverse neonatal outcome): k = 144 outcome reports; n = 5880 neonates; pooled incidence = 0.365 (95% CI 0.300–0.432).

Outcome	Outcome reports (k)	Virus infection		
		Participants (n)	Pooled proportion (95% CI)	I^2 (%)
**Overall (≥ 1 outcome)**	144	5880	0.365 (0.300 to 0.432)	96
Fever	13	349	0.689 (0.436 to 0.892)	96
Rash	12	396	0.677 (0.462 to 0.859)	95
Pigmentation	5	260	0.218 (0.046 to 0.469)	89
Bullous skin disease	5	258	0.108 (0.073 to 0.149)	0
Edema	9	401	0.512 (0.244 to 0.776)	97
Hyperalgesia	7	212	0.643 (0.341 to 0.893)	95
Feeding difficulties	4	55	0.794 (0.279 to 1.000)	94
Abnormal crying	6	147	0.617 (0.240 to 0.926)	96
Diarrhea	4	170	0.156 (0.065 to 0.279)	72
Thrombocytopenia	7	126	0.572 (0.205 to 0.897)	95
Respiratory distress	9	345	0.176 (0.116 to 0.246)	53
Brain lesions	17	474	0.234 (0.135 to 0.352)	81
Epilepsy	8	438	0.148 (0.040 to 0.309)	91
Sepsis	2	16	0.238 (0.047 to 0.517)	29
Cardiac lesions	13	1162	0.076 (0.035 to 0.133)	71
Pulmonary hypertension	1	16	0.188 (0.040 to 0.456)	-
Decreased muscle tone	3	82	0.526 (0.004 to 1.000)	96
Organ failure	3	50	0.145 (0.025 to 0.341)	63
Delayed physical development	2	23	0.130 (0.027 to 0.295)	0
Delayed neurodevelopment	2	46	0.436 (0.247 to 0.636)	41
Cyanosis	2	23	0.135 (0.006 to 0.390)	47
Splenomegaly	1	7	0.143 (0.004 to 0.579)	-
Lymphadenopathy	1	8	0.375 (0.085 to 0.755)	-
Confusion	3	37	0.696 (0.140 to 1.000)	92
Death	5	787	0.069 (0.012 to 0.167)	82

Note: Outcomes were extracted using each study's original definitions and grouped into broader categories for synthesis because definitions and assessment methods varied across studies.

Table S8. Comparison of adverse pregnancy and neonatal outcomes between pregnant women infected and uninfected with CHIKV.

Outcome	Studies (k)	CHIKV infection group			CHIKV non-infection group			Odds ratios (OR)	I^2 (%)	Confounders adjusted for	Adjustment method
		Participants (n)	Pooled proportion (%)	I^2 (%)	Participants (n)	Pooled proportion (%)	I^2 (%)				
Overall (≥ 1 outcome)	15	2912	12.9	95	10833	5.5	96	2.283 (1.351 to 3.856)	80		
Pregnancy outcomes											
Preterm birth	2	3851	20.8	56	3851	8.5	95	3.133 (0.504 to 19.489)	92		
Lenglet et al 2006		151	17.2		3678	13.9		1.289 (0.836 to 1.987)		Maternal age, parity, socioeconomic status	Multivariate logistic regression
Basurko et al 2022		73	26.0		173	4.0		8.344 (3.327 to 20.924)		Maternal age, gestational age at delivery, pre-existing medical conditions	Multivariate logistic regression (nested case-control)
Abnormal fetal heart rate	2	92	44.9	95	211	17.8	96	5.073 (2.397 to 10.735)	0		
Gerardin et al 2008		19	73.7		38	39.5		4.293 (1.279 to 14.408)		Maternal age, parity, mode of delivery	Multivariate logistic regression
Basurko et al 2022		73	19.2		173	4.0		5.627 (2.166 to 14.618)		Maternal age, gestational age at delivery, pre-existing medical conditions	Multivariate logistic regression
Fetal growth restriction	2	224	9.3	82	3851	5.1	92	2.044 (0.175 to 23.845)	92		
Lenglet et al 2006		151	5.3		3678	8.4		0.610 (0.296 to 1.255)		Maternal age, parity, mode of	Multivariate logistic regression
Basurko et al 2022		73	15.1		173	2.3		7.496 (2.302 to 24.414)		Maternal age, gestational age at delivery, pre-existing medical conditions	Multivariate logistic regression (nested case-control)
Postpartum hemorrhage	2	731	9.2	86	828	6.3	0	1.466 (0.442 to 4.863)	82		
Fritel et al 2010		658	5.5		655	6.4		0.845 (0.534 to 1.337)		Maternal age, gestational age at mode of delivery	Multivariate logistic regression
Basurko et al 2022		73	15.1		173	5.8		2.892 (1.170 to 7.148)		Maternal age, gestational age at delivery, mode of delivery	Multivariate logistic regression (nested case-control)
Neonatal outcomes											
Fever	1	128	6.8	-	128	1.6	-	4.632 (0.875 to 24.531)	-		
Basurko et al 2022										Maternal age, gestational age at delivery, mode of delivery	Multivariate logistic regression (nested case-control)

Respiratory distress	1	173	12.3	-	173	5.8	-	2.292 (0.890 to 5.902)	-		
Basurko et al 2022										Maternal age, gestational age at delivery, mode of delivery	Multivariate logistic regression (nested case-control)
Epilepsy	1	173	0	-	173	0.6	-	0.782 (0.032 to 19.428)	-		
Basurko et al 2022										Maternal age, gestational age at delivery, mode of delivery	Multivariate logistic regression (nested case-control)
Delayed neurodevelopment	1	135	51.5	-	135	8.1	-	11.977 (4.775 to 30.052)	-		
Gérardin et al 2014										Maternal age, gestational age at	Multivariate logistic regression (2-year follow-up cohort)
Congenital malformation	2	828	40.0	57	828	3.9	83	1.213 (0.679 to 2.169)	0		
Fritel et al 2010								1.269 (0.639 to 2.519)		Maternal age, parity, pre-existing medical conditions	Multivariate logistic regression
Basurko et al 2022								1.083 (0.362 to 3.235)		Maternal age, gestational age at delivery, pre-existing medical conditions	Multivariate logistic regression (nested case-control)
Death	1	655	0.8	-	655	1.2	-	0.619 (0.202 to 1.903)	-		
Fritel et al 2010										Maternal age, parity, mode of	Multivariate logistic regression

Note: Outcome definitions varied across studies; outcomes were grouped into broader categories for comparison.

Table S9. Publication bias analysis.

Analysis	*Egger*´s test p-value	*Begg*´s test p-value
Vertical transmission rate	0.3909	0.4620
Adverse pregnancy outcomes	0.2845	0.3127
Adverse neonatal outcomes	0.0674	0.1083
Risk of adverse outcomes	0.0787	0.1542

Table S10. Outcome definition mapping table.

Outcome Category	Specific Outcome	Study	Definition / Diagnostic Criteria Used	Notes
**Pregnancy Outcome**	Preterm birth	Lenglet et al 2006	Delivery before 37 weeks of gestation	-
		Gerardin et al 2008	Birth before 37 completed weeks	-
		Escobar et al 2017	Gestational age <37 weeks at delivery	-
		Basurko et al 2022	Delivery before 37 weeks of pregnancy	-
		Kumar et al 2019	Birth at <37 weeks gestation	-
		Torres et al 2016	Not specified	Assumed standard definition
		Sagay et al 2024	Preterm delivery (<37 weeks)	-
		Faria et al 2024	Birth before 37 weeks	-
**Pregnancy Outcome**	Stillbirth	Lenglet et al 2006	Fetal death after 20 weeks of gestation	-
		Touret et al 2006	Intrauterine fetal death after 22 weeks	-
		Senanayake et al 2009	Fetal death after 28 weeks of gestation	Different threshold
		Gerardin et al 2008	Fetal death at ≥20 weeks	-
**Pregnancy Outcome**	Miscarriage	Senanayake et al 2009	Pregnancy loss before 20 weeks	-
		Escobar et al 2017	Spontaneous abortion <20 weeks	-
		Pinheiro de Carvalho et al 2025	Pregnancy loss before 20-22 weeks	-
		Salomão et al 2021	Spontaneous abortion in first trimester	Pathologically confirmed
**Pregnancy Outcome**	Abnormal fetal heart rate	Gerardin et al 2008	Fetal heart rate abnormalities during labor	-
		Shrivastava et al 2011	Non-reassuring fetal heart rate pattern	-
		Enter et al 2018	Fetal distress with heart rate abnormalities	-
		Basurko et al 2022	Abnormal fetal heart rate on monitoring	-
**Pregnancy Outcome**	Fetal growth restriction	Senanayake et al 2009	Estimated fetal weight <10th percentile	-
		Escobar et al 2017	Intrauterine growth restriction on ultrasound	-
		Basurko et al 2022	Fetal weight <10th percentile for gestational age	-
		Nigam et al 2016	Fetal growth restriction on ultrasound	-
**Pregnancy Outcome**	Preeclampsia	Escobar et al 2017	Hypertension + proteinuria after 20 weeks	-
		Di Maio Ferreira et al 2024	Hypertension with end-organ dysfunction	-
		Oliveira et al 2018	Clinical diagnosis of preeclampsia	-
**Pregnancy Outcome**	Postpartum hemorrhage	Fritel et al 2010	Blood loss >500 mL vaginal, >1000 mL cesarean	-
		Escobar et al 2017	Excessive bleeding requiring intervention	-
		Basurko et al 2022	Estimated blood loss >500 mL	-
**Neonatal Outcome**	Preterm birth	Ramful et al 2007	Delivery <37 weeks	-

		Gérardin et al 2014	Gestational age <37 weeks	-
		Ramos et al 2018	Birth at 35 weeks	Specific case
		Kumar et al 2019	Preterm (<37 weeks)	-
**Neonatal Outcome**	Low birth weight	Di Maio Ferreira et al 2024	Birth weight <2500 g	-
**Neonatal Outcome**	Fever	Robillard et al 2006	Axillary temperature >37.5°C	-
		Gerardin et al 2008	Temperature >38.0°C	-
		Ramful et al 2007	Fever (temperature not specified)	-
		Torres et al 2016	Documented fever	-
		Maria et al 2018	Fever in neonatal period	-
		Corrêa et al 2020	Fever at presentation	-
**Neonatal Outcome**	Rash	Robillard et al 2006	Maculopapular rash	-
		Gerardin et al 2008	Exanthema	-
		Ramful et al 2007	Skin rash	-
		Torres et al 2016	Maculopapular or petechial rash	-
		Villamil-Gómez et al 2015	Maculopapular rash, bullous lesions	-
**Neonatal Outcome**	Pigmentation	Rao et al 2008	Hyperpigmentation	-
		Khandelwal et al 2012	Centro-facial hyperpigmentation	-
		Gopakumar et al 2012	Generalized hyperpigmentation	-
		Vasani et al 2016	Hyperpigmentation (brownie-nose)	-
		Torres et al 2016	Skin hyperpigmentation	-
**Neonatal Outcome**	Feeding difficulties	Gerardin et al 2008	Poor sucking, feeding refusal	-
		Enter et al 2018	Poor feeding	-
		Kumar et al 2019	Refusal to feed	-
		Goulart Corrêa et al 2020	Feeding difficulties	-
**Neonatal Outcome**	Abnormal crying	Robillard et al 2006	Irritability, high-pitched cry	-
		Gopakumar et al 2012	Abnormal cry	-
		Torres et al 2016	Irritability, excessive crying	-
		Corrêa et al 2020	Irritability	-
**Neonatal Outcome**	Thrombocytopenia	Robillard et al 2006	Platelet count <150 × 10^9/L	-
		Gerardin et al 2008	Platelet count <150,000/mm³	-
		Gopakumar et al 2012	Severe thrombocytopenia	-
		Torres et al 2016	Platelet count <100,000/mm³	Different threshold
		Maria et al 2018	Platelet count <150 × 10^9/L	-
		Kumar et al 2019	Platelet count <150,000/µL	-
		Corrêa et al 2020	Platelet count <150,000/mm³	-
**Neonatal Outcome**	Neurological complications	Shenoy et al 2012	Seizures, encephalopathy, cerebral palsy, developmental delay	Long-term follow-up
		Maria et al 2018	Seizures, encephalopathy, abnormal neuroimaging	-
		Corrêa et al 2020	Brain lesions on MRI, seizures	MRI findings
		de Souza et al 2024	Encephalitis, meningitis, seizures	Pathologically

				confirmed
		Kumar et al 2019	Seizures, encephalopathy	-
**Neonatal Outcome**	Seizures/Epilepsy	Villamil-Gómez et al 2015	Clinical seizures	-
		Bandeira et al 2016	Seizures, encephalitis	-
		Ramos et al 2018	Epilepsy, seizures	-
		Kumar et al 2019	Seizures	-
**Neonatal Outcome**	Brain lesions	Shenoy et al 2012	Brain parenchymal lesions on imaging	-
		Corrêa et al 2020	MRI: white matter lesions, cerebral atrophy	Detailed imaging
		Goulart Corrêa et al 2020	Brain lesions on neuroimaging	-
**Neonatal Outcome**	Respiratory distress	Rao et al 2008	Respiratory distress requiring support	-
		Senanayake et al 2009	Respiratory difficulty	-
		Shrivastava et al 2011	Respiratory distress	-
		Villamil-Gómez et al 2015	Respiratory failure, distress	-
		Torres et al 2016	Respiratory distress, failure	-
		Kumar et al 2019	Respiratory distress	-
**Neonatal Outcome**	Neonatal sepsis	Gopakumar et al 2012	Clinical sepsis	-
		Oliveira et al 2018	Clinical signs + positive blood culture	Culture-confirmed
		Kumar et al 2019	Clinical sepsis with or without positive culture	-
		Chandramathi et al 2020	Sepsis with thrombocytopenia	-
		Sagay et al 2024	Neonatal sepsis (clinical)	-
**Neonatal Outcome**	Myocarditis/Cardiac	Villamil-Gómez et al 2015	Myocarditis, pericardial effusion	Echo findings
		Nigam et al 2016	Pericardial effusion	-
		Kumar et al 2019	Myocarditis	-
**Neonatal Outcome**	Death	Fritel et al 2010	Neonatal death	-
		Villamil-Gómez et al 2015	Death in neonatal period	-
		Evans-Gilbert et al 2017	Fatal outcome	-
		Enter et al 2018	Neonatal death	-
		Oliveira et al 2018	Maternal and infant death	-
		Kumar et al 2019	Death in neonatal period	-
		Faria et al 2024	Neonatal death	-
		Pedrosa Do Monte et al 2025	Fetal or neonatal death	-

A detailed mapping of neonatal outcome definitions and their corresponding grouped categories is provided in Supplementary Table S10.

References

1 Lenglet Y, Barau G, Robillard PY, et al. [Chikungunya infection in pregnancy: Evidence for intrauterine infection in pregnant women and vertical transmission in the parturient. Survey of the Reunion Island outbreak]. *J Gynecol Obstet Biol Reprod (Paris)* 2006; **35**: 578-83.

2 Robillard PY, Boumahni B, Gérardin P, et al. [Vertical maternal fetal transmission of the chikungunya virus. Ten cases among 84 pregnant women]. *Presse Med* 2006; **35**: 785-88.

3 Watanaveeradej V, Endy TP, Simasathien S, et al. The study transplacental chikungunya virus antibody kinetics, Thailand. *Emerg Infect Dis* 2006; **12**: 1770-72.

4 Touret Y, Randrianaivo H, Michault A, et al. [Early maternal-fetal transmission of the Chikungunya virus]. *Presse Med* 2006; **35**: 1656-58.

5 Ramful D, Carbonnier M, Pasquet M, et al. Mother-to-child transmission of Chikungunya virus infection. *Pediatr Infect Dis J* 2007; **26**: 811-15.

6 Gérardin P, Barau G, Michault A, et al. Multidisciplinary prospective study of mother-to-child chikungunya virus infections on the island of La Réunion. *PLoS Med* 2008; **5**: e60.

7 Sissoko D, Malvy D, Giry C, et al. Outbreak of Chikungunya fever in Mayotte, Comoros archipelago, 2005-2006. *Trans R Soc Trop Med Hyg* 2008; **102**: 780-86.

8 Rao G, Khan YZ, Chitnis DS. Chikungunya infection in neonates. *Indian Pediatr* 2008; **45**: 240-42.

9 Senanayake MP, Senanayake SM, Vidanage KK, Gunasena S, Lamabadusuriya SP. Vertical transmission in chikungunya infection. *Ceylon Med J* 2009; **54**: 47-50.

10 Fritel X, Rollot O, Gerardin P, et al. Chikungunya virus infection during pregnancy, Reunion, France, 2006. *Emerg Infect Dis* 2010; **16**: 418-25.

11 Boumahni B, Kaplan C, Clabé A, Randrianaivo H, Lanza F. [Maternal-fetal chikungunya infection associated with Bernard-Soulier syndrome]. *Arch Pediatr* 2011; **18**: 272-75.

12 Shrivastava A, Waqar Beg M, Gujrati C, Gopalan N, Rao PV. Management of a vertically transmitted neonatal Chikungunya thrombocytopenia. *Indian J Pediatr* 2011; **78**: 1008-09.

13 Shenoy S, Pradeep GC. Neurodevelopmental outcome of neonates with vertically transmitted Chikungunya fever with encephalopathy. *Indian Pediatr* 2012; **49**: 238-40.

14 Khandelwal K, Aara N, Ghiya BC, Bumb RA, Satoskar AR. Centro-facial pigmentation in asymptomatic congenital chikungunya viral infection. *J Paediatr Child Health* 2012; **48**: 542-43.

15 Gopakumar H, Ramachandran S. Congenital chikungunya. *J Clin Neonatol* 2012; **1**: 155-56.

16 Kumar N, Gupta V, Thomas N. Brownie-nose: hyperpigmentation in neonatal chikungunya. *Indian Pediatr* 2014; **51**: 419.

17 Ramful D, Sampériz S, Fritel X, et al. Antibody kinetics in infants exposed to Chikungunya virus infection during pregnancy reveals absence of congenital infection. *J Infect Dis* 2014; **209**: 1726-30.

18 Gérardin P, Sampériz S, Ramful D, et al. Neurocognitive outcome of children exposed to perinatal mother-to-child Chikungunya virus infection: the CHIMERE cohort study on Reunion Island. *PLoS Negl Trop Dis* 2014; **8**: e2996.

19 Foeller ME, Nosrat C, Krystosik A, et al. Chikungunya infection in pregnancy - reassuring maternal and perinatal outcomes: a retrospective observational study. *BJOG* 2021; **128**: 1077-86.

20 Villamil-Gómez W, Alba-Silvera L, Menco-Ramos A, et al. Congenital Chikungunya Virus Infection in Sincelejo, Colombia: A Case Series. *J Trop Pediatr* 2015; **61**: 386-92.

21 Amar Taksande, K.Y.Vilhekar. Neonatal Chikungunya Infection. J Prev Infect Control. 2015. 258: 118171.

22 Vasani R, Kanhere S, Chaudhari K, et al. Congenital Chikungunya--A Cause of Neonatal Hyperpigmentation. *Pediatr Dermatol* 2016; **33**: 209-12.

23 Laoprasopwattana K, Suntharasaj T, Petmanee P, Suddeaugrai O, Geater A. Chikungunya and dengue virus infections during pregnancy: seroprevalence, seroincidence and maternal-fetal transmission, southern Thailand, 2009-2010. *Epidemiol Infect* 2016; **144**: 381-88.

24 Torres JR, Falleiros-Arlant LH, Dueñas L, Pleitez-Navarrete J, Salgado DM, Castillo JB. Congenital and perinatal complications of chikungunya fever: a Latin American experience. *Int J Infect Dis* 2016; **51**: 85-88.

25 Alvarado-Socarras JL, Ocampo-González M, Vargas-Soler JA, Rodriguez-Morales AJ, Franco-Paredes C. Congenital and Neonatal Chikungunya in Colombia. *J Pediatric Infect Dis Soc* 2016; **5**: e17-20.

26 Bandeira AC, Campos GS, Sardi SI, Rocha VF, Rocha GC. Neonatal encephalitis due to Chikungunya vertical transmission: First report in Brazil. *IDCases* 2016; **5**: 57-59.

27 Karthiga V, Kommu PP, Krishnan L. Perinatal chikungunya in twins. *J Pediatr Neurosci* 2016; **11**: 223-24.

28 Rodríguez-Nieves M, García-García I, García-Fragoso L. Perinatally Acquired Chikungunya Infection: The Puerto Rico Experience. *Pediatr Infect Dis J* 2016; **35**: 1163.

29 Nigam A, Sharma S, Jain A, Gupta A, Prakash A. Vertical Transmission of Chikungunya Manifesting as Foetal Pericardial Effusion. *J Assoc Physicians India* 2016; **64**: 76-79.

30 Lyra PP, Campos GS, Bandeira ID, et al. Congenital Chikungunya Virus Infection after an Outbreak in Salvador, Bahia, Brazil. *AJP Rep* 2016; **6**: e299-300.

31 Muñoz CM, Castillo JO, Salas D, Valderrama MA, Rangel CT, Vargas HP, et al. Atypical mucocutaneous manifestations in neonates and infants with chikungunya fever

in the municipalities of Cúcuta, Los Patios and Villa del Rosario, Norte de Santander, Colombia, 2014. Biomédica. 2016;36(3):368-77.

32 Escobar M, Nieto AJ, Loaiza-Osorio S, Barona JS, Rosso F. Pregnant Women Hospitalized with Chikungunya Virus Infection, Colombia, 2015. *Emerg Infect Dis* 2017; **23**: 1777-83.

33 Evans-Gilbert T. Chikungunya and Neonatal Immunity: Fatal Vertically Transmitted Chikungunya Infection. *Am J Trop Med Hyg* 2017; **96**: 913-15.

34 Cardona-Correa SE, Castaño-Jaramillo LM, Quevedo-Vélez A. [Vertical transmission of chikungunya virus infection. Case Report]. *Rev Chil Pediatr* 2017; **88**: 285-88.

35 Ramos R, Viana R, Brainer-Lima A, et al. Perinatal Chikungunya Virus-associated Encephalitis Leading to Postnatal-Onset Microcephaly and Optic Atrophy. *Pediatr Infect Dis J* 2018; **37**: 94-95.

36 Maria A, Vallamkonda N, Shukla A, Bhatt A, Sachdev N. Encephalitic presentation of Neonatal Chikungunya: A Case Series. *Indian Pediatr* 2018; **55**: 671-74.

37 Oliveira R, Barreto F, Maia A, et al. Maternal and infant death after probable vertical transmission of chikungunya virus in Brazil - case report. *BMC Infect Dis* 2018; **18**: 333.

38 van Enter B, Huibers M, van Rooij L, et al. Perinatal Outcomes in Vertically Infected Neonates During a Chikungunya Outbreak on the Island of Curaçao. *Am J Trop Med Hyg* 2018; **99**: 1415-18.

39 Dorléans F, Hoen B, Najioullah F, et al. Outbreak of Chikungunya in the French Caribbean Islands of Martinique and Guadeloupe: Findings from a Hospital-Based Surveillance System (2013-2015). *Am J Trop Med Hyg* 2018; **98**: 1819-25.

40 Kumar S, Agrawal G, Wazir S, et al. Experience of Perinatal and Neonatal Chikungunya Virus (CHIKV) Infection in a Tertiary Care Neonatal Centre during Outbreak in North India in 2016: A Case Series. *J Trop Pediatr* 2019; **65**: 169-75.

41 Di Maio Ferreira F, da Silva A, Bispo de Filippis AM, Brasil P. Late Identification of Chikungunya Virus in the Central Nervous System of a 2-Month-Old Infant: Persistence of Maternal-Neonatal Infection. *J Pediatric Infect Dis Soc* 2019; **8**: 374-77.

42 Chandramathi J, Prabhu A, Kumar A. The "Chik Sign" in Neonatal Chikungunya. *Rev Soc Bras Med Trop* 2020; **53**: e20200157.

43 Corrêa DG, Freddi T, Werner H, et al. Brain MR Imaging of Patients with Perinatal Chikungunya Virus Infection. *AJNR Am J Neuroradiol* 2020; **41**: 174-77.

44 Jebain J, Siller A Jr, Lupi O, et al. Perinatal chikungunya induced scalded skin syndrome. *IDCases* 2020; **22**: e00969.

45 Corrêa DG, Di Maio Ferreira F, Hygino da Cruz LC Jr, Brasil P, Rueda Lopes FC. Longitudinal brain magnetic resonance imaging of children with perinatal Chikungunya encephalitis. *Neuroradiol J* 2020; **33**: 532-37.

46 Shen JY, Li M, Xie L, et al. Perinatal Vertical Transmission of Chikungunya Virus in Ruili, a Town on the Border between China and Myanmar. *Virol Sin* 2021; **36**: 145-48.

47 Salomão N, Brendolin M, Rabelo K, et al. Spontaneous Abortion and Chikungunya Infection: Pathological Findings. *Viruses* 2021; **13**: 554.

48 Basurko C, Hcini N, Demar M, et al. Symptomatic Chikungunya Virus Infection and Pregnancy Outcomes: A Nested Case-Control Study in French Guiana. *Viruses* 2022; **14**: 2705.

49 Salomão N, Araújo L, Rabelo K, et al. Placental Alterations in a Chikungunya-Virus-Infected Pregnant Woman: A Case Report. *Microorganisms* 2022; **10**: 872.

50 Ferreira F, Filippis A, Moreira M, et al. Perinatal and Neonatal Chikungunya Virus Transmission: A Case Series. *J Pediatric Infect Dis Soc* 2024; **13**: 576-84.

51 Ngwe Tun MM, Luvai E, Toizumi M, et al. Possible vertical transmission of Chikungunya virus infection detected in the cord blood samples from a birth cohort in Vietnam. *J Infect Public Health* 2024; **17**: 1050-56.

52 Sagay AS, Hsieh SC, Dai YC, et al. Chikungunya virus antepartum transmission and abnormal infant outcomes in a cohort of pregnant women in Nigeria. *Int J Infect Dis* 2024; **139**: 92-100.

53 Faria BS, da Silva LB, Avelar C, de Morais P, Bentes AA. Vertical transmission of chikungunya virus: a worldwide concern. *Braz J Infect Dis* 2024; **28**: 103747.

54 Do Monte A, Lacerda HR. Fetal and Neonatal Deaths Resulting from Chikungunya Virus Infection During Pregnancy: A Case Series. *Int Med Case Rep J* 2025; **18**: 479-85.

55 de Carvalho A, Cruz A, Quaresma J, et al. Impact of Zika and Chikungunya Viruses on Spontaneous Abortions: Insights from a Reference Maternity Hospital. *Microorganisms* 2025; **13**: 678.

56 Pinho de Almeida Di Maio Ferreira FC, Nielsen-Saines K, Lopes Moreira ME, et al. Neurodevelopmental Follow-Up in Children with Intrauterine and Perinatal Exposure to Chikungunya Virus. *J Pediatr* 2025; **279**: 114477.

57 Yin X, Hu TS, Zhang H, et al. Emergent chikungunya fever and vertical transmission in Yunnan Province, China, 2019. *Arch Virol* 2021; **166**: 1455-62.
