# Supplementary material for: Maternal Chikungunya virus infection and pregnancy outcomes: a global systematic review and meta-analysis of vertical transmission dynamics and associated morbidity
Source: Emerg Microbes Infect. 2026 Mar 25;15(1):2651466. doi: 10.1080/22221751.2026.2651466 (PMC13101005; doi:10.1080/22221751.2026.2651466)

Study	Proportion	95%−CI	P−value	Tau2	Tau	I2
Omitting Robillard	0.37	[0.30; 0.43]	.	0.1624	0.4030	96%
Omitting Ramful	0.36	[0.30; 0.43]	.	0.1616	0.4019	96%
Omitting Gerardin	0.36	[0.29; 0.42]	.	0.1568	0.3960	96%
Omitting Senanayake	0.36	[0.30; 0.43]	.	0.1613	0.4016	96%
Omitting Torres	0.36	[0.29; 0.42]	.	0.1562	0.3953	95%
Omitting Rodríguez−Nieves	0.36	[0.30; 0.43]	.	0.1630	0.4037	96%
Omitting Muñoz	0.36	[0.29; 0.43]	.	0.1574	0.3968	96%
Omitting Maria	0.36	[0.30; 0.43]	.	0.1612	0.4015	96%
Omitting Kumar	0.36	[0.30; 0.43]	.	0.1623	0.4029	96%
Omitting Corrêa	0.36	[0.30; 0.43]	.	0.1620	0.4025	96%
Omitting Basurko	0.37	[0.30; 0.43]	.	0.1629	0.4037	96%
Omitting Di Maio Ferreira	0.37	[0.30; 0.43]	.	0.1630	0.4038	96%
Omitting Ferreira	0.37	[0.30; 0.43]	.	0.1630	0.4037	96%
Omitting Robillard	0.36	[0.29; 0.43]	.	0.1575	0.3969	96%
Omitting Ramful	0.36	[0.30; 0.43]	.	0.1613	0.4016	96%
Omitting Gerardin	0.36	[0.30; 0.43]	.	0.1605	0.4006	96%
Omitting Villamil−Gómez	0.36	[0.30; 0.43]	.	0.1626	0.4032	96%
Omitting Torres	0.36	[0.30; 0.43]	.	0.1609	0.4012	96%
Omitting Torres	0.36	[0.30; 0.43]	.	0.1586	0.3982	96%
Omitting Torres	0.36	[0.30; 0.43]	.	0.1632	0.4039	96%
Omitting Rodríguez−Nieves	0.36	[0.30; 0.43]	.	0.1627	0.4034	96%
Omitting Muñoz	0.36	[0.30; 0.43]	.	0.1605	0.4006	96%
Omitting Kumar	0.37	[0.30; 0.43]	.	0.1630	0.4038	96%
Omitting Di Maio Ferreira	0.37	[0.30; 0.43]	.	0.1629	0.4036	96%
Omitting Ferreira	0.37	[0.30; 0.43]	.	0.1629	0.4036	96%
Omitting Senanayake	0.37	[0.30; 0.43]	.	0.1631	0.4038	96%
Omitting Torres	0.37	[0.30; 0.44]	.	0.1618	0.4023	96%
Omitting Maria	0.36	[0.30; 0.43]	.	0.1618	0.4023	96%
Omitting Di Maio Ferreira	0.37	[0.30; 0.43]	.	0.1625	0.4032	96%
Omitting Ferreira	0.37	[0.30; 0.43]	.	0.1625	0.4031	96%
Omitting Villamil−Gómez	0.37	[0.30; 0.43]	.	0.1626	0.4032	96%
Omitting Torres	0.37	[0.30; 0.43]	.	0.1625	0.4031	96%
Omitting Torres	0.37	[0.30; 0.44]	.	0.1623	0.4028	96%
Omitting Di Maio Ferreira	0.37	[0.30; 0.43]	.	0.1625	0.4032	96%
Omitting Ferreira	0.37	[0.30; 0.43]	.	0.1625	0.4031	96%
Omitting Robillard	0.36	[0.29; 0.43]	.	0.1575	0.3969	96%
Omitting Ramful	0.36	[0.30; 0.43]	.	0.1628	0.4035	96%
Omitting Gerardin	0.36	[0.30; 0.43]	.	0.1616	0.4020	96%
Omitting Foeller	0.37	[0.30; 0.44]	.	0.1612	0.4015	96%
Omitting Villamil−Gómez	0.37	[0.30; 0.43]	.	0.1629	0.4037	96%
Omitting Torres	0.36	[0.30; 0.43]	.	0.1607	0.4009	96%
Omitting Torres	0.37	[0.30; 0.43]	.	0.1631	0.4039	96%
Omitting Torres	0.36	[0.30; 0.43]	.	0.1632	0.4039	96%
Omitting Rodríguez−Nieves	0.37	[0.30; 0.43]	.	0.1630	0.4037	96%
Omitting Gerardin	0.36	[0.29; 0.42]	.	0.1568	0.3960	96%
Omitting Sissoko	0.36	[0.30; 0.43]	.	0.1628	0.4035	96%
Omitting Villamil−Gómez	0.36	[0.30; 0.43]	.	0.1629	0.4036	96%
Omitting Torres	0.36	[0.30; 0.43]	.	0.1594	0.3992	96%
Omitting Torres	0.37	[0.30; 0.43]	.	0.1626	0.4033	96%
Omitting Torres	0.36	[0.30; 0.43]	.	0.1628	0.4035	96%
Omitting Rodríguez−Nieves	0.36	[0.30; 0.43]	.	0.1630	0.4037	96%
Omitting Gerardin	0.36	[0.29; 0.42]	.	0.1568	0.3960	96%
Omitting Rodríguez−Nieves	0.37	[0.30; 0.43]	.	0.1630	0.4037	96%
Omitting Maria	0.36	[0.29; 0.43]	.	0.1572	0.3965	96%
Omitting Kumar	0.36	[0.30; 0.43]	.	0.1631	0.4039	96%
Omitting Robillard	0.36	[0.29; 0.43]	.	0.1575	0.3969	96%
Omitting Torres	0.36	[0.30; 0.43]	.	0.1601	0.4002	96%
Omitting Rodríguez−Nieves	0.36	[0.30; 0.43]	.	0.1627	0.4034	96%
Omitting Muñoz	0.36	[0.30; 0.43]	.	0.1621	0.4026	96%
Omitting Corrêa	0.37	[0.30; 0.43]	.	0.1626	0.4032	96%
Omitting Di Maio Ferreira	0.37	[0.30; 0.43]	.	0.1625	0.4032	96%
Omitting Ramful	0.37	[0.30; 0.43]	.	0.1632	0.4039	96%
Omitting Torres	0.37	[0.30; 0.43]	.	0.1629	0.4036	96%
Omitting Torres	0.37	[0.30; 0.44]	.	0.1623	0.4028	96%
Omitting Kumar	0.37	[0.30; 0.44]	.	0.1621	0.4026	96%
Omitting Robillard	0.36	[0.29; 0.43]	.	0.1575	0.3969	96%
Omitting Gerardin	0.36	[0.30; 0.43]	.	0.1605	0.4006	96%
Omitting Torres	0.37	[0.30; 0.44]	.	0.1616	0.4020	96%
Omitting Rodríguez−Nieves	0.37	[0.30; 0.43]	.	0.1630	0.4037	96%
Omitting Maria	0.36	[0.30; 0.43]	.	0.1602	0.4003	96%
Omitting Kumar	0.36	[0.30; 0.43]	.	0.1630	0.4037	96%
Omitting Corrêa	0.37	[0.30; 0.43]	.	0.1626	0.4032	96%
Omitting Senanayake	0.37	[0.30; 0.43]	.	0.1626	0.4033	96%
Omitting Villamil−Gómez	0.36	[0.30; 0.43]	.	0.1629	0.4036	96%
Omitting Torres	0.37	[0.30; 0.44]	.	0.1622	0.4028	96%
Omitting Torres	0.37	[0.30; 0.44]	.	0.1624	0.4030	96%
Omitting Rodríguez−Nieves	0.36	[0.30; 0.43]	.	0.1630	0.4037	96%
Omitting Kumar	0.36	[0.30; 0.43]	.	0.1631	0.4039	96%
Omitting Basurko	0.37	[0.30; 0.43]	.	0.1626	0.4032	96%
Omitting Di Maio Ferreira	0.37	[0.30; 0.43]	.	0.1629	0.4036	96%
Omitting Ferreira	0.37	[0.30; 0.43]	.	0.1629	0.4036	96%
Omitting Torres	0.37	[0.30; 0.44]	.	0.1622	0.4027	96%
Omitting Rodríguez−Nieves	0.36	[0.30; 0.43]	.	0.1630	0.4037	96%
Omitting Maria	0.37	[0.30; 0.43]	.	0.1627	0.4034	96%
Omitting Di Maio Ferreira	0.37	[0.30; 0.44]	.	0.1623	0.4028	96%
Omitting Ferreira	0.37	[0.30; 0.43]	.	0.1625	0.4031	96%
Omitting Robillard	0.36	[0.30; 0.43]	.	0.1630	0.4038	96%
Omitting Gerardin	0.36	[0.30; 0.43]	.	0.1630	0.4038	96%
Omitting Sissoko	0.37	[0.30; 0.43]	.	0.1630	0.4038	96%
Omitting Senanayake	0.37	[0.30; 0.43]	.	0.1626	0.4033	96%
Omitting Villamil−Gómez	0.37	[0.30; 0.43]	.	0.1629	0.4037	96%
Omitting Torres	0.37	[0.30; 0.44]	.	0.1622	0.4027	96%
Omitting Torres	0.37	[0.30; 0.44]	.	0.1609	0.4012	96%
Omitting Rodríguez−Nieves	0.36	[0.30; 0.43]	.	0.1596	0.3995	96%
Omitting Maria	0.37	[0.30; 0.43]	.	0.1630	0.4037	96%
Omitting Kumar	0.37	[0.30; 0.43]	.	0.1629	0.4036	96%
Omitting Corrêa	0.36	[0.30; 0.43]	.	0.1620	0.4025	96%
Omitting Di Maio Ferreira	0.37	[0.30; 0.43]	.	0.1631	0.4038	96%
Omitting Torres	0.37	[0.30; 0.44]	.	0.1614	0.4018	96%
Omitting Kumar	0.36	[0.30; 0.43]	.	0.1630	0.4037	96%
Omitting Corrêa	0.36	[0.30; 0.43]	.	0.1630	0.4037	96%
Omitting Basurko	0.37	[0.30; 0.44]	.	0.1610	0.4012	96%
Omitting Basurko	0.37	[0.30; 0.44]	.	0.1606	0.4007	96%
Omitting Di Maio Ferreira	0.37	[0.30; 0.43]	.	0.1628	0.4034	96%
Omitting Ferreira	0.37	[0.30; 0.43]	.	0.1627	0.4034	96%
Omitting Maria	0.36	[0.30; 0.43]	.	0.1630	0.4037	96%
Omitting Villamil−Gómez	0.36	[0.30; 0.43]	.	0.1630	0.4037	96%
Omitting Corrêa	0.37	[0.30; 0.43]	.	0.1626	0.4032	96%
Omitting Fritel	0.37	[0.30; 0.44]	.	0.1614	0.4017	96%
Omitting Foeller	0.37	[0.30; 0.44]	.	0.1612	0.4015	96%
Omitting Escobar	0.37	[0.30; 0.43]	.	0.1623	0.4028	96%
Omitting Maria	0.37	[0.30; 0.44]	.	0.1622	0.4028	96%
Omitting Basurko	0.37	[0.30; 0.44]	.	0.1621	0.4026	96%
Omitting Sagay	0.37	[0.30; 0.44]	.	0.1617	0.4021	96%
Omitting Villamil−Gómez	0.36	[0.30; 0.43]	.	0.1626	0.4032	96%
Omitting Torres	0.37	[0.30; 0.44]	.	0.1612	0.4015	96%
Omitting Torres	0.37	[0.30; 0.44]	.	0.1619	0.4024	96%
Omitting Torres	0.37	[0.30; 0.44]	.	0.1609	0.4012	96%
Omitting Kumar	0.37	[0.30; 0.43]	.	0.1629	0.4036	96%
Omitting Senanayake	0.37	[0.30; 0.43]	.	0.1629	0.4036	96%
Omitting Kumar	0.37	[0.30; 0.43]	.	0.1630	0.4038	96%
Omitting Kumar	0.37	[0.30; 0.43]	.	0.1629	0.4036	96%
Omitting Sissoko	0.37	[0.30; 0.43]	.	0.1625	0.4031	96%
Omitting Maria	0.36	[0.29; 0.43]	.	0.1572	0.3965	96%
Omitting Ferreira	0.37	[0.30; 0.43]	.	0.1631	0.4038	96%
Omitting Sissoko	0.37	[0.30; 0.43]	.	0.1629	0.4036	96%
Omitting Dorléans	0.37	[0.30; 0.43]	.	0.1630	0.4038	96%
Omitting Sagay	0.37	[0.30; 0.44]	.	0.1617	0.4021	96%
Omitting Senanayake	0.37	[0.30; 0.43]	.	0.1626	0.4033	96%
Omitting Kumar	0.37	[0.30; 0.43]	.	0.1626	0.4032	96%
Omitting Gérardin	0.36	[0.30; 0.43]	.	0.1630	0.4037	96%
Omitting Maria	0.37	[0.30; 0.43]	.	0.1631	0.4038	96%
Omitting Rodríguez−Nieves	0.37	[0.30; 0.43]	.	0.1630	0.4037	96%
Omitting Kumar	0.37	[0.30; 0.44]	.	0.1621	0.4026	96%
Omitting Senanayake	0.37	[0.30; 0.43]	.	0.1626	0.4033	96%
Omitting Villamil−Gómez	0.36	[0.30; 0.43]	.	0.1630	0.4037	96%
Omitting Maria	0.36	[0.29; 0.43]	.	0.1572	0.3965	96%
Omitting Kumar	0.36	[0.30; 0.43]	.	0.1628	0.4035	96%
Omitting Corrêa	0.37	[0.30; 0.43]	.	0.1629	0.4037	96%
Omitting Fritel	0.37	[0.30; 0.44]	.	0.1607	0.4008	95%
Omitting Villamil−Gómez	0.36	[0.30; 0.43]	.	0.1630	0.4037	96%
Omitting Torres	0.37	[0.30; 0.44]	.	0.1618	0.4023	96%
Omitting Kumar	0.37	[0.30; 0.44]	.	0.1621	0.4026	96%
Omitting Sagay	0.37	[0.30; 0.43]	.	0.1625	0.4031	96%
**Random effects model**	**0.36**	**[0.30; 0.43]**	**.**	**0.1619**	**0.4023**	**96%**

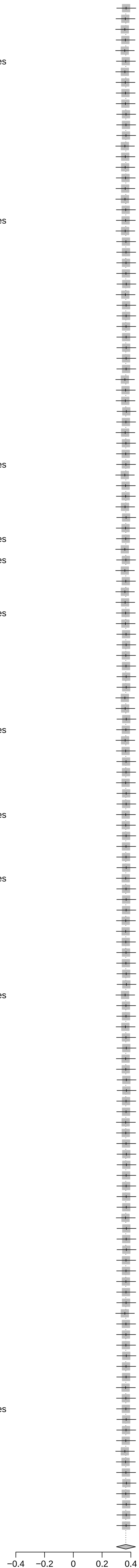

Supplement: FigureS13.pdf [file TEMI_A_2651466_SM5562.pdf]
